# SDS‐CRISPR for Single‐Nucleotide Variant Detection

**DOI:** 10.1002/advs.75149

**Published:** 2026-04-07

**Authors:** Xin Guan, Chong Guo, Jiongyu Zhang, Rui Yang, Yerramsetti Ramachandra, Chengyu Hou, Minjie Pei, Shuo Zhang, Kurt T. Schalper, Xingye Liu, Qian Wu, Ketan R. Bulsara, Changchun Liu

**Affiliations:** ^1^ Department of Biomedical Engineering University of Connecticut Health Center Farmington Connecticut USA; ^2^ Department of Biomedical Engineering University of Connecticut Storrs Connecticut USA; ^3^ Pathology and Laboratory Medicine University of Connecticut Health Center Farmington Connecticut USA; ^4^ Department of Neurosurgery University of Connecticut Health Center Farmington Connecticut USA

**Keywords:** CRISPR‐based molecular diagnostics, glioma diagnosis, IDH1 mutation, SDS‐CRISPR, single‐nucleotide variant detection

## Abstract

The CRISPR‐Cas12a system offers a promising platform for simple and sensitive nucleic acid diagnostics, including tumor‐associated variant detection and infectious agent identification. However, its intrinsic mismatch tolerance limits its ability to accurately detect single‐nucleotide variants (SNVs). Here, we introduce **S** tructure‐**D** isruption‐**S** ensitive CRISPR (SDS‐CRISPR), a programmable CRISPR‐Cas12a approach that achieves highly precise allele discrimination. Guided by AlphaFold3 modeling and bioinformatic analysis, we uncover how split structural design and ionic modulation reconfigure Cas12a conformations, elucidating the structural basis of SNV discrimination in SDS‐CRISPR. We apply SDS‐CRISPR to detect IDH1^WT^ and IDH1^R132H^ alleles with attomole sensitivity and 0.01% variant frequency. To facilitate intraoperative use, we combine SDS‐CRISPR with a lateral‐flow strip and an artificial intelligence‐assisted smartphone reader, enabling on‐site detection within 20 min. Clinical validation with 43 glioma tissue samples shows high concordance with immunohistochemistry, while plasma cfDNA testing demonstrates mutation fractions consistent with next‐generation sequencing. Beyond glioma, SDS‐CRISPR generalizes across molecular targets, discriminating microRNA isoforms and identifying HIV‐1 drug‐resistance mutations. Together, these results establish SDS‐CRISPR as a universal, mechanistically informed, and clinically actionable framework for precision molecular diagnostics.

## Graphical abstract

Structure‐disruption‐sensitive CRISPR (SDS‐CRISPR) converts structural instability into single‐nucleotide precision, thereby overcoming mismatch tolerance in canonical Cas12a and enabling versatile diagnostics across DNA, RNA, and microRNA targets. When applied to rapid IDH1 mutation detection for glioma genotyping and integrated with lateral‐flow strips and AI‐enabled smartphone readout, it delivers onsite detection within 20 min to support intraoperative decision‐making.

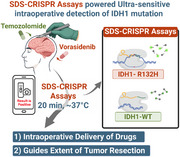

## 1 Introduction

The CRISPR‐Cas12a system has become a powerful tool for nucleic acid diagnostics [1, 2], enabling the detection of infectious agents and tumor‐associated mutations [3]. However, canonical CRISPR‐Cas12a assays show limited ability to discriminate single‐nucleotide variants (SNVs), as the highly stable full‐sized CRISPR RNA (crRNA)–target duplex often fails to prevent Cas12a activation in the presence of mismatches, leading to substantial non‐specific signals. Efforts to improve SNV specificity, such as introducing synthetic mismatches [4, 5, 6] or chemical modifications [4, 5, 6], have achieved partial success but typically require extensive optimization and remain limited by sequence context.

Recent advances in CRISPR‐Cas12a engineering have highlighted the potential of split and structurally modified designs for improving SNV discrimination. For example, Hu et al. used spatial blocking within the CRISPR‐Cas12a system to achieve ultrasensitive small molecule detection [7]. Moreover, the “splice‐at‐will” approach [8] and the split‐activator strategy [9] demonstrated that introducing breaks into CRISPR components enhances single‐base resolution for ultrashort RNA and microRNA (miRNA) detection, respectively. Chen et al. reported that implementing a split‐crRNA configuration enhances the discrimination capability of DNA assays [10]. Despite these promising advances, most studies remain at the proof‐of‐concept stage without clinical validation, primarily due to the challenge posed by the abundant background DNA or RNA present in clinical samples [9]. More importantly, current approaches are often limited by target type, split configuration, or the need for extensive empirical optimization for each new target. The underlying structure‐disruption‐sensitive mechanism of Cas12a remains unclear, hindering the establishment of a generalizable and programmable design principle.

Gliomas are aggressive brain tumors whose clinical management increasingly depends on the mutational status of the isocitrate dehydrogenase (IDH) genes, which serve as key molecular markers according to the 2021 World Health Organization Classification of Tumors of the Central Nervous System [11, 12]. IDH mutations not only stratify patients by prognosis but also dictate intraoperative resection strategies and thereby critically determine survival outcomes [13, 14]. Among these alterations, IDH1^R132H^ is the most prevalent, accounting for ∼90% of all IDH mutations in diffuse gliomas [15], and represents the most clinically actionable variant with established diagnostic and therapeutic relevance [13, 16, 17, 18, 19, 20]. Rapid intraoperative identification of IDH1^R132H^ is therefore essential for precision neurosurgical care. Beyond surgical guidance, such detection could further enable mutation‐directed local interventions when paired with sustained‐release drug delivery technologies to achieve better tumor control with reduced systemic toxicity [21, 22, 23, 24, 25, 26, 27, 28, 29]. However, conventional methods such as next‐generation sequencing (NGS) and immunohistochemistry (IHC) have long turnaround times incompatible with intraoperative decision‐making [30], highlighting the urgent need for simple, rapid, point‐of‐care molecular diagnostics in the neurosurgical theater.

Here, we present **S** tructure‐**D** isruption‐**S** ensitive CRISPR (SDS‐CRISPR), a programmable and structurally responsive CRISPR‐Cas12a platform that establishes a generalizable principle for SNV discrimination. Through structure‐disruptive nicks in either the DNA activator or the crRNA, SDS‐CRISPR abolishes cross‐activation between wild‐type and mutant alleles, achieving highly sensitive and specific detection across DNA and RNA targets. AlphaFold3 modeling and bioinformatic analysis further reveal that split design and ionic modulation reconfigure CRISPR‐Cas12a conformations, providing mechanistic insight into the structural determinants of SNV specificity. Leveraging this framework and an improved mechanistic understanding of Cas12a, we implement SDS‐CRISPR to develop an intraoperative assay that integrates a lateral‐flow strip with an artificial intelligence (AI)‐powered smartphone application for detecting IDH1^R132H^ mutations in clinical glioma samples within 20 min, offering a simple, rapid, and reliable approach to guide surgical and therapeutic decisions. Beyond glioma, SDS‐CRISPR reliably discriminates closely related microRNA family members and selectively identifies the HIV‐1 reverse transcriptase M184V drug‐resistance mutation, underscoring its versatility across nucleic acid targets. Collectively, SDS‐CRISPR introduces a universal, structurally programmable, mechanistically informed, and clinically actionable framework with broad potential for precision diagnostics.

## 2 Results

### 2.1 Overview of the SDS‐CRISPR System

**FIGURE 1:**
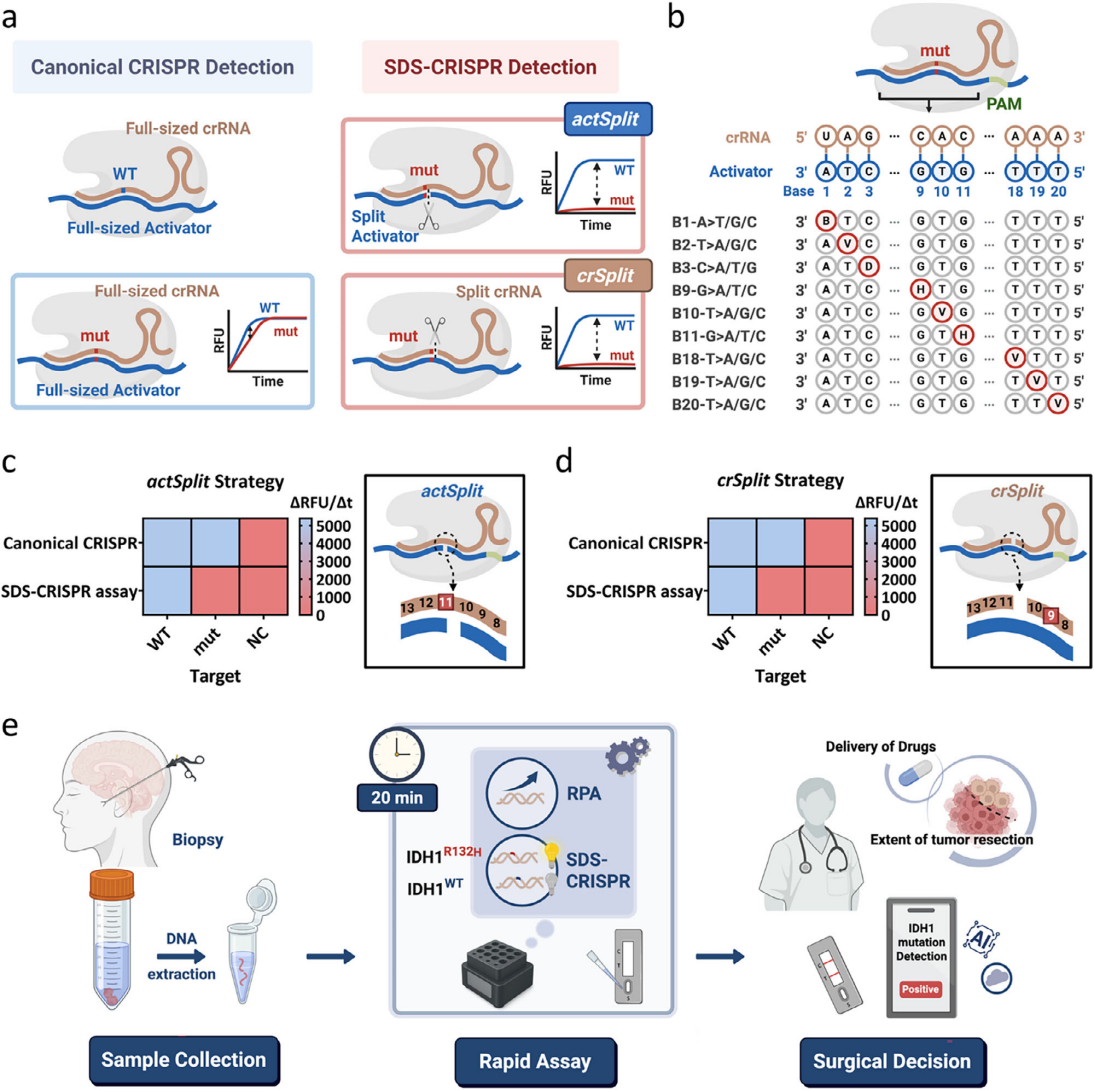
Overview of the SDS‐CRISPR system. (a) Schematic of detection strategies for canonical CRISPR and SDS‐CRISPR systems. WT, wild type; mut, mutant. (b) Design of a mutant library introducing single‐base substitutions at 20 positions across the crRNA recognition region. At each position, three possible base substitutions were introduced using standard degenerate nucleotide codes: B (C, G, or T), V (A, C, or G), D (A, G, or T), and H (A, C, or T). Nine representative sites (B1–3, 9–11, 18–20) were selected for analysis. (c, d) Heatmaps showing the results of the *actSplit* strategy at the B11 position (c) and the *crSplit* strategy at the B9 position (d), comparing canonical CRISPR and SDS‐CRISPR assays for wild‐type (WT), mutant (mut), and negative control (NC) targets. (e) Implementation of the SDS‐CRISPR IDH1 assay within a clinical workflow, enabling intraoperative mutation detection and therapeutic guidance.

The canonical CRISPR‐Cas12a system fails to discriminate SNVs because full‐sized crRNA–DNA activator (or target) duplexes tolerate mismatches (Figures S1 and S2). To address this limitation, we investigated SDS‐CRISPR, which introduces engineered structural breaks to destabilize local base pairing near the variant site, thereby providing a structurally programmable framework for precise SNV detection (Figure 1a). We developed two SDS‐CRISPR strategies: (i) *actSplit (activator split)*, which introduces a structural nick in the DNA activator strand, and (ii) *crSplit (crRNA split)*, which embeds a discontinuity directly into the crRNA. Together, these strategies provide complementary modes of control, enabling flexible positioning of structural disruptions and robust discrimination between wild‐type and mutant targets.

To systematically evaluate this principle, we constructed a mutant library containing all three possible base substitutions at 20 positions across the crRNA recognition region, allowing positional specificity and the performance of *actSplit* vs. *crSplit* to be systematically compared (Figure 1b). Each position was designated B1–B20 from the protospacer adjacent motif (PAM)‐proximal to distal end (activator 3’ to 5’, crRNA 5’ to 3’). At representative loci, SDS‐CRISPR consistently suppressed mutant signals to near‐background levels while preserving strong wild‐type activity, as shown for *actSplit* at B11 (Figure 1c) and *crSplit* at B9 (Figure 1d). These results demonstrate that SDS‐CRISPR eliminates the cross‐activation that compromises canonical CRISPR‐Cas12a, thereby achieving unambiguous, high‐fidelity discrimination between wild‐type and mutant alleles.

With its high‐fidelity discrimination, positional flexibility, and robustness against cross‐activation, SDS‐CRISPR offers a structurally programmable assay architecture intrinsically suited for clinical translation and aligned with the stringent requirements of precision diagnostics. For clinical implementation, we established a workflow in which surgical biopsy specimens are processed by DNA extraction (sample collection), followed by recombinase polymerase amplification (RPA) and SDS‐CRISPR analysis (rapid assay). Results are displayed on a lateral‐flow strip and interpreted through a smartphone application featuring AI‐powered analysis and secure cloud reporting. This readout provides intraoperative guidance for both drug delivery and the extent of tumor resection (surgical decision) (Figure 1e). The rapid assay is completed within 20 min, satisfying intraoperative time constraints, and the integrated workflow establishes SDS‐CRISPR as a clinically actionable platform for precision oncology.

### 2.2 *actSplit* Enables Precise SNV Discrimination

**FIGURE 2:**
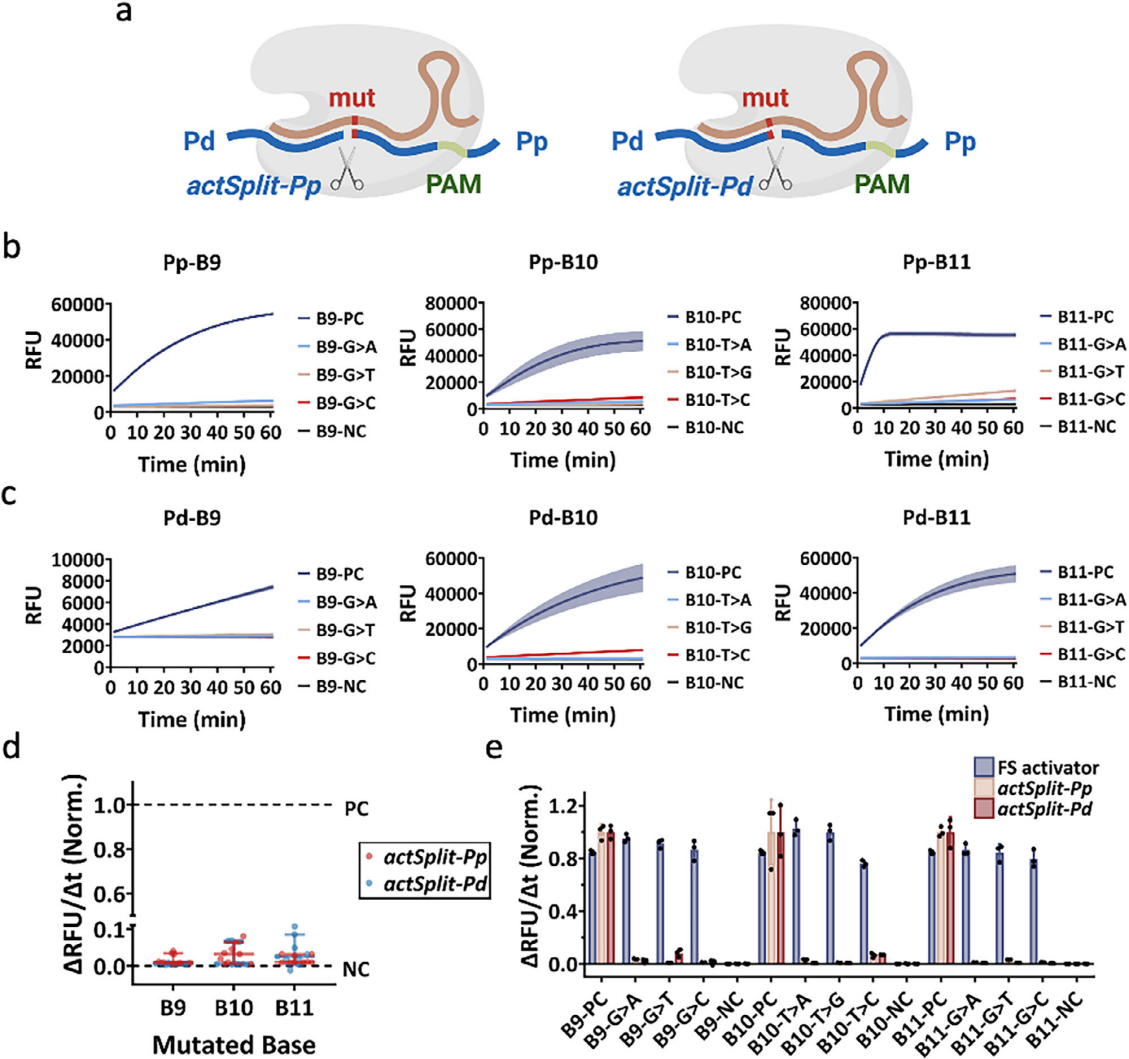
*actSplit* enables precise SNV discrimination. (a) Schematic of the *actSplit* strategy, in which the activator is cleaved at the SNV site to generate two designs: *actSplit‐Pp* and *actSplit‐Pd*, depending on whether the SNV resides on the Pp or Pd fragment. (b,c) Real‐time fluorescence kinetics of Cas12a cleavage for three SNV positions (B9, B10, B11) with the SNV placed on Pp (b) or Pd (c). Each graph includes the split activator perfect‐match control (PC), three SNV variants, and a no‐template control (NC, lacking the fragment harboring the SNV). Data represent mean ± s.d. from three technical replicates (*n* = 3). (d) Normalized fluorescence growth rates (0–5 min) for SNVs at positions B9, B10, and B11 with the variant placed on the Pp or Pd fragment. Each group comprises three SNV types with three replicates each (*n* = 9). Data are shown as the median with 95% confidence intervals. Dashed lines indicate the reference levels for the split activator perfect‐match control (PC) and the no‐template control (NC, lacking the fragment harboring the SNV). (e) Normalized fluorescence growth rates (0–5 min) for SNVs at positions B9, B10, and B11 under three detection designs: full‐sized activator (FS activator), *actSplit‐Pp*, and *actSplit‐Pd*. For each design, mutant signals were normalized to the corresponding wild‐type reference. Each bar represents the mean ± s.d. from three replicates (*n* = 3).

To examine the effect of cleavage placement on SNV discrimination, we applied the *actSplit* strategy to the DNA activator, placing the cleavage junction adjacent to the variant and anchoring the SNV at the split boundary on the 5’ end of the PAM‐proximal (Pp) fragment; this design is readily compatible with assays that permit exogenous activator input. By shifting the junction along the crRNA–activator duplex, the variant was systematically repositioned across different sites of the hybridization interface (Figure 2a). In this configuration, Cas12a activation in response to mutant targets was markedly attenuated (Figure 2b; Figures S3 and S5a). The strongest suppression occurred when the SNV was near the central region (B9–B11), whereas effects were less pronounced at the duplex termini.

To assess the impact of junction polarity, we inverted the configuration, placing the SNV on the 3’ end of the PAM‐distal (Pd) fragment (Figure 2a). Similar to *actSplit‐Pp*, the *actSplit‐Pd* design markedly suppressed activation by mismatched targets across multiple positions (Figure 2c; Figures S4 and S5b). This finding was further supported by aggregated analysis across mutation types, in which mutant signals at each site were normalized to the corresponding wild‐type split activator (Figure 2d). We next compared both *actSplit* configurations with the full‐sized activator. Although the split designs slightly reduced wild‐type signal (Figure S6), normalization revealed a clear gain in discrimination (Figure 2e), demonstrating that engineered structural breaks trade modest activity loss for substantial improvements in SNV resolution.

Together, *actSplit* establishes a robust framework for precise single‐nucleotide discrimination in CRISPR‐Cas12a detection by selectively suppressing mismatch‐driven activation and amplifying the contrast between wild‐type and mutant responses.

### 2.3 *crSplit* Delivers Enhanced SNV Specificity

**FIGURE 3:**
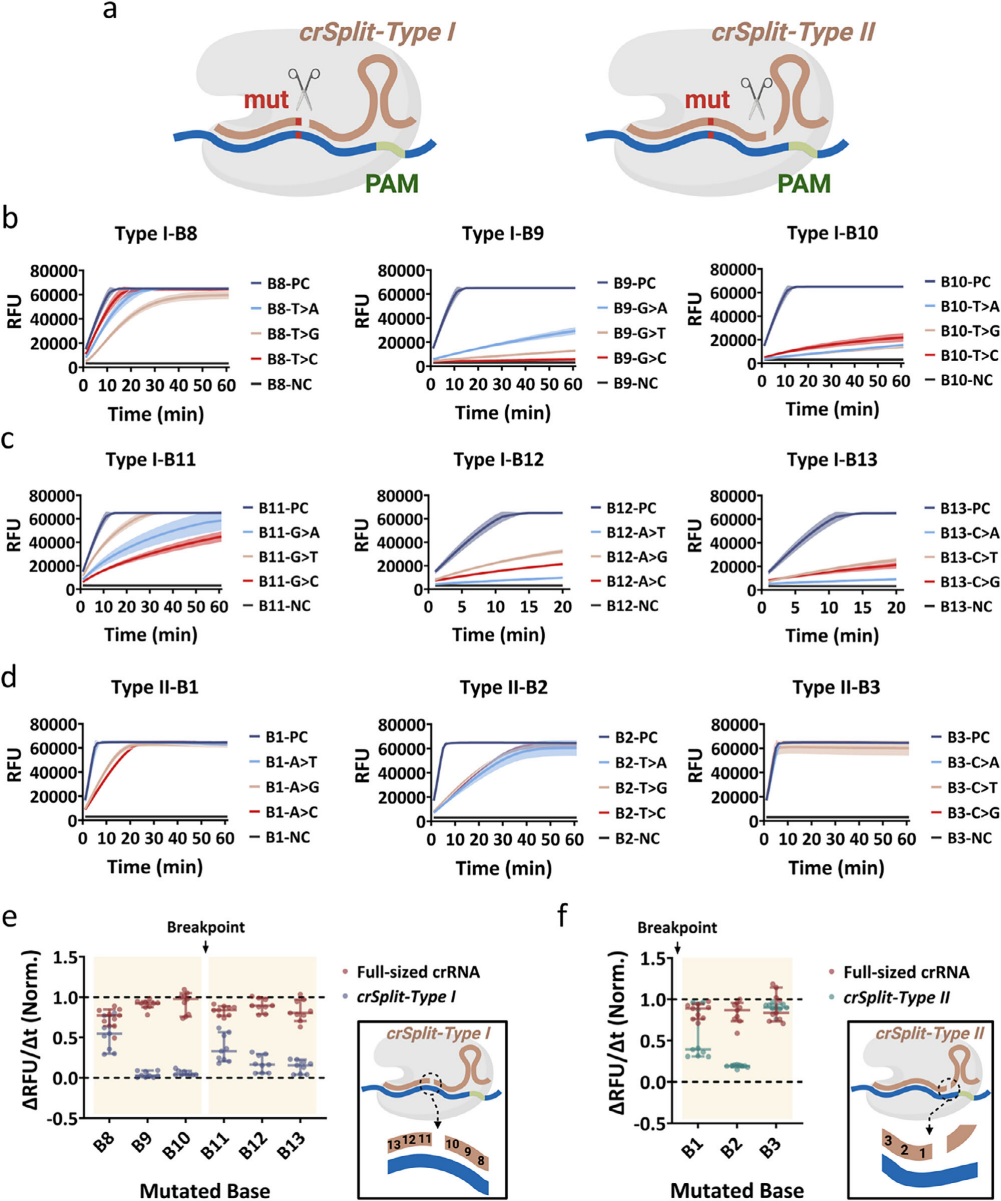
*crSplit* delivers enhanced SNV specificity. (a) Schematic of the *crSplit* strategy. (b–d) Real‐time fluorescence kinetics for SNVs at indicated positions under *crSplit* designs: (b) B8–B10 under *crSplit‐Type I*; (c) B11–B13 under *crSplit‐Type I*; (d) B1–B3 under *crSplit‐Type II*. Each plot includes the full‐sized perfect‐match control (PC), three SNV variants, and a no‐template control (NC). Data represent mean ± s.d. from three technical replicates (*n* = 3). (e,f) Comparison of normalized fluorescence growth rates (0–5 min) between full‐sized crRNA and *crSplit* designs. For each design, mutant signals were normalized to the corresponding wild‐type reference. (e) Positions B8–B13 under *crSplit‐Type I* design. (f) Positions B1–B3 under the *crSplit‐Type II* design. Each position includes three SNV types with three replicates each (*n* = 9). Data are presented as the median with 95% confidence intervals. Dashed lines mark PC (full‐sized perfect‐match control) and NC (no‐template control) reference levels. Insets illustrate the relative positions of SNVs with respect to the crRNA breakpoint in each design.

While the *actSplit* strategy markedly improves SNV discrimination, its reliance on engineered DNA activators constrains applicability in settings where artificial activators cannot be incorporated into the assay. To address this limitation, we developed a *crSplit* strategy in which an engineered breakpoint was introduced within the crRNA sequence (Figure 3a). We designed two configurations: (i) *Type I*, with the junction located within the spacer, and (ii) *Type II*, with the junction placed between the spacer and the handle. In both configurations, the crRNAs were held constant, and SNVs were systematically introduced into full‐sized single‐stranded DNA (ssDNA) activators to probe positional effects on discrimination.

Fluorescence kinetics revealed that both *crSplit* configurations attenuated nonspecific Cas12a activation in the presence of SNVs but exhibited distinct positional effects across mutation types (Figure 3b–d; Figures S7–S9). Specifically, *crSplit‐Type I* achieved stronger suppression at loci near the cleavage junction (e.g., B9, B10, B12, B13) (Figure 3b,c; Figures S7 and S9a), whereas *crSplit‐Type II* produced modest suppression at its proximal sites (e.g., B1–B2) but also reduced signals at several distal positions (e.g., B12–B13, B17–B19) (Figure 3d; Figures S8 and S9b), indicating that structural perturbations at the cleavage site can propagate along the duplex.

We next benchmarked both *crSplit* designs against the canonical full‐sized crRNA (Figure 3e,f). To quantify discrimination gains independent of mutation type, we normalized mutant signals to their wild‐type and no‐target controls and focused on sites flanking the cleavage junction (Figure 3e,f). Both split designs enhanced SNV resolution relative to the full‐sized configuration, with *Type I* showing broad suppression around the junction (Figure 3e) and *Type II* exhibiting a localized effect at B2 (Figure 3f). These results highlight that both the junction placement (*Type I* or *Type II*) and the relative positioning of the SNV shape the kinetic response landscape, demonstrating that introducing structural breaks into the crRNA enhances SNV discrimination in a position‐dependent manner compared with canonical designs.

### 2.4 Dual‐Split SDS‐CRISPR Reveals Structural Tolerance Limits

**FIGURE 4:**
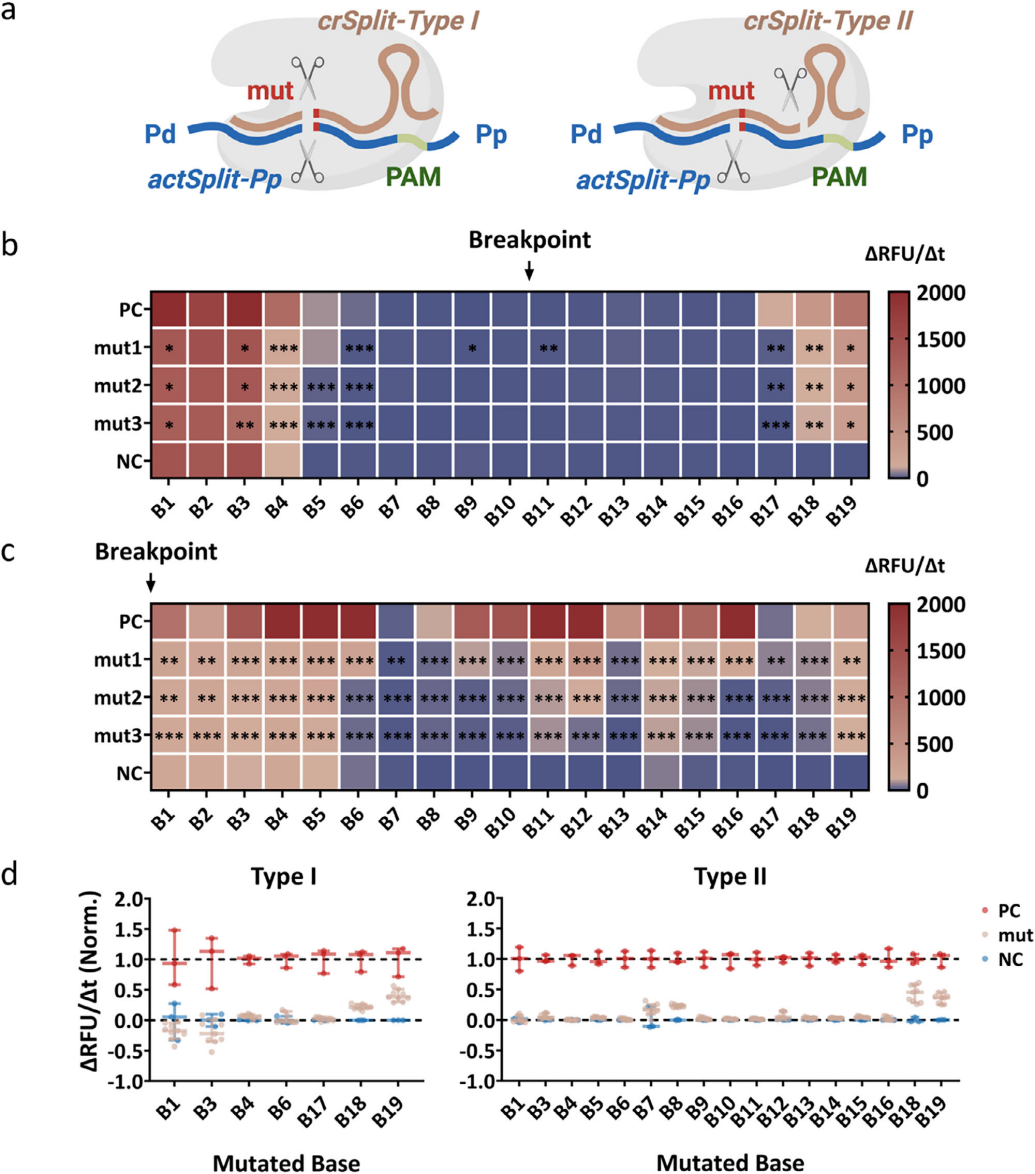
Dual‐split SDS‐CRISPR reveals structural tolerance limits. (a) Schematic of the SDS‐CRISPR system integrating both *actSplit* and *crSplit* strategies. (b) Fluorescence growth rates (0–5 min) are shown as a heatmap for each of 19 SNV positions (B1–B19) under the combined *crSplit‐Type I* and *actSplit‐Pp* design. At each position, three SNV substitutions (mut1–mut3) were evaluated and ranked by signal growth rate, alongside split activator perfect‐match control (PC) and no‐template control (NC, lacking the fragment harboring the SNV). Data represent the mean of three replicates (*n* = 3). The location of the *crSplit‐Type I* breakpoint is indicated. Statistical significance was determined relative to PC using two‐tailed unpaired t‐tests (^*^ *p* < 0.05; ^**^ *p* < 0.01; ^***^ *p* < 0.001). See Table S1 for detailed values. (c) Heatmap of fluorescence growth rates for the *crSplit‐Type II* configuration, analyzed as in (b). See Table S2 for detailed values. (d) Scatter plots showing normalized fluorescence growth rates (0–5 min) for each SNV position under the dual‐split design (*crSplit‐Type I*/*II* + *actSplit‐Pp*). For each design, mutant signals were normalized to the corresponding wild‐type reference. Positions were included if (i) pooled mut (mut1–mut3; *n* = 9) vs. PC (*n* = 3) reached *p* < 0.05 by two‐tailed exact Mann–Whitney test (with multiple‐comparison correction across positions) and (ii) PC vs. NC (*n* = 3 vs. 3) reached *p* < 0.05 by two‐tailed Welch's *t*‐test. Each dot represents an individual replicate (*n* = 9 per mut group, 3 per PC/NC group). Data are presented as the median with 95% confidence intervals. See Tables S3–S6 for detailed values.

To further evaluate the structural tolerance of the SDS‐CRISPR system, we implemented a dual‐split configuration by combining *crSplit* and *actSplit* (Figure 4a). Dual central breaks within the crRNA spacer and the activator, introduced by *crSplit‐Type I* with *actSplit*, disrupted duplex integrity and severely compromised activation at central positions, leaving only the terminal 3–5 bases detectable (Figure 4b; Figures S10 and S11). Although overall efficiency was reduced, single‐nucleotide resolution was still preserved at these terminal sites (Figure 4d), establishing a clear structural tolerance boundary in which central dual breaks severely compromise Cas12a activation. In contrast, splitting the crRNA at the handle–spacer junction (*crSplit‐Type II*) and combining this with *actSplit* avoided extensive central disruption, thereby maintaining on‐target (perfect‐match) detection while improving single‐nucleotide discrimination across many positions relative to *crSplit‐Type II* alone (Figure 4c,d; Figures S12 and S13).

Further position‐specific analysis (Figure S14) suggested that optimal split designs vary depending on SNV position. For PAM‐proximal positions (B1–B3), *actSplit‐Pp* combined with *crSplit‐Type II* likely provides superior discrimination. At central positions (B9–B11), *actSplit‐Pp*, either alone or in combination with *crSplit‐Type II*, demonstrates optimal performance, whereas combining with *crSplit‐Type I* disrupts detection. For PAM‐distal positions (B17–B19), *actSplit* alone has a limited effect; however, incorporating *crSplit (Type I or II)* moderately improves SNV detection.

To assess the generalizability of the SDS‐CRISPR platform, we evaluated both *actSplit* and *crSplit* strategies on an independent target and observed that each consistently reduced non‐specific signal (Figures S15–S18). Taken together, these findings suggest that moderate structural disruptions in either the crRNA or the activator can enhance SNV discrimination by Cas12a, whereas excessive central breaks impair activation and compromise detection.

### 2.5 SDS‐CRISPR Assay for IDH1^R132H^

In neurosurgical settings, intraoperative genotyping can guide surgical decisions and enable mutation‐directed therapy, including determining the extent of tumor resection and intraoperative drug delivery, thereby potentially improving patient outcomes [22, 23, 24, 25, 26, 27, 28]. However, conventional PCR‐based sequencing and IHC are typically incompatible with point‐of‐care timelines. In contrast, SDS‐CRISPR combines single‐base discrimination with a short turnaround time suited to intraoperative workflows, enabling rapid IDH1^R132H^ genotyping.

**FIGURE 5:**
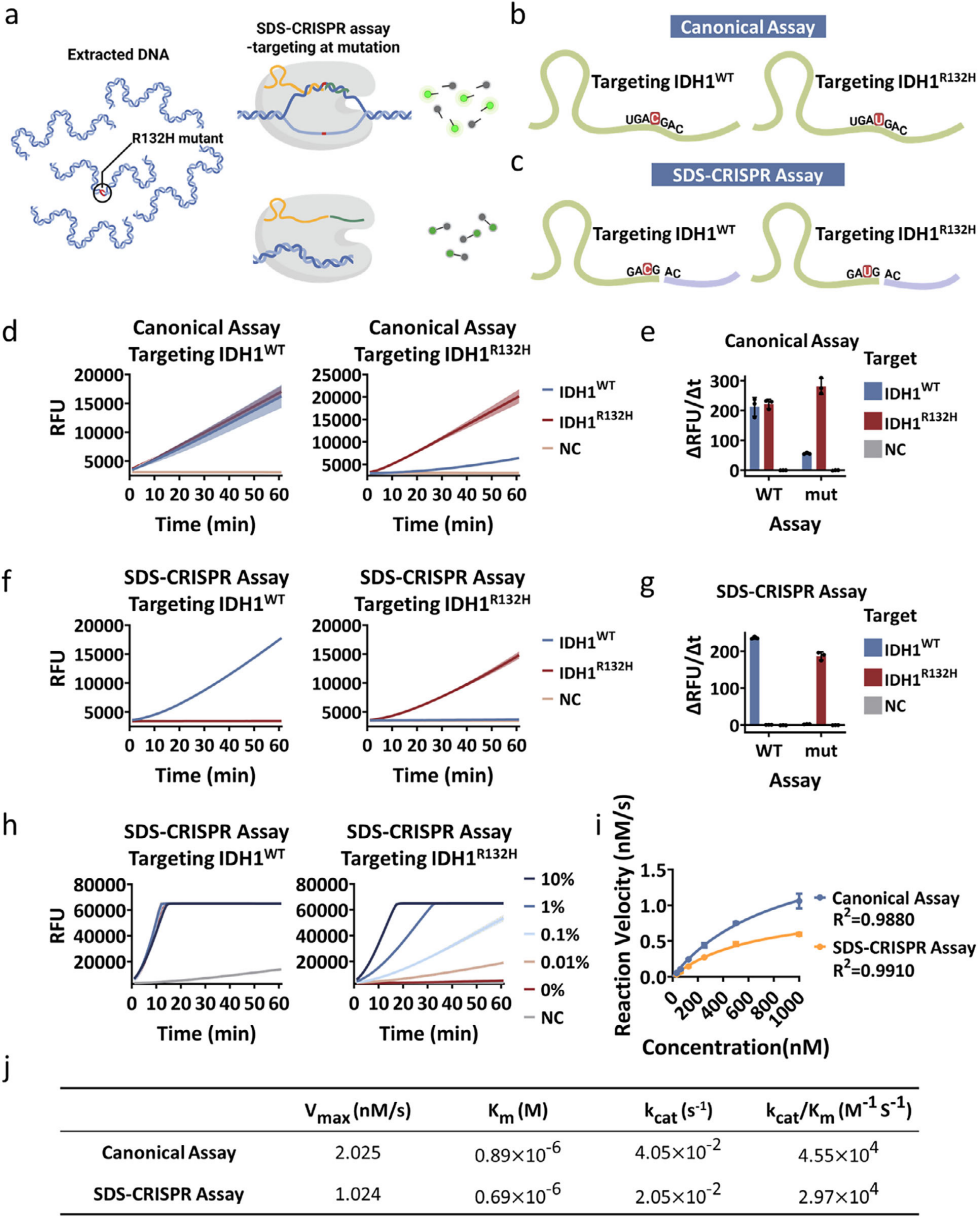
Design and performance of the SDS‐CRISPR IDH1 assays. (a) Schematic of the SDS‐CRISPR assay for IDH1^R132H^ detection, applied to extracted genomic DNA. (b,c) Comparison of canonical CRISPR assays with full‐sized crRNAs vs. (b) SDS‐CRISPR assays with the *crSplit‐Type I* design (c). (d,e) Fluorescence kinetics (d) and initial reaction rates within 5 min (e) for canonical wild‐type and mutant assays in response to IDH1^WT^ and IDH1^R132H^ targets. Data represent mean ± s.d. from three technical replicates (*n* = 3). (f,g) Fluorescence kinetics (f) and initial reaction rates within 5 min (g) for SDS‐CRISPR assays. Data represent mean ± s.d. from three technical replicates (*n* = 3). (h) Sensitivity of SDS‐CRISPR assays, tested with serial dilutions of IDH1^R132H^ DNA in a wild‐type background (10%–0.01% VAF). Data represent mean ± s.d. from three technical replicates (*n* = 3). (i) Michaelis–Menten plots showing reaction velocities of canonical and SDS‐CRISPR assays targeting IDH1^R132H^ across a range of substrate concentrations. Data represent mean ± s.d. from three technical replicates (*n* = 3). (j) Kinetic parameters derived from (i); detailed values are provided in Figure S24 and Table S7.

To establish the optimal assay architecture at this locus, we applied the crRNA‐tunable *crSplit* framework and evaluated multiple crRNA designs for IDH1^R132H^ (Figure 5a–c; Figure S19). Compared with the standard full‐sized crRNA (Figure 5d,e), *crSplit‐Type I* markedly suppressed non‐specific background while preserving detection of the matched target (Figure S19c), whereas *crSplit‐Type II* did not appreciably improve discrimination (Figure S19a,b). Prompted by reports that artificial mismatches can improve SNV recognition [31, 32, 33], we tested such modifications within the SDS‐CRISPR framework; however, these designs collapsed *trans*‐cleavage activity, even for fully matched DNA (Figure S19d,e). We next optimized variant placement and found that, under *crSplit‐Type I*, assigning the variant to the penultimate nucleotide adjacent to the split junction effectively eliminated non‐specific signal while preserving matched detection (Figure 5f,g), suggesting that this site may represent a structurally privileged position with broader utility for SNV assay design. Gel‐resolved *cis*‐cleavage paralleled the *trans* discrimination, indicating that the effect occurs at target recognition and activation rather than during reporter turnover (Figure S20).

We designed two parallel SDS‐CRISPR assays, one targeting the wild‐type and the other the mutant sequence, to enable direct comparison between alleles. By coupling SDS‐CRISPR with RPA amplification (Figure S21), the mutant‐specific assay consistently detected mutant alleles at variant allele fractions (VAFs) down to 0.01% in defined wild‐type/mutant mixtures (Figure 5h). The resulting dose–response curve (R^2^ = 0.9984) reflected quantitative sensitivity and a broad dynamic range (Figure S22). Such sensitivity supports applications beyond tumor tissue, including liquid biopsy specimens with very low mutation burdens. Consistently low background across repeated controls further demonstrated high specificity and reproducibility (Figure S23).

To assess whether the structural modifications affected enzyme turnover, we analyzed enzymatic kinetics using Michaelis–Menten modeling (Figure 5i,j; Figure S24 and Table S7). The catalytic efficiency of the SDS‐CRISPR assay targeting IDH1^R132H^ (k_cat_/K_m_ = 2.97 × 10^4^ M^−1^s^−1^) was modestly lower than that of the canonical design (k_cat_/K_m_ = 4.55 × 10^4^ M^−1^s^−1^), reflecting a trade‐off between enhanced SNV discrimination and turnover rate.

### 2.6 Exploring the Mechanism of the SDS‐CRISPR for SNV Discrimination

**FIGURE 6:**
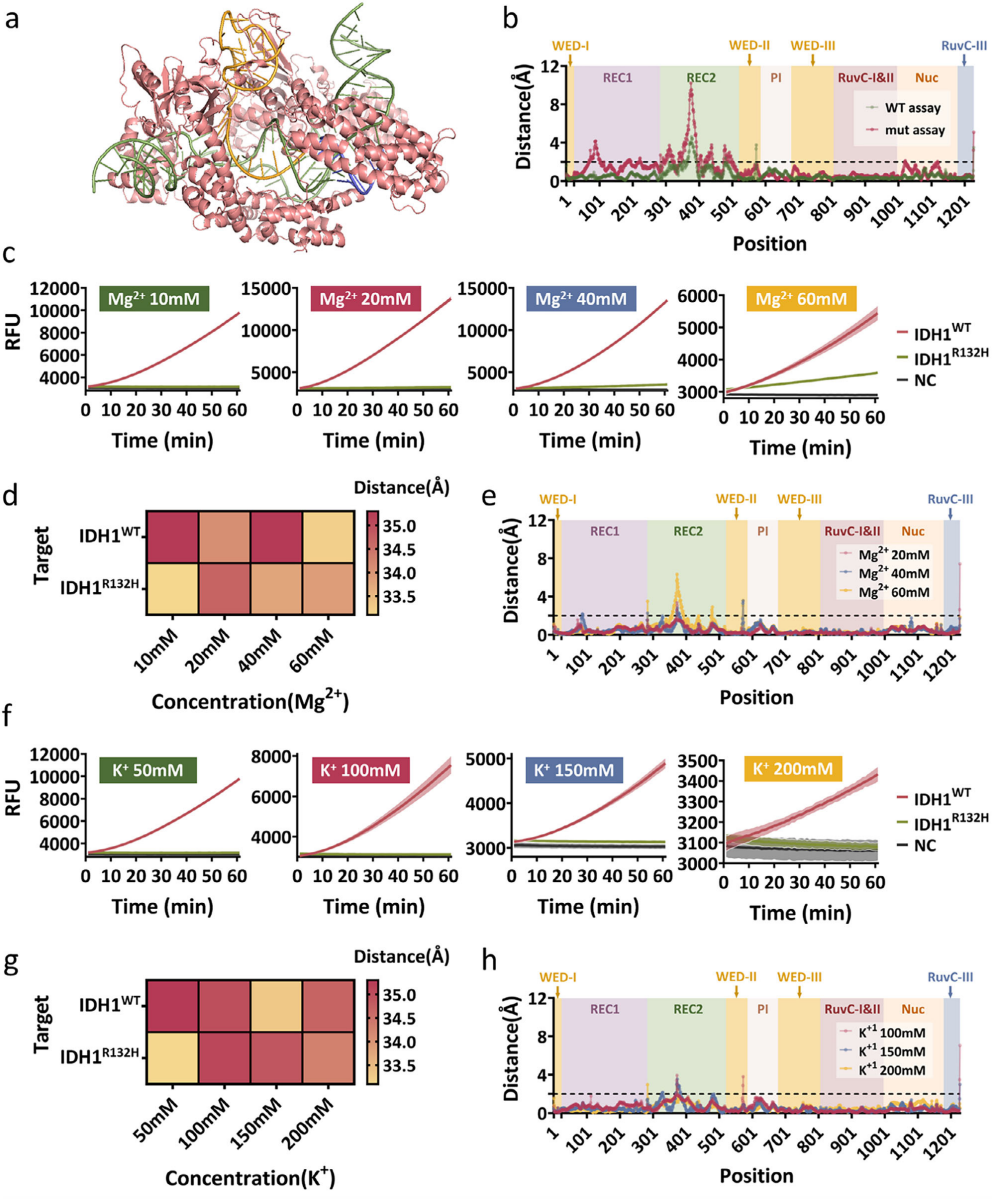
Structural responses of LbCas12a to target mismatch and varying ion concentrations. (a) Predicted 3D structure of the Cas12a–crRNA–DNA complex in the wild‐type configuration, generated using AlphaFold3 and visualized with PyMOL [42]. LbCas12a (PDB: 5XUS [36]) is shown in pink; the two segments of the crRNA are highlighted in orange and purple; and the target dsDNA is shown in green. (b) Per‐residue Euclidean distance (Å) between Cα atoms of Cas12a predicted under IDH1^WT^‐targeting and IDH1^R132H^‐targeting SDS‐CRISPR assay conditions, each bound to either wild‐type or mutant DNA. (c–e) Mg^2^^+^‐dependent structural and functional responses of Cas12a within the IDH1^WT^‐targeting SDS‐CRISPR assay. (c) Real‐time fluorescence kinetics of Cas12a *trans*‐cleavage activity under varying Mg^2^^+^ concentrations (10–60 mM), using wild‐type, mutant, or no‐target DNA templates. Data represent mean ± s.d. from three technical replicates (*n* = 3). (d) Heatmap of distance changes between Val377 and Gln1136 in Cas12a, comparing wild‐type and mutant targets across increasing Mg^2^^+^ concentrations. (e) Per‐residue Euclidean distance plots of Cas12a at 20‐, 40‐, and 60 mM Mg^2^^+^ relative to the 10 mM baseline, targeting wild‐type DNA. (f–h) K^+^‐dependent structural and functional responses of Cas12a within the IDH1^WT^‐targeting SDS‐CRISPR assay. (f) Real‐time fluorescence kinetics of Cas12a *trans*‐cleavage activity under varying K^+^ concentrations (50–200 mM), using wild‐type, mutant, or no‐target DNA templates. Data represent mean ± s.d. from three technical replicates (*n* = 3). (g) Heatmap of distance changes between Val377 and Gln1136 in Cas12a, comparing wild‐type and mutant targets across increasing K^+^ concentrations. (h) Per‐residue Euclidean distance plots of Cas12a at 100‐, 150‐, and 200 mM K^+^ relative to the 50 mM baseline, targeting wild‐type DNA.

To further probe the mechanistic basis of the discrimination–turnover trade‐off, we modeled the IDH1^WT^‐specific SDS‐CRISPR assay using AlphaFold3 [34, 35], comparing conformations bound to either IDH1^WT^ or IDH1^R132H^ targets (Figure 6a). In parallel, we modeled the canonical CRISPR assay in target‐bound (active) and target‐free (inactive) states as structural references. At the global level, root mean square deviation (RMSD) analysis of canonical active and inactive states revealed a large conformational shift (3.1 Å), consistent with target‐dependent switching^35^. In contrast, SDS‐CRISPR assays exhibited only minor shifts between matched IDH1^WT^ and mismatched IDH1^R132H^ complexes (0.7 Å for the IDH1^WT^‐specific design and 0.5 Å for the IDH1^R132H^‐specific design), even though *trans*‐cleavage CRISPR assays revealed clear fluorescence discrimination, indicating that SDS‐CRISPR relies on an alternative structural mode for target recognition. To refine this view, we analyzed the Val377–Gln1136 distance (Figure S25), an activation‐linked metric capturing REC2–NUC rearrangements and previously proposed as a structural readout of Cas12a activation [36, 37]. In canonical assays, this metric shifted from 23.1 Å (inactive) to 33.2 Å (active), whereas in SDS‐CRISPR assays it expanded to approximately 35.3 Å with matched wild‐type targets but contracted to 32.3–33.1 Å with mismatched mutant targets (Table S8), indicating that subtle conformational shifts correlate with SNV discrimination. Per‐residue distance mapping further pinpointed REC2 as the dominant site of structural divergence, with supplementary shifts in REC1, WED‐II, and RuvC‐III (Figure 6b), establishing REC2‐centered modulation as a key determinant of SDS‐CRISPR specificity.

To investigate how the ionic environment modulates SDS‐CRISPR activity, we performed systematic Mg^2^^+^ titrations using the IDH1^WT^‐targeting SDS‐CRISPR assay. For wild‐type targets, increasing Mg^2^^+^ from 10 to 60 mM progressively suppressed fluorescence output (Figure 6c). This effect was mirrored at the structural level: Val377–Gln1136 distance analysis revealed a shift from the fully active state at 10–40 mM toward partially activated conformations at 60 mM (Figure 6d), while residue‐level mapping showed pronounced conformational rearrangements in the REC2 domain at high Mg^2^^+^ (Figure 6e). In contrast, for mutant targets, higher Mg^2^^+^ concentrations (40–60 mM) produced elevated non‐specific activation (Figure 6c). Consistently, Val377–Gln1136 measurements indicated unexpected activation under excess Mg^2^^+^ (Figure 6d), and residue‐level mapping revealed additional fluctuations in the NUC domain (Figure 6e; Figure S26a). Comparative analyses of matched and mismatched complexes further confirmed this trend: structural differences evident at 10 mM gradually diminished with increasing Mg^2^^+^, and by 60 mM REC2 showed almost no detectable divergence (Figure S27a). Together, these results demonstrate that excess Mg^2^^+^ undermines SNV selectivity by destabilizing REC2‐mediated activation in matched complexes while inducing spurious NUC‐domain fluctuations in mismatched ones.

We next assessed whether K^+^ concentration influences SNV discrimination in the IDH1^WT^‐targeting SDS‐CRISPR assay. Unlike Mg^2^^+^, increasing K^+^ from 50 to 200 mM modulated fluorescence intensity for wild‐type targets without elevating non‐specific signals (Figure 6f). Structural analyses paralleled these findings: at 50 mM K^+^, the Val377–Gln1136 distance most clearly distinguished wild‐type from mutant complexes (Figure 6g). Wild‐type complexes showed minimal structural variation (Figure 6h), whereas mutant complexes displayed modest conformational shifts (Figure S26b). Crucially, across all K^+^ concentrations, wild‐type and mutant complexes remained consistently distinguishable (Figure S27b), underscoring the robustness of K^+^‐supported SNV selectivity.

Collectively, AlphaFold3‐based modeling revealed that SDS‐CRISPR responds to both SNVs and ionic perturbations through distinct conformational adjustments, with REC2 emerging as the most consistent site of divergence. Previous studies have implicated REC2 in allosteric communication with the NUC domain and in regulating DNA breathing during R‐loop formation [38, 39, 40]. SNVs, in turn, have been reported to stall R‐loop propagation and trap Cas12a in intermediate states [41]. Integrating these insights, our findings establish REC2‐centered modulation as a mechanistic determinant of SNV discrimination in the SDS‐CRISPR system.

### 2.7 AI‐Powered SDS‐CRISPR for Clinical Detection of IDH1^R132H^

**FIGURE 7:**
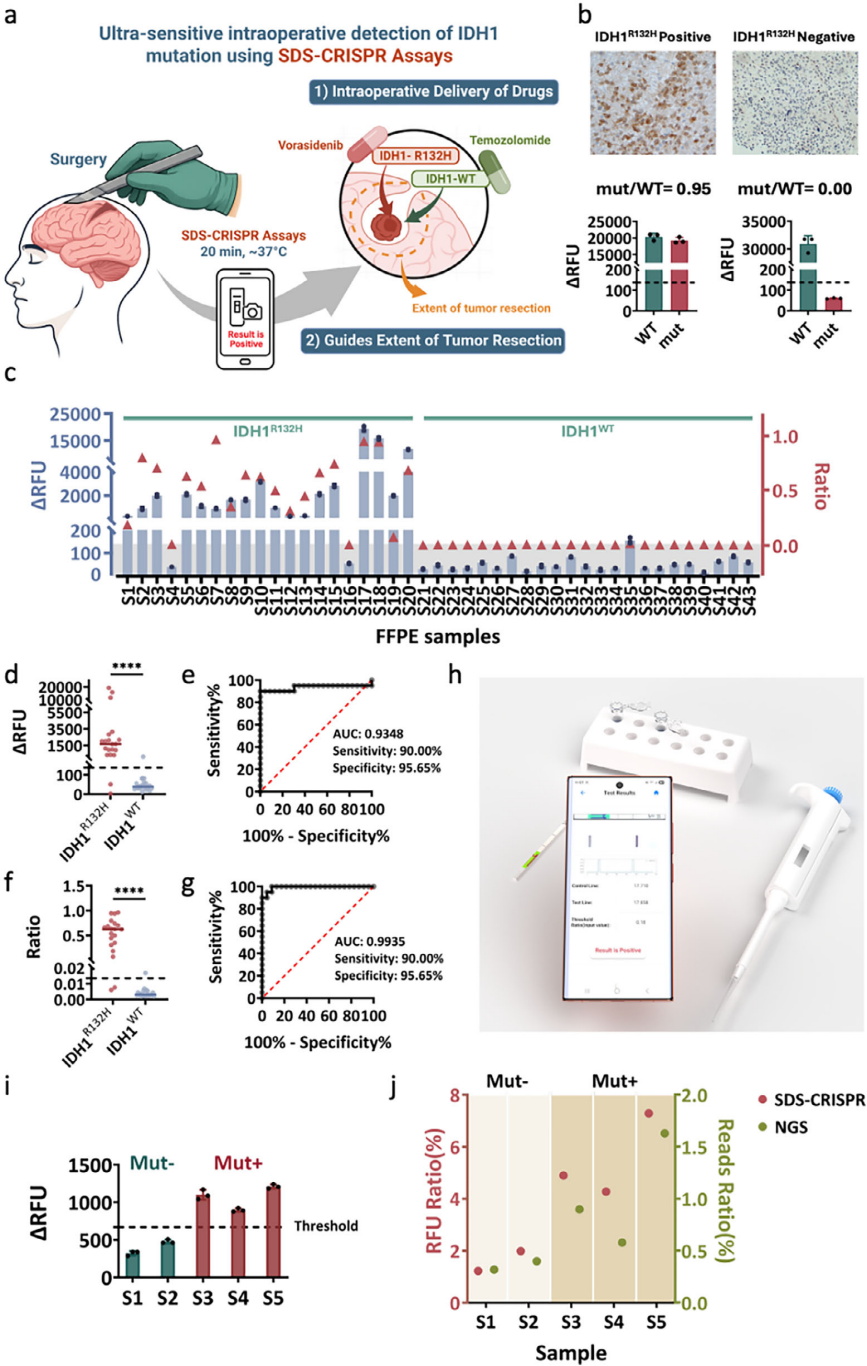
Clinical detection of IDH1^R132H^ mutations using the SDS‐CRISPR assays. (a) Schematic workflow illustrating clinical use of the SDS‐CRISPR assay for ultra‐sensitive intraoperative detection of IDH1^R132H^ mutations, supporting both real‐time therapeutic decision‐making and guidance of tumor resection extent. (b) Representative clinical samples (S17, S41) validated by IHC (top, IDH1^R132H^‐positive and ‐negative) and corresponding fluorescence readouts from SDS‐CRISPR assays (bottom). Data represent mean ± s.d. from three technical replicates (*n* = 3). Threshold values were calculated as mean + 3 × s.d. of negative samples. (c) Blue bars represent the fluorescence increase within the first 5 min, plotted as mean ± s.d. from three replicates and aligned to the left *y*‐axis. Red triangles show the R132H/WT signal ratio, referenced to the right *y*‐axis. Cutoff thresholds for both metrics were aligned and defined as the mean + 3 × s.d. of negative controls. The gray shaded region indicates values below the cutoff. (d–g) Quantitative analysis of the same FFPE cohort. (d) Distribution of ΔRFU values with statistical comparison by two‐tailed Mann–Whitney *U* test (^****^ *p* < 0.0001). (e) ROC curve of ΔRFU, yielding an AUC of 0.9348 with 90.0% sensitivity and 95.65% specificity. (f) Distribution of mutant‐to‐wild‐type ratios with two‐tailed Mann–Whitney *U* test ^(****^ *p* < 0.0001). (g) ROC curve of the ratio, yielding an AUC of 0.9935 with 90.0% sensitivity and 95.65% specificity. (h) Workflow and smartphone app (AI‐powered) analysis for the lateral‐flow strip readout. (i) Detection of plasma cfDNA from IDH1‐confirmed samples using SDS‐CRISPR; mutant‐positive (red) and negative (green) samples were classified by ΔRFU. Threshold values were calculated as mean + 3 × s.d. of negative samples. (j) Correlation between mutation fractions quantified by SDS‐CRISPR signal ratios and targeted NGS in matched plasma cfDNA samples.

Given the need for rapid IDH1^R132H^ identification to guide intraoperative decisions and interventions, we translated the SDS‐CRISPR assays into a portable diagnostic system that seamlessly integrates lateral‐flow readout with smartphone‐based AI interpretation. The assay operates isothermally at 37°C and delivers results within 20 min, aligning with surgical timelines. Lateral‐flow strips convert molecular activity into binary visual signals, while a dedicated smartphone application standardizes image acquisition, applies AI‐based background correction, and thresholds line intensities for robust interpretation. This integration transforms the assay from a benchtop method into a workflow‐compatible diagnostic tool, enabling rapid, real‐time mutation calls to support decisions on resection margins and mutation‐directed therapy within the operative window (Figure 7a).

First, we conducted clinical validation using formalin‐fixed paraffin‐embedded (FFPE) glioma specimens through a conventional fluorescence detection approach. To mitigate specimen‐to‐specimen variability in cellularity, we used a dual‐readout strategy: absolute ΔRFU from the IDH1^R132H^‐targeting SDS‐CRISPR assay and the R132H/wild‐type signal ratio. Among 43 IHC‐confirmed cases (20 IDH1^R132H^‐positive, 23 wild‐type; Figure 7b,c; Figures S28–S31 and Table S9), both readouts separated mutant from wild‐type samples (two‐tailed Mann–Whitney *U* test, ^****^ *p* < 0.0001; Figure 7d,f). Receiver operating characteristic (ROC) analysis yielded an area under the curve (AUC) of 0.9348 for ΔRFU and 0.9935 for the ratio (Figure 7e,g); at the selected operating point, sensitivity reached 90.0% and specificity 95.65%. The ratio outperformed ΔRFU, consistent with normalization to the wild‐type control reducing cellularity‐driven variability.

To evaluate assay sensitivity in a clinically relevant setting, we performed serial dilutions of FFPE‐derived glioma specimens and assessed detection limits using both ΔRFU and ratio readouts. Thresholds were defined as the wild‐type mean plus three standard deviations (s.d.). ΔRFU reliably detected down to a 1:100 dilution (cycle threshold [Ct] = 37.43), whereas the ratio readout extended this limit to 1:1,000 (Ct = 39.28) (Figure S32; Tables S10 and S11). This level of sensitivity underscores the robustness of SDS‐CRISPR for samples with limited cellular material, a frequent constraint in intraoperative and archival workflows.

After validation, we adapted the assay for intraoperative use by converting the fluorescence readout into a lateral‐flow format, enabling rapid interpretation at the bedside without specialized equipment (Figure 7h; Figures S33 and S34). The companion smartphone application standardizes strip evaluation through automated line quantification and AI‐based background correction (Figure S35), ensuring objective, reproducible results across users and operating room conditions. Applied to FFPE glioma specimens, strip‐based readouts yielded classifications fully concordant with fluorescence assays (Figures S36–S41), supporting translation to intraoperative testing.

We next assessed SDS‐CRISPR performance on plasma circulating‐free DNA (cfDNA), a minimally invasive substrate for molecular profiling. We tested five plasma samples: three from patients with tumor‐confirmed IDH1^R132H^ mutations by NGS and two wild‐type controls (Table S9). SDS‐CRISPR assays clearly distinguished mutant‐positive from wild‐type samples based on fluorescence signals (Figure 7i). For comparison, we performed targeted amplicon sequencing (∼50 000 paired‐end reads per sample) and calculated R132H/wild‐type read ratios (Table S12). These ratios showed strong concordance with SDS‐CRISPR fluorescence ratios (Figure 7j; Table S13), demonstrating that SDS‐CRISPR provides a rapid, sequencing‐consistent alternative for plasma‐based mutation detection and enables minimally invasive molecular monitoring.

### 2.8 Broad Applicability of SDS‐CRISPR

To demonstrate the versatility of SDS‐CRISPR across nucleic acid types, we applied it to two clinically relevant contexts, miRNA family discrimination and antiviral drug‐resistance mutation detection, both of which involve highly homologous sequences that challenge conventional assays.

**FIGURE 8:**
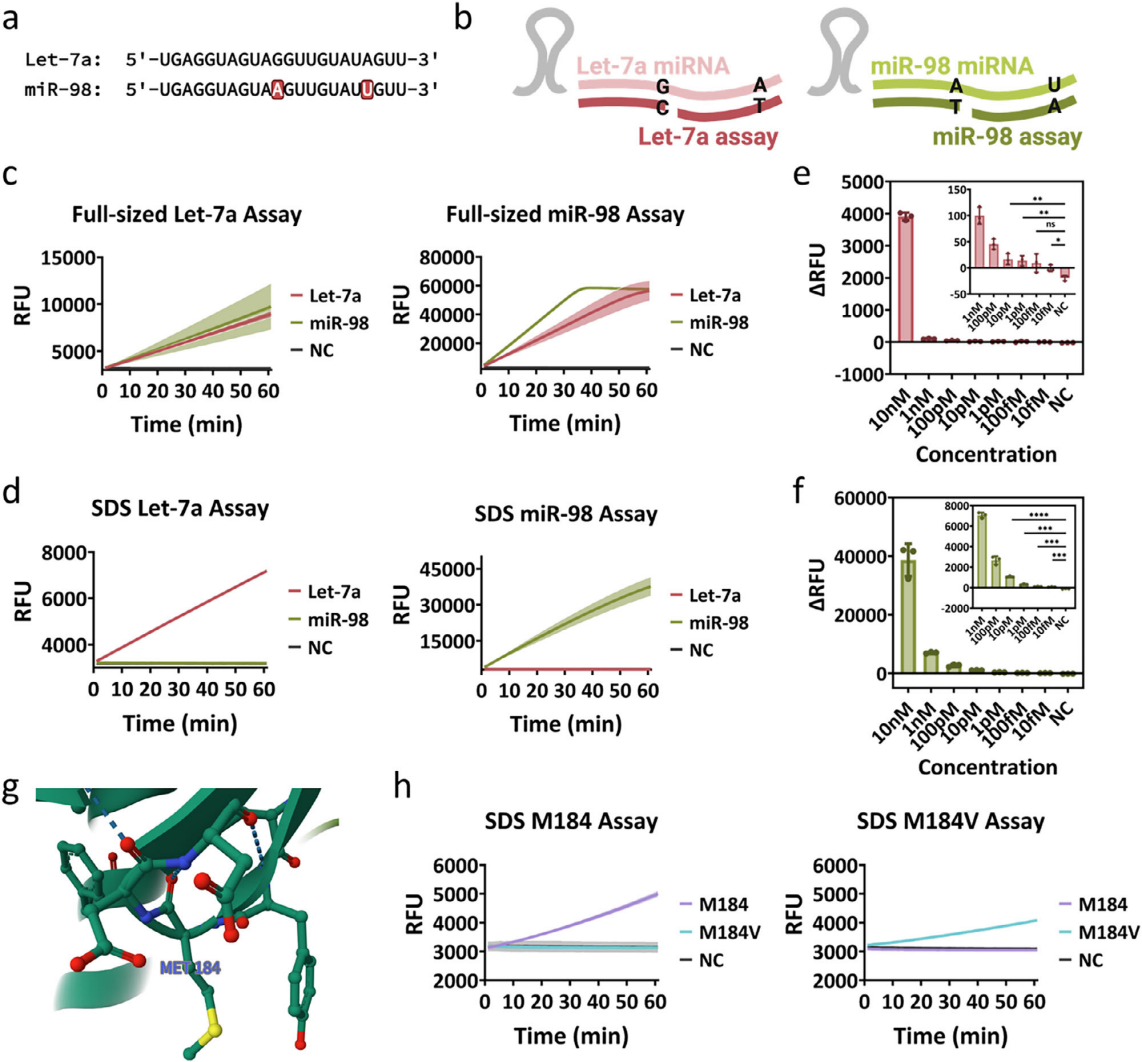
SDS‐CRISPR enables discrimination of closely related miRNAs and clinically relevant drug‐resistance mutations. (a) Sequence alignment of Let‐7a and miR‐98 miRNAs, highlighting sequence mismatches. (b) Schematic of SDS‐CRISPR assays using the *actSplit* strategy for miRNA discrimination, in which the target miRNA functions as the crRNA spacer. (c,d) Real‐time fluorescence kinetics of canonical CRISPR assays (c) and SDS‐CRISPR assays (d) targeting Let‐7a and miR‐98. (e,f) Sensitivity of SDS‐CRISPR assays for Let‐7a (e) and miR‐98 (f) across target concentrations from 10 nM to 10 fM. Fluorescence signals increased within 60 min of reaction. Statistical significance was assessed by two‐tailed unpaired *t*‐tests ^(*^ *p* < 0.05; ^**^ *p* < 0.01; ^***^ *p* < 0.001; ^****^ *p* < 0.0001). Exact P values are provided in the Tables S14 and S15. (g) Structural model of HIV reverse transcriptase showing the M184 site (PDB: 1HMV [46]). (h) SDS‐CRISPR assays for wild‐type M184 and mutant M184V showing selective detection of each variant.

The Let‐7 family represents a prototypical group of tumor‐suppressor miRNAs whose members differ by as little as one or two nucleotides [43], posing a stringent challenge for sequence discrimination. Within this family, Let‐7a and miR‐98 differ only at positions 11 and 19 (Figure 8a). Given their intrinsic short length, miRNAs are well suited to serve as spacers in the *crSplit‐Type II* design, and pairing them with engineered *actSplit* activators enables SDS‐CRISPR to fully leverage its design advantages. We therefore designed both canonical full‐sized and SDS‐CRISPR activators targeting Let‐7a and miR‐98 (Figure 8b) and validated the assays using synthetic miRNAs. Whereas the canonical design showed limited discrimination (Figure 8c), the SDS‐CRISPR assays achieved markedly improved specificity while maintaining robust signal output (Figure 8d). To assess clinical potential, we performed concentration‐gradient experiments for both canonical and SDS‐CRISPR assays. Both platforms detected inputs down to 10 fM within 60 min, but only the SDS‐CRISPR assay combined comparable sensitivity with substantially enhanced specificity (Figure 8e,f; Figure S42).

To extend SDS‐CRISPR to RNA mutation detection, we focused on the clinically important M184V substitution in HIV reverse transcriptase (Figure 8g), a single‐nucleotide change that confers resistance to the nucleoside analogs lamivudine (3TC) and emtricitabine (FTC) [44]. Because routine workflows analyze viral RNA mutations via reverse‐transcribed cDNA [45], they provide a natural substrate for SDS‐CRISPR. Guided by the IDH1^R132H^ design principle, placing the variant at the penultimate nucleotide adjacent to the *crSplit‐Type I* junction, we developed assays for both M184 and M184V. Using synthetic wild‐type and mutant templates, these assays achieved high specificity and unambiguous single‐base discrimination (Figure 8h), underscoring the penultimate‐adjacent position as a generalizable design hotspot and highlighting the utility of SDS‐CRISPR for resistance monitoring in RNA viruses.

In summary, these results establish SDS‐CRISPR as a versatile platform capable of resolving highly homologous nucleic acid sequences across both DNA and RNA contexts. By enabling precise discrimination of closely related miRNA isoforms and clinically relevant RNA SNVs such as HIV drug‐resistance mutations, SDS‐CRISPR demonstrates broad applicability to diverse molecular targets. These attributes underscore its translational potential for point‐of‐care diagnostics in oncology, infectious disease, and beyond.

## 3 Discussion

Simple, rapid, and reliable discrimination of SNVs remains a major challenge in molecular diagnostics, as existing approaches are often constrained by sequence context and require extensive site‐specific optimization, limiting their scalability and translational potential. SDS‐CRISPR provides a structurally programmable solution by embedding engineered structural perturbations into the Cas12a–nucleic acid complex, thereby decoupling assay performance from sequence dependence and establishing a generalizable framework for SNV detection. The platform offers three principal advantages: (i) its engineered structural breaks confer robust specificity, preserving clear mutant–wild‐type separation in interference‐rich clinical matrices, including FFPE tissue and plasma cfDNA; (ii) it enables rapid, point‐of‐care testing, delivering results within 20 min without complex instrumentation via lateral‐flow readout and AI‐assisted smartphone analysis; and (iii) the design generalizes across analytes, spanning DNA variants, short RNAs (e.g., miRNA isoforms), and RNA SNVs, supporting applications from tumor genotyping to antiviral resistance monitoring. Together, these attributes establish SDS‐CRISPR as a universal, structurally programmable, and clinically actionable platform for precision diagnostics.

SDS‐CRISPR introduces structure‐disruptive nicks into the DNA activator (*actSplit*) or the crRNA (*crSplit*) to suppress inter‐allelic cross‐activation while maintaining turnover, yielding high signal‐to‐background discrimination across both DNA and RNA targets. As independent design levers, *actSplit* provides the largest gains when the junction lies near the variant, strongest at mid‐duplex positions, with only modest wild‐type signal penalty, whereas *crSplit* shows distinct positional behavior: *Type I* acts locally around the break, while *Type II* propagates attenuation to distal loci. A tolerance map constructed from both levers reveals that dual central breaks largely extinguish Cas12a activation, leaving only the terminal 3–5 nt resolvable. By contrast, *actSplit* combined with *crSplit‐Type II* avoids central over‐disruption, preserves on‐target detection, and improves single‐base resolution across multiple positions. This pattern reproduces in an independent target, indicating a Cas12a‐specific response; in practice, moderate, well‐placed breaks enhance specificity, whereas excessive central disruption impairs catalytic switching. As with other Cas12a‐based detection systems, the applicability of SDS‐CRISPR is influenced by PAM availability and local protospacer context; however, the known tolerance of Cas12a to suboptimal or non‐canonical PAMs [36, 47, 48, 49, 50] broadens the range of targetable mutation sites in practice.

Mechanistically, AlphaFold3 models suggest a REC2‐centered rearrangement as a plausible basis for discrimination. In SDS‐CRISPR, allele calling appears to arise from subtle, target‐dependent conformational shifts rather than the large inactive‐to‐active toggle characteristic of canonical assays. Two model‐derived metrics, the Val377–Gln1136 separation and residue‐wise deviations concentrated in REC2 (with smaller contributions from REC1, WED‐II, and RuvC‐III), covary with fluorescence output, supporting a REC2‐focused working model. Because Cas12a activation involves coordinated rearrangements across multiple structural elements, changes in a single residue‐to‐residue distance may not necessarily vary monotonically with ionic conditions or directly mirror fluorescence output. In this context, the broader residue‐wise analyses provide a more comprehensive structural view of the conformational responses underlying SDS‐CRISPR activity. We note that this represents a correlative structure–function link; causal validation will require perturbational and structural studies (e.g., REC2 mutagenesis, FRET distance reporters, time‐resolved cryogenic electron microscopy, or molecular dynamics). Because Mg^2^^+^ and K^+^ are the principal ions in CRISPR buffers, with Mg^2^^+^ supporting catalysis and duplex stability and K^+^ modulating ionic strength and conformational balance [51, 52, 53], we quantified their impact on selectivity. Operationally, SDS‐CRISPR performs best at 10–40 mM Mg^2+^ with 50–150 mM K^+^, while avoiding Mg^2^^+^ ≥ 60 mM, which compresses mutant–wild‐type contrast and elevates background.

Compared with a qPCR design leveraging 3’‐end primer discrimination [54, 55] (qPCR‐pair2, Table S20), which produced off‐target amplification and non‐specific signal, the SDS‐CRISPR assays eliminated background while maintaining high sensitivity. This advantage stems from its structural‐disruption strategy: by introducing instability into the crRNA–DNA activator duplex, SDS‐CRISPR converts the fragility of mismatched hybrids into a selective filter resilient to the complexity of clinical DNA. This principle underlies the clean separation of wild‐type and mutant signals and explains the assay's robustness in challenging contexts such as the extensively fragmented and chemically crosslinked DNA derived from FFPE clinical samples and low‐input cfDNA.

In the FFPE cohort, SDS‐CRISPR achieved high sensitivity and specificity, with two false positives and one false negative, likely reflecting tissue heterogeneity and DNA quality. Serial dilutions of FFPE‐derived specimens, benchmarked against qPCR using synthetic standards, confirmed reliable detection down to Ct = 37.43 (1:100) by ΔRFU and Ct = 39.28 (1:1000) by ratio, corresponding to sub‐femtomolar concentrations (≤  100 aM) (Table S18). These results demonstrate that SDS‐CRISPR maintains performance even when tissue input is extremely limited, a frequent constraint in intraoperative and archival workflows.

Preliminary evaluation in fresh‐frozen negative samples showed similarly low background (Figure S43 and Table S19), supporting the feasibility for fresh tissue analysis. While the limited number of IDH1^R132H^‐positive plasma samples reflects the suboptimality of plasma‐based glioma mutation detection [56, 57], future work will extend this framework to cerebrospinal fluid monitoring [57, 58, 59] and to other disease contexts such as acute leukemia [60, 61]. The dual‐metric design buffers against template variability, and the combination of lateral‐flow readout with AI‐based analysis minimizes operator subjectivity, meeting intraoperative demands for speed and simplicity. To further improve ratio quantification and streamline testing, we plan to integrate mutant and wild‐type assays onto a single lateral‐flow strip and upgrade the smartphone application for simultaneous dual‐readout analysis.

SDS‐CRISPR is not limited to DNA mutations in glioma; the same framework directly interrogates SNVs on RNA. With *crSplit‐Type II*, the intrinsic short length of miRNAs makes them natural spacers, enabling isoform‐level discrimination of closely related Let‐7 family members while preserving 10 fM sensitivity within 60 min. Beyond sensitivity, we used the miRNA assay to examine ionic dependence under the *actSplit* design. Elevated K^+^ concentrations (> 150 mM) attenuated signal intensity, whereas increasing Mg^2^^+^ up to 60 mM did not raise background (Figure S44), indicating that SDS‐CRISPR may operate under distinct modes of ionic regulation between *actSplit* and *crSplit* configurations. Extending the principle from native RNA to its cDNA intermediates, *crSplit‐Type I* unambiguously resolved the HIV‐1 M184V resistance mutation. These demonstrations reveal that structural disruption is not a niche modification but a transferable rule that operates across nucleic‐acid classes and clinical contexts. In *crSplit‐Type I* assays designed for detecting intact exogenous DNA or amplification products, the recurrent success of positioning variants at the penultimate base near the split provides additional evidence for a broadly exploitable design hotspot, which we plan to validate across diverse SNVs to establish its universality. For *crSplit‐Type II* configurations, typically coupled with the *actSplit* strategy for RNA targets such as miRNAs, placing the SNV at PAM‐proximal, central, or PAM‐distal regions consistently improves mutation discrimination while largely preserving matched wild‐type activation.

In this sense, SDS‐CRISPR defines a new class of structurally engineered diagnostics, programmable in design, rooted in mechanism, and readily portable. Instead of building isolated assays for each target, the platform applies a single guiding principle: use rational disruption to convert molecular specificity into robust clinical readouts. Its reach, from tumor genotyping to viral drug resistance and miRNA isoform discrimination, underscores SDS‐CRISPR as a versatile foundation for next‐generation precision diagnostics.

## 4 Methods

### 4.1 Ethical Statement

Deidentified clinical samples, including tissue and plasma samples, were collected at the University of Connecticut Health Center under approval and guidelines from the ethics committee of UConn Health (IRB# 08‐310‐1 and GP1686).

### 4.2 Materials

Nuclease‐free water was purchased from New England Biolabs (NEB). The TwistAmp Basic kit was purchased from TwistDx Limited. All nucleotides were synthesized by Integrated DNA Technologies (IDT). Alt‐R L.b. Cas12a (Cpf1) Ultra was obtained from IDT. NEBuffer 4 was obtained from NEB. For gel electrophoresis, 40% Acrylamide/Bis Solution (19:1), Ammonium Persulfate, 10 × TBE buffer, and TEMED were purchased from Bio‐Rad Laboratories. SYBR Gold Nucleic Acid Gel Stain (10 000 × Concentrate in DMSO) was purchased from Thermo Fisher Scientific. The MinElute PCR Purification Kit, DNeasy Blood and Tissue Kit, QIAamp Circulating Nucleic Acid Kit, and QIAamp DNA FFPE Tissue Kit were obtained from QIAGEN. The GoTaq Probe qPCR kit was purchased from Promega. Magnesium chloride was purchased from Sigma–Aldrich (Germany). Potassium chloride was purchased from Thermo Fisher Scientific. HybriDetect strips were purchased from Milenia Biotec.

### 4.3 CRISPR‐Cas12a *trans*‐Cleavage Assay

For SDS‐CRISPR assays, 10 µL reaction mixtures were prepared containing 1× NEBuffer 4, 200 nM crRNA (either full‐sized or split components), 100 nM Alt‐R Cas12a, 5 µM reporter, and 1 µL of 1 µM ssDNA activator (either full‐sized or split components). After thorough mixing, reactions were incubated at 37°C in a CFX96 Touch Real‐Time PCR Detection System for 60 min, with fluorescence measurements recorded every minute.

### 4.4 RPA Assay

RPA reactions were conducted using the TwistAmp Basic kit, adhering to the instructions provided in its manual. The reaction mixture comprised 29.5 µL of rehydration buffer, 480 nM forward and reverse primers, 14 mM magnesium acetate, and 7.5 µL of either plasmid or extracted DNA. For the negative control, nuclease‐free water was used instead of nucleic acid. Subsequently, the reaction proceeded at 39°C for 15 min.

### 4.5 Native PAGE

To prepare the gel, 4 mL of 40% Acrylamide/Bis Solution (19:1), 2 mL of 10×TBE buffer, 14 mL of deionized water, 8 µL of TEMED, and 66 µL of 30% APS solution (prepared using 3 mg in 100 µL nuclease‐free water) were mixed. The mixture was then transferred to the gel‐casting chamber and allowed to solidify. All samples were premixed with gel loading dye and added to each lane of the gel. Electrophoresis was conducted at a constant 100 V for 90 min in 1 × TBE buffer. Subsequently, the gel was stained with dissolved SYBR Gold Nucleic Acid Gel Stain solution for 5 min in a dark environment. Images were obtained using the Bio‐Rad ChemiDoc MP imaging system.

### 4.6 SDS‐CRISPR Assays for IDH1 Mutation Detection

The SDS‐CRISPR *trans*‐cleavage reaction was performed in a final volume of 10 µL containing 1 × NEBuffer 4, 200 nM crRNA (full‐sized or split components), 100 nM Alt‐R Cas12a, 5 µM reporter, and 1 µL of 10 nM synthetic double‐stranded DNA (dsDNA) or RPA amplicons. Reactions were incubated at 37°C in a CFX96 Touch Real‐Time PCR Detection System for 60 min, with fluorescence recorded every minute.

### 4.7 Michaelis–Menten Kinetics Calculations

For kinetic measurements [62, 63], reactions were set up in a total volume of 20 µL with 1 × NEBuffer 4, 25 nM crRNA (either full‐sized or split components), 50 nM Alt‐R Cas12a, and 1 µL of 10 nM synthetic IDH1^WT^ dsDNA or water. After incubation at 37°C for 20 min, varying concentrations of ssDNA reporter (31.25 nM, 62.5 nM, 125 nM, 250 nM, 500 nM, and 1 µM) were added, and the reaction was further incubated at 37°C. Fluorescence was recorded every 30 s, with the first 300 s of data used to calculate initial velocities.

For fluorescence calibration, reporter solutions covering the same concentration range were prepared and fully digested in the presence of 25 nM crRNA (either full‐sized or split components), 50 nM Alt‐R Cas12a, 1 × NEBuffer 4, and 1 µL of 10 nM synthetic IDH1^WT^ dsDNA or water at 37°C for 60 min. Endpoint fluorescence values obtained after complete digestion were used to establish the relationship between fluorescence intensity and cleaved reporter concentration.

For the canonical assay, a linear relationship between fluorescence and cleaved reporter concentration (nM) was established (*F_c_* = 24.47 *C_c_*), where *F_c_* represents the fluorescence signal (a.u.) from the fully cleaved reporter, and *C_c_* is the concentration of cleaved reporter (nM). Calibration for the uncleaved reporter was similarly performed (*F_u_* = 0.1610 *C_u_*), with *F_u_* indicating the fluorescence from the uncleaved reporter and *C_u_* its concentration (nM) (Figure S24a–c). For the SDS‐CRISPR assay, fluorescence calibration yielded *F_c_* = 23.19 *C_c_* for cleaved reporter and *F_u_* = 0.1990 *C_u_* for uncleaved reporter (Figure S24d–f).

The total fluorescence at each time point was modeled as the sum of contributions from cleaved and uncleaved reporters:

(1) $$F{\left(t\right)}_{canonical}=24.47\;C_{c}\left(t\right)+0.1610\;C_{u}\left(t\right)$$

(2) $$F{\left(t\right)}_{SDS-CRISPR}=23.19\;C_{c}\left(t\right)+0.1990\;C_{u}\left(t\right)$$

Assuming that the sum of cleaved *C_c_* (*t*) and uncleaved *C_u_* (*t*) reporters equals the initial substrate concentration (*C*_0_), the fluorescence at time t can be simplified as:

(3) $$\begin{array}{ccc} F{\left(t\right)}_{canonical} & = & 24.47\;C_{c}\left(t\right)+0.1610\;\left(C_{0}-C_{c}\left(t\right)\right)\\ & = & 24.309C_{c}\left(t\right)+0.1610C_{0} \end{array}$$

(4) $$\begin{array}{ccc} F{\left(t\right)}_{SDS-CRISPR} & = & 23.19\;C_{c}\left(t\right)+0.1990\;\left(C_{0}-C_{c}\left(t\right)\right)\\ & = & 22.991C_{c}\left(t\right)+0.1990C_{0} \end{array}$$

The reaction velocity *dC_c_*/*dt* (nm s^−^^1^) was calculated as:

(5) $$\frac{dC_{c}}{dt}=\frac{1}{24.309}\times \frac{dF_{canonical}}{dt}$$

(6) $$\frac{dC_{c}}{dt}=\frac{1}{22.991}\times \frac{dF_{SDS-CRISPR}}{dt}$$

Initial reaction velocities (*v*_0_) for each substrate concentration were fitted to the Michaelis–Menten equation:

(7) $$v_{0}=v_{max}\frac{\left[S\right]}{K_{M}+\left[S\right]}=k_{cat}E_{0}\frac{\left[S\right]}{K_{M}+\left[S\right]}$$

where *k_cat_* is the catalytic turnover rate, *E*_0_ is the concentration of activated enzyme (50 nM in this experiment), [*S*] is the substrate concentration, and *K_M_* is the Michaelis–Menten constant. The Michaelis–Menten kinetics calculations for both assays are summarized in Figure 5i,j and Table S7.

### 4.8 AlphaFold3‐Based Structural Prediction of Cas12a RNP Complexes and Bioinformatic Analysis

Cas12a–crRNA–activator complex structures were predicted using AlphaFold3 with relevant sequences [37]. Each molecular species was entered separately, with copy numbers set to one for Cas12a, crRNA(s), and activator(s), two for Mg^2+^ ions, and ten for K^+^ ions. Calculations were performed using the auto‐seed setting. Model quality was assessed by pTM and ipTM metrics, with pTM scores > 0.5 indicating accurate overall folds and ipTM values > 0.8 reflecting confident interface predictions.

Structural comparison was performed by importing CIF models into PyMOL [42], aligning the complexes, and calculating the RMSD values. RMSD values > 2 Å were considered indicative of significant conformational differences. Key residue distances (e.g., Val377–Gln1136) were measured directly on 3D models. In addition, Euclidean distances for each residue of Cas12a (chain A) were computed across aligned structures to quantify local conformational changes. The code used for residue‐wise distance analysis is available in the Code Availability section.

The Euclidean distance *d* between corresponding residues *i* in two aligned structures was calculated as:

(8) $$d_{i}=\sqrt{{\left(x_{i,1}-x_{i,2}\right)}^{2}+{\left(y_{i,1}-y_{i,2}\right)}^{2}+{\left(z_{i,1}-z_{i,2}\right)}^{2}}$$

where (*x*_*i*,1_, *y*_*i*,1_, *z*_*i*,1_) and (*x*_*i*,2_, *y*_*i*,2_, *z*_*i*,2_) are the coordinates of residue *i* in structures 1 and 2, respectively.

### 4.9 qPCR Detection

qPCR was performed using the GoTaq Probe qPCR Master Mix (Promega) following the manufacturer's protocol. Each 20 µL reaction contained 1× Master Mix, 500 nM forward primer, 500 nM reverse primer, 250 nM hydrolysis probe, template DNA, and nuclease‐free water. Thermal cycling was carried out on a CFX96 Touch Real‐Time PCR Detection System (Bio‐Rad) with the following conditions: initial DNA polymerase activation at 95°C for 2 min, followed by 40 cycles of 95°C for 15 s and 60°C for 1 min. The assay used for clinical samples (qPCR‐pair1, Table S20) did not discriminate between IDH1^R132H^ and wild‐type alleles. The sensitivity of this assay is reported in Table S18. Each sample was tested in triplicate, and undetermined reactions without a Ct value were imputed as Ct = 40 for subsequent analysis.

### 4.10 Clinical Sample Preparation and SDS‐CRISPR Detection

DNA was extracted from clinical specimens, including FFPE sections (20 µm, two slides), fresh‐frozen tissue (∼25 mg), and plasma (1 mL), using the QIAamp DNA FFPE Tissue Kit, DNeasy Blood and Tissue Kit, or QIAamp Circulating Nucleic Acid Kit (QIAGEN), according to the manufacturers’ instructions.

RPA was performed with the TwistAmp Basic kit (TwistDx) at 39°C for 15 min. The subsequent SDS‐CRISPR *trans*‐cleavage reaction was carried out in 10 µL containing 1 × NEBuffer 4 (NEB), 200 nM crRNA (full‐sized or split), 100 nM Cas12a (Alt‐R), 5 µM FQ reporter, and 1 µL of RPA product. Reactions were incubated at 37°C for 5 min in a CFX96 Touch Real‐Time PCR System (Bio‐Rad), with fluorescence recorded every minute. For lateral‐flow detection, the reporter was replaced with 1 µM FB probe. The 10 µL CRISPR reaction was diluted in 40 µL HybriDetect assay buffer (Milenia Biotec) and applied to commercial test strips for readout.

### 4.11 Smartphone Application for Lateral‐Flow Strip Analysis

A custom smartphone application was implemented to support standardized interpretation of lateral‐flow strips. After completion of the assay, the strip was placed on a flat surface and imaged using the smartphone camera under ambient laboratory lighting conditions without additional optical components such as filters, external illumination modules, or lens attachments. The app identifies the test and control regions on each strip image and extracts their intensity profiles using OpenCV‐based algorithms. Average pixel values within the line boundaries are quantified, while surrounding areas are excluded as background. Results are returned as both numerical outputs and positive/negative classifications, with an adjustable threshold option for flexible use. For each assay, the app provides processed images, line intensity plots, and classification outcomes in real time. The frontend was developed in JavaScript/TypeScript with React Native (via Expo), and the backend was implemented in Python using the Django REST API.

### 4.12 Next‐Generation Sequencing and Data Processing

PCR amplicons spanning the IDH1^R132^ locus were purified using the MinElute PCR Purification Kit (QIAGEN) and submitted to Genewiz/Azenta for targeted amplicon sequencing. Libraries were prepared and sequenced in a 2 × 250 bp paired‐end configuration on an Illumina platform, generating approximately 50 000 high‐quality reads per sample. The vendor provided adapter‐trimmed and quality‐filtered clean reads along with integrated single‐nucleotide polymorphism and insertion/deletion calling.

For downstream analysis, all clean reads were aligned to the IDH1 reference sequence using a custom Python‐based pipeline. At the R132 position, nucleotide counts were extracted, and mutation abundance was quantified as the ratio of IDH1^R132H^ reads to wild‐type reads. Insertions and deletions were also recorded to ensure comprehensive variant profiling. The resulting variant allele frequencies were benchmarked against SDS‐CRISPR assay outputs to confirm concordance.

### 4.13 SDS‐CRISPR Assays for miRNA Detection

The SDS‐CRISPR *trans*‐cleavage reaction was performed in a final volume of 10 µL containing 1 × NEBuffer 4, 200 nM handle, 100 nM Alt‐R Cas12a, 5 µM reporter, 1 µL of 1 µM ssDNA activator (either full‐sized or split components), and 1 µL of synthetic miRNA (10 nM for verification; 10 nM–10 fM for sensitivity testing). Reactions were incubated at 37°C in a CFX96 Touch Real‐Time PCR Detection System for 60 min, with fluorescence recorded every minute.

### 4.14 SDS‐CRISPR Assays for HIV Mutation Detection

The SDS‐CRISPR *trans*‐cleavage reaction was performed in a final volume of 10 µL containing 1 × NEBuffer 4, 200 nM crRNA (full‐sized or split components), 100 nM Alt‐R Cas12a, 5 µM reporter, and 1 µL of 10 nM synthetic HIV dsDNA. Reactions were incubated at 37°C in a CFX96 Touch Real‐Time PCR Detection System for 60 min, with fluorescence recorded every minute.

### 4.15 Statistical Information

Normalized data used positive control–negative control scaling, defined as:

$$x_{norm}=\frac{x\;-\;x_{\;negative\;control}}{x_{\;positive\;control}\;-\;x_{\;negative\;control}}$$

Statistical analyses were performed using GraphPad Prism. Two‐tailed unpaired t‐tests, non‐parametric Mann–Whitney *U* tests, and Welch's *t*‐tests were applied as appropriate, with exact tests used for small sample sizes. A *p* value < 0.05 was considered significant. For all fluorescence‐based assays, the threshold was uniformly defined as the mean fluorescence of negative controls plus three s.d.

### 4.16 Code Availability

All custom code used in this study is openly available on GitHub.

#### 4.16.1 Calculation‐of‐Per‐Residue‐C‐Distance

Scripts for computing per‐residue Cα distance metrics on AlphaFold‐predicted Cas12a complexes.

https://github.com/XxinGuan/Calculation‐of‐per‐residue‐C‐distance

#### 4.16.2 NGS‐SNV‐Analysis

Amplicon‐NGS utilities for aligning clean reads and quantifying IDH1^R132H^ vs. wild‐type read counts in cfDNA.

https://github.com/XxinGuan/NGS‐SNV‐analysis

#### 4.16.3 Striplens Mobile App

Lateral‐flow strip analyzer (React Native/Expo frontend and Django REST backend) for automated control/ test line quantification.

https://github.com/ramchandra3101/strip_frontend

https://github.com/ramchandra3101/strip_backend

Unless otherwise noted in the individual repositories, the code is released under the MIT License.

## Conflicts of Interest

The authors declare no conflicts of interest.

## Supporting information




**Supporting File**: advs75149‐sup‐0001‐SuppMat.pdf.

## Data Availability

The data that supports the findings of this study are available in the supplementary material of this article.
