# Supplementary material for: SDS‐CRISPR for Single‐Nucleotide Variant Detection
Source: Adv Sci (Weinh). 2026 Apr 7;13(36):e75149. doi: 10.1002/advs.75149 (PMC13317634; doi:10.1002/advs.75149)
Supplement: Supplementary file 1 — Supporting File: advs75149‐sup‐0001‐SuppMat.pdf. [file ADVS-13-e75149-s001.pdf]

Supporting Information

SDS-CRISPR for Single-Nucleotide Variant Detection

Xin Guan[1,2], Chong Guo[1,2], Jiongyu Zhang[1], Rui Yang[1,2], Yerramsetti Ramachandra[1], Chengyu Hou[1,2], Minjie Pei[1,2], Shuo Zhang[1,2], Kurt T. Schalper[1,2], Xingye Liu[1,2], Qian Wu[3], Ketan R. Bulsara[4], and Changchun Liu[1]*

1. *Department of Biomedical Engineering, University of Connecticut Health Center, Farmington, Connecticut 06030, USA*
2. *Department of Biomedical Engineering, University of Connecticut, Storrs, Connecticut 06269, USA*
3. *Pathology and Laboratory Medicine, University of Connecticut Health Center, Farmington, Connecticut 06030, USA*
4. *Department of Neurosurgery, University of Connecticut Health Center, Farmington, Connecticut 06030, USA*

*** Corresponding author**

Dr. Changchun Liu
Department of Biomedical Engineering
University of Connecticut Health Center
263 Farmington Avenue
Farmington, CT 06030
Phone: (860)-679-2565
E-mail: chaliu@uchc.edu

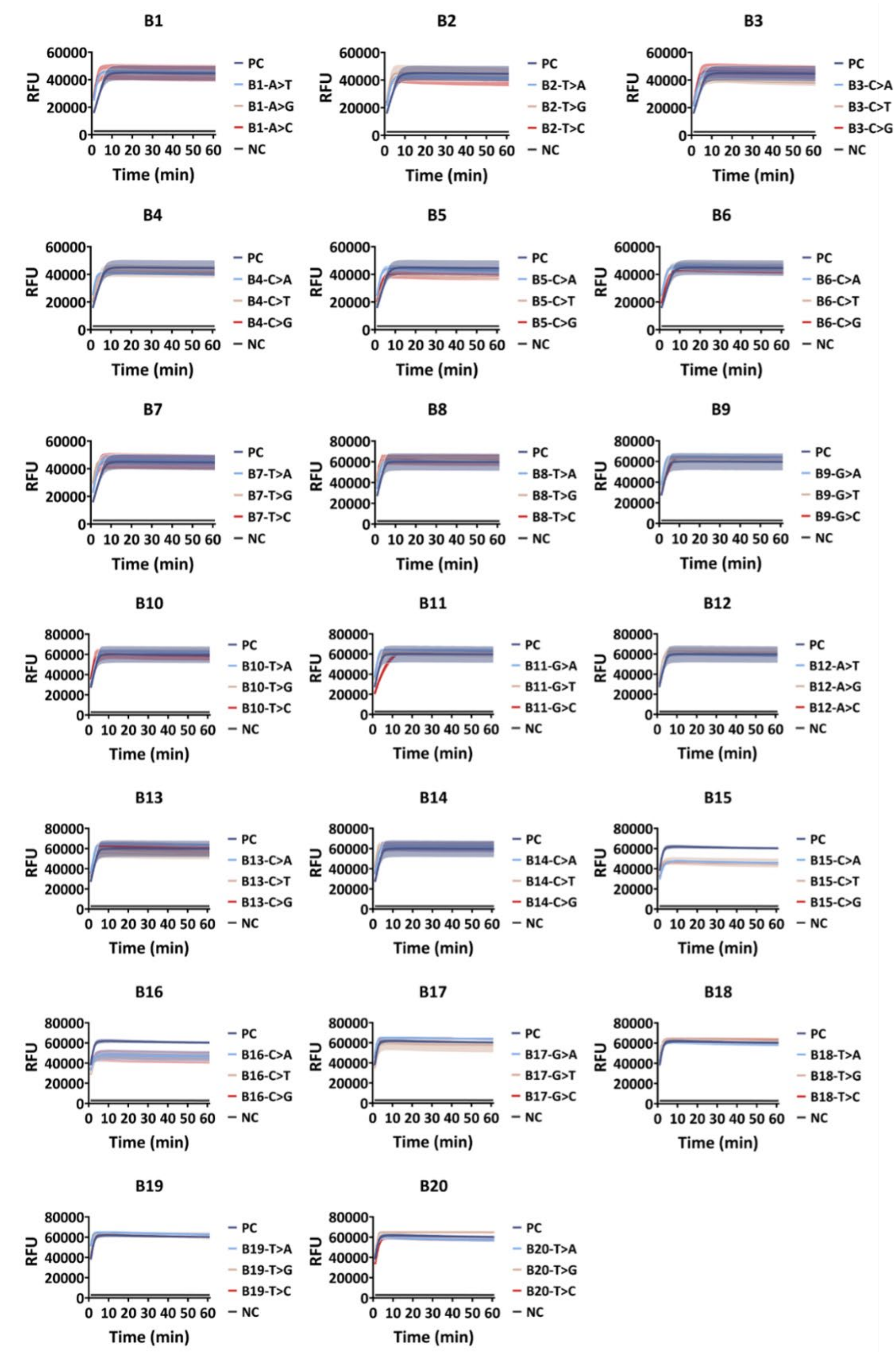

Supplementary Figure 1. Real-time fluorescence kinetics of Cas12a collateral cleavage with canonical CRISPR. Each plot includes the perfect-match control (PC), three SNV variants, and a no-template control (NC). Data represent mean ± s.d. from three technical replicates (n = 3).

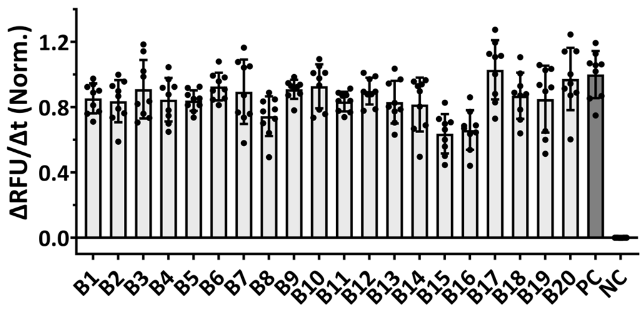

Supplementary Figure 2. Normalized fluorescence growth rates with canonical CRISPR. Growth rates during the first 5 min were calculated using positive (perfect-match) and negative (template-free) controls. Each SNV site includes three mutation types with three replicates per type (n = 9).

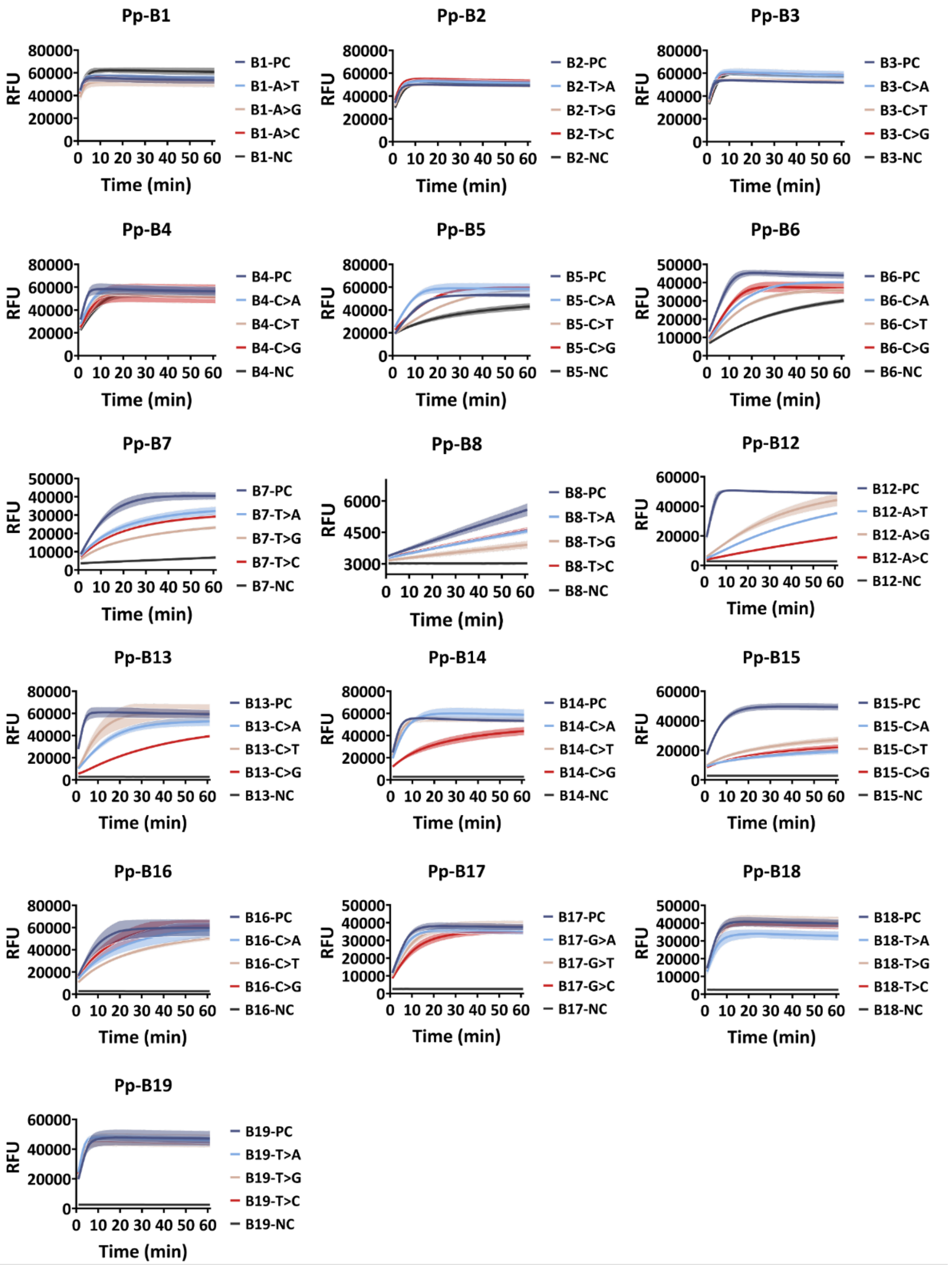

Supplementary Figure 3. Real-time fluorescence kinetics of Cas12a collateral cleavage using the *actSplit-Pp* strategy (additional SNV sites not shown in the main figures). Each plot includes the perfect-match control (PC), three SNV variants, and a no-template control (NC). Data represent mean ± s.d. from three technical replicates (n = 3).

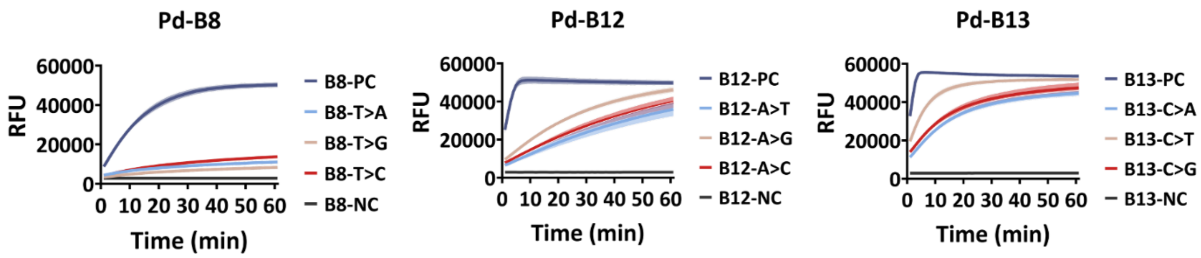

Supplementary Figure 4. Real-time fluorescence kinetics of Cas12a collateral cleavage using the *actSplit-Pd* strategy (additional SNV sites not shown in the main figures). Each plot includes the perfect-match control (PC), three SNV variants, and a no-template control (NC). Data represent mean ± s.d. from three technical replicates (n = 3).

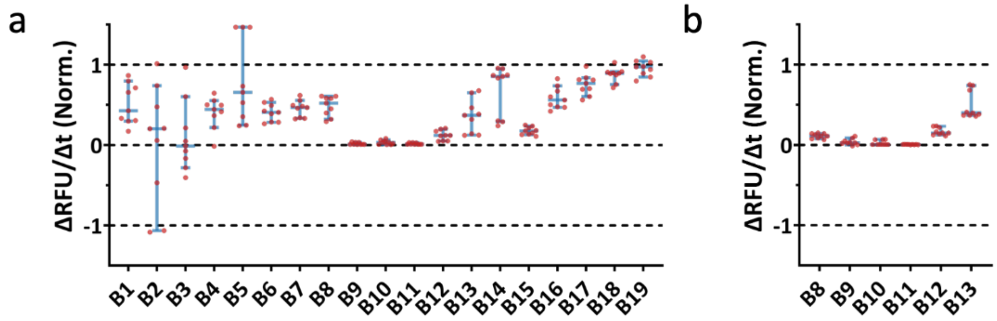

Supplementary Figure 5. Normalized fluorescence growth rates using the *actSplit* strategy. Growth rates during the first 5 min were calculated using positive (perfect-match) and negative (template-free) controls. Each SNV site includes three mutation types with three replicates per type (n = 9). Data are presented as the median with 95% confidence intervals. (a) Results correspond to Supplementary Figure 3 (*actSplit-Pp* strategy). (b) Results correspond to Supplementary Figure 4 (*actSplit-Pd* strategy).

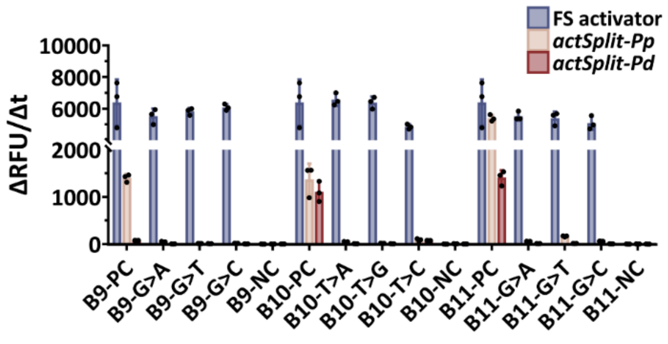

Supplementary Figure 6. Fluorescence growth rates for full-sized and *actSplit* activators at positions B9–B11. Fluorescence growth rate (0–5 min) for SNVs at positions B9, B10, and B11 under three detection designs: full-sized activator (FS activator), *actSplit-Pp,* and *actSplit-Pd*. Data represent mean ± s.d. from three technical replicates (n = 3).

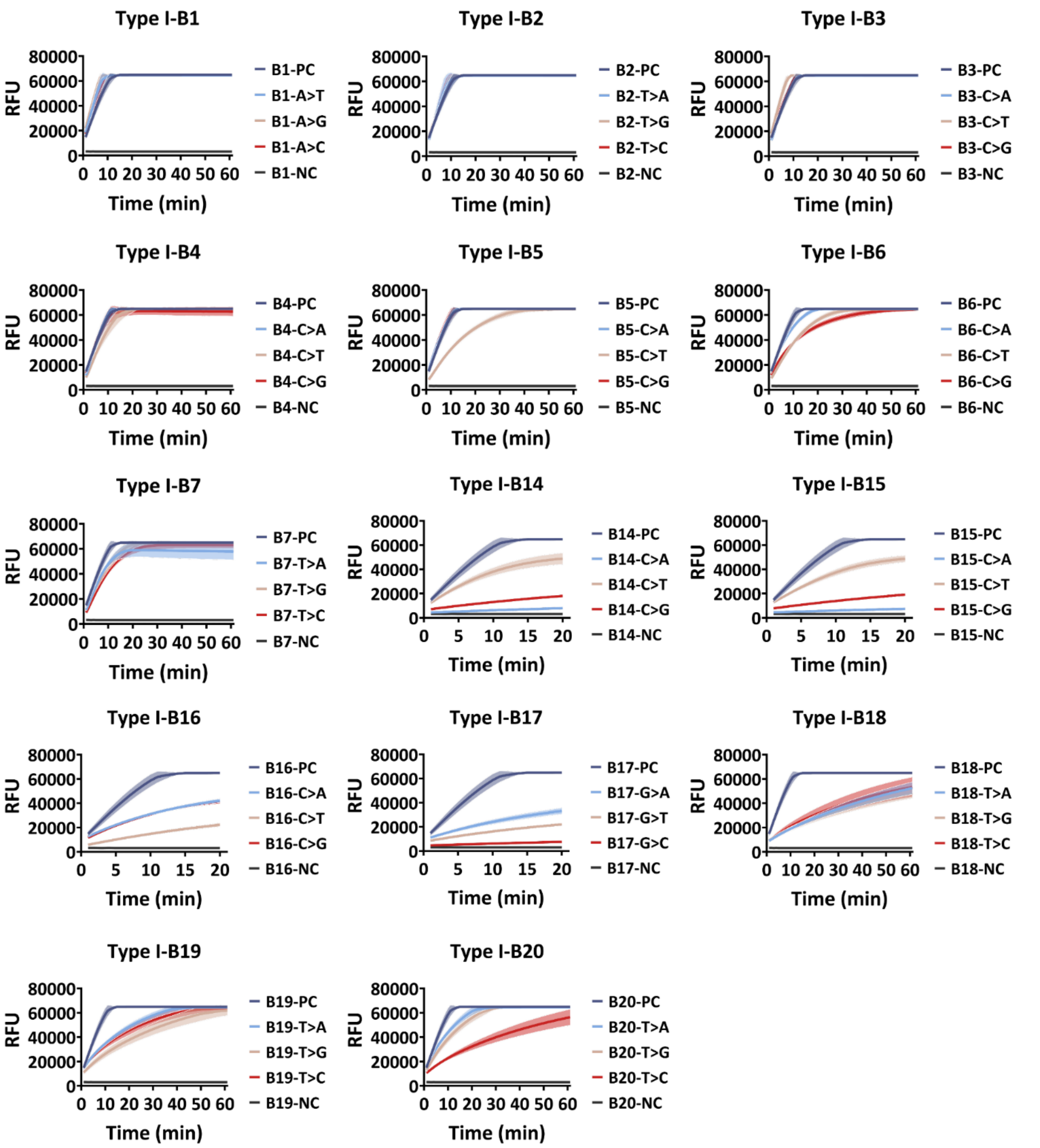

Supplementary Figure 7. Real-time fluorescence kinetics of Cas12a collateral cleavage using the *crSplit-Type I* strategy (additional SNV sites not shown in the main figures). Each plot includes the perfect-match control (PC), three SNV variants, and a no-template control (NC). Data represent mean ± s.d. from three technical replicates (n = 3).

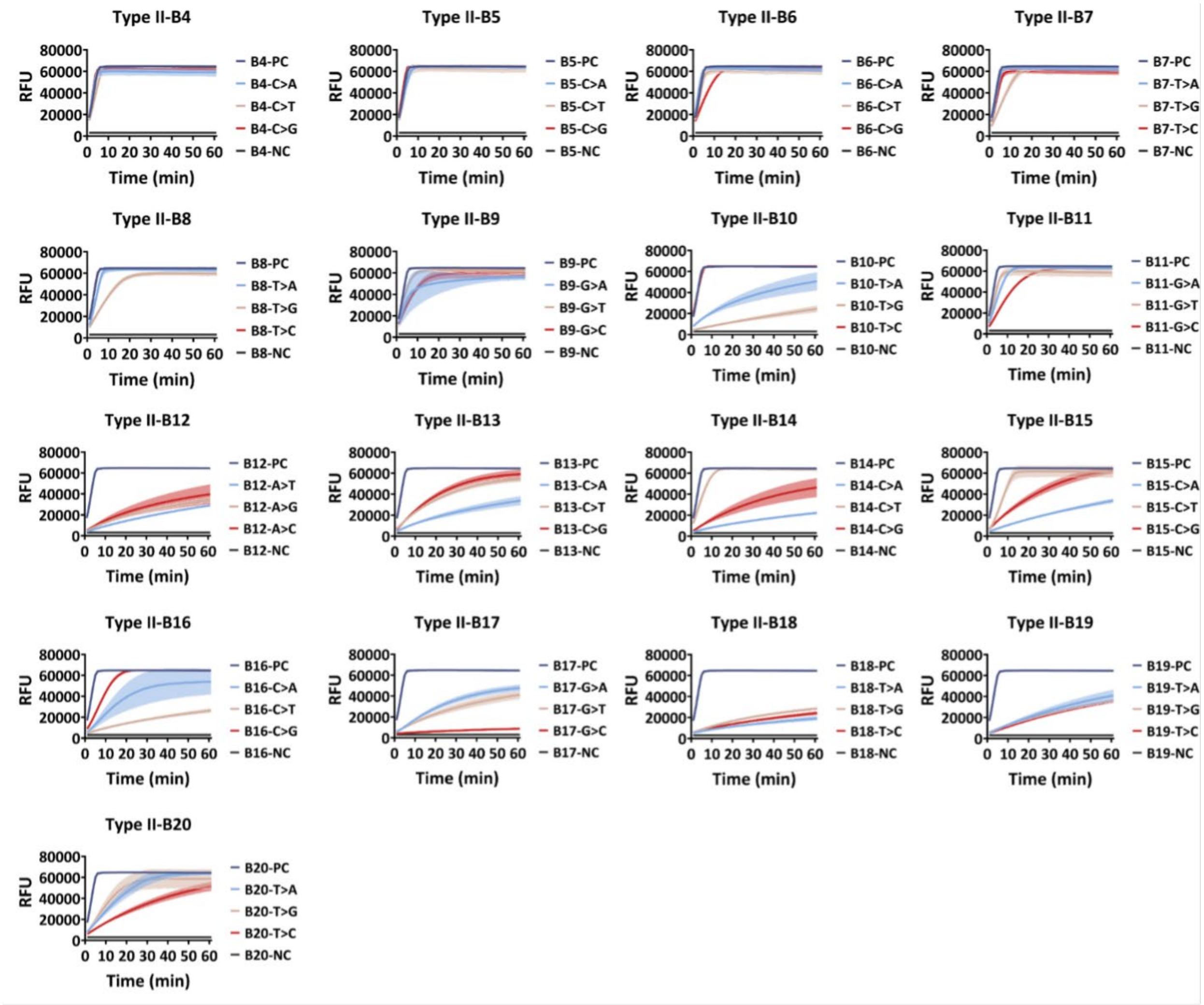

Supplementary Figure 8. Real-time fluorescence kinetics of Cas12a collateral cleavage using the *crSplit-Type II* strategy (additional SNV sites not shown in the main figures). Each plot includes the perfect-match control (PC), three SNV variants, and a no-template control (NC). Data represent mean ± s.d. from three technical replicates (n = 3).

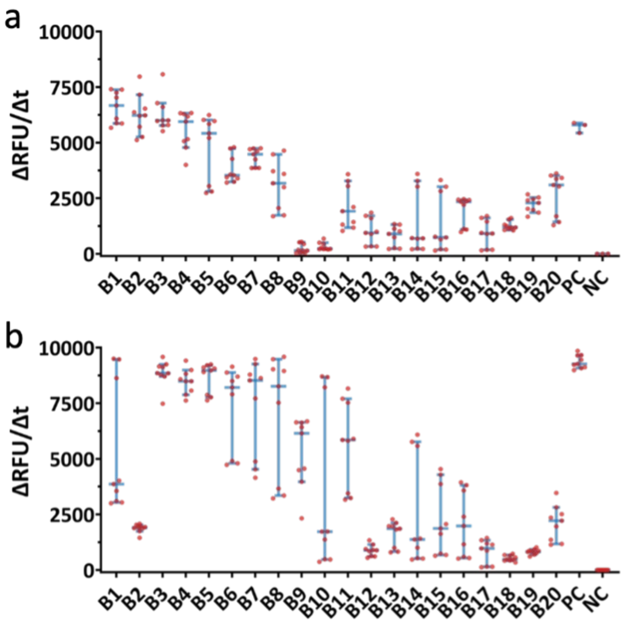

Supplementary Figure 9. Normalized fluorescence growth rates using the *crSplit* strategy. Growth rates during the first 5 min were calculated using positive (perfect-match) and negative (template-free) controls. Each SNV site includes three mutation types with three replicates per type (n = 9). Data are presented as the median with 95% confidence intervals. (a) Results correspond to Supplementary Figure 7 (*crSplit-Type I* strategy). (b) Results correspond to Supplementary Figure 8 (*crSplit-Type II* strategy).

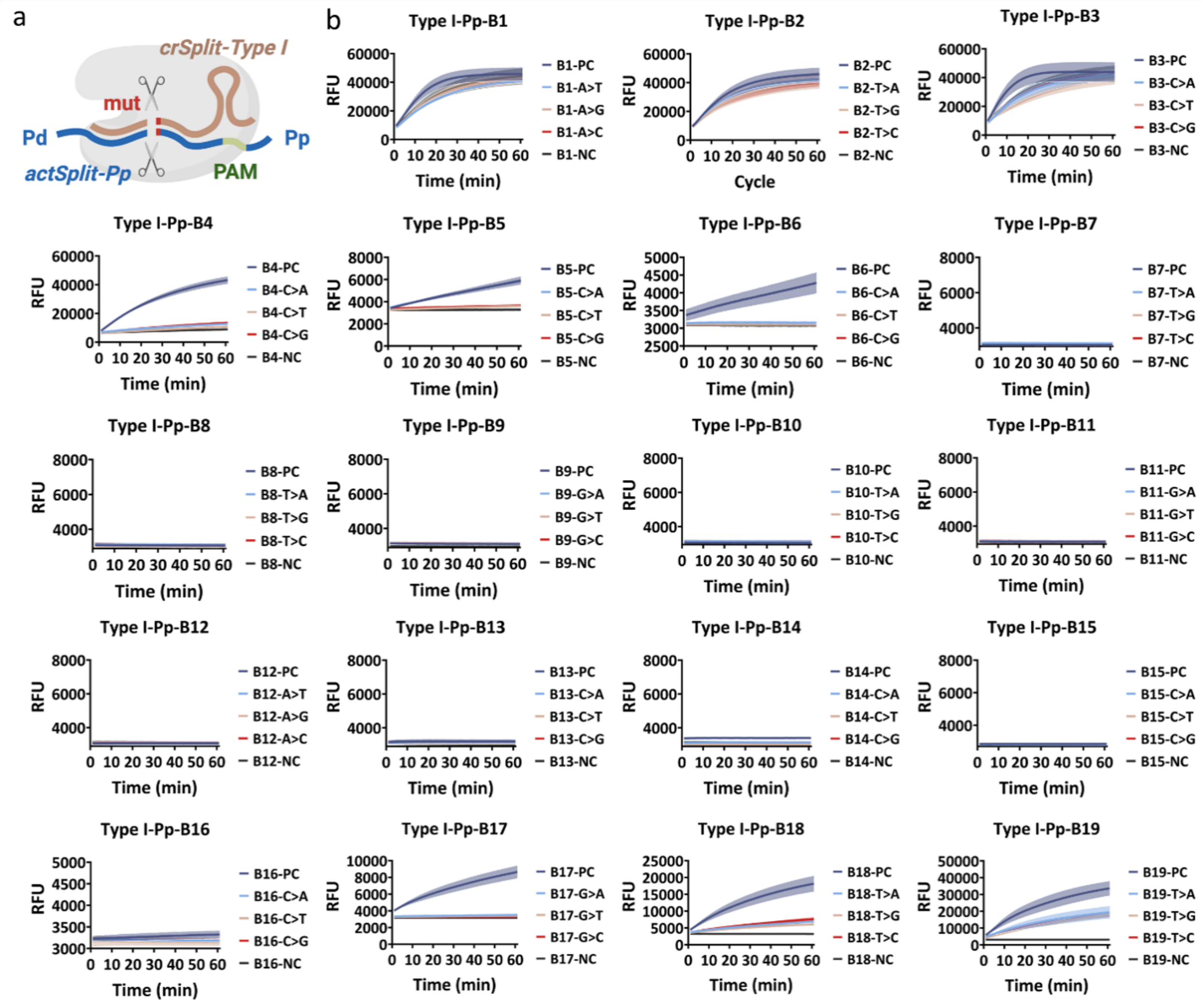

Supplementary Figure 10. Real-time fluorescence kinetics of Cas12a collateral cleavage integrating the *actSplit-Pp* and *crSplit-Type I* strategies. (a) Schematic of the SDS-CRISPR system integrating the *actSplit-Pp* and *crSplit-Type I* strategies. (b) Each plot includes the perfect-match control (PC), three SNV variants, and a no-template control (NC). Data represent mean ± s.d. from three technical replicates (n = 3).

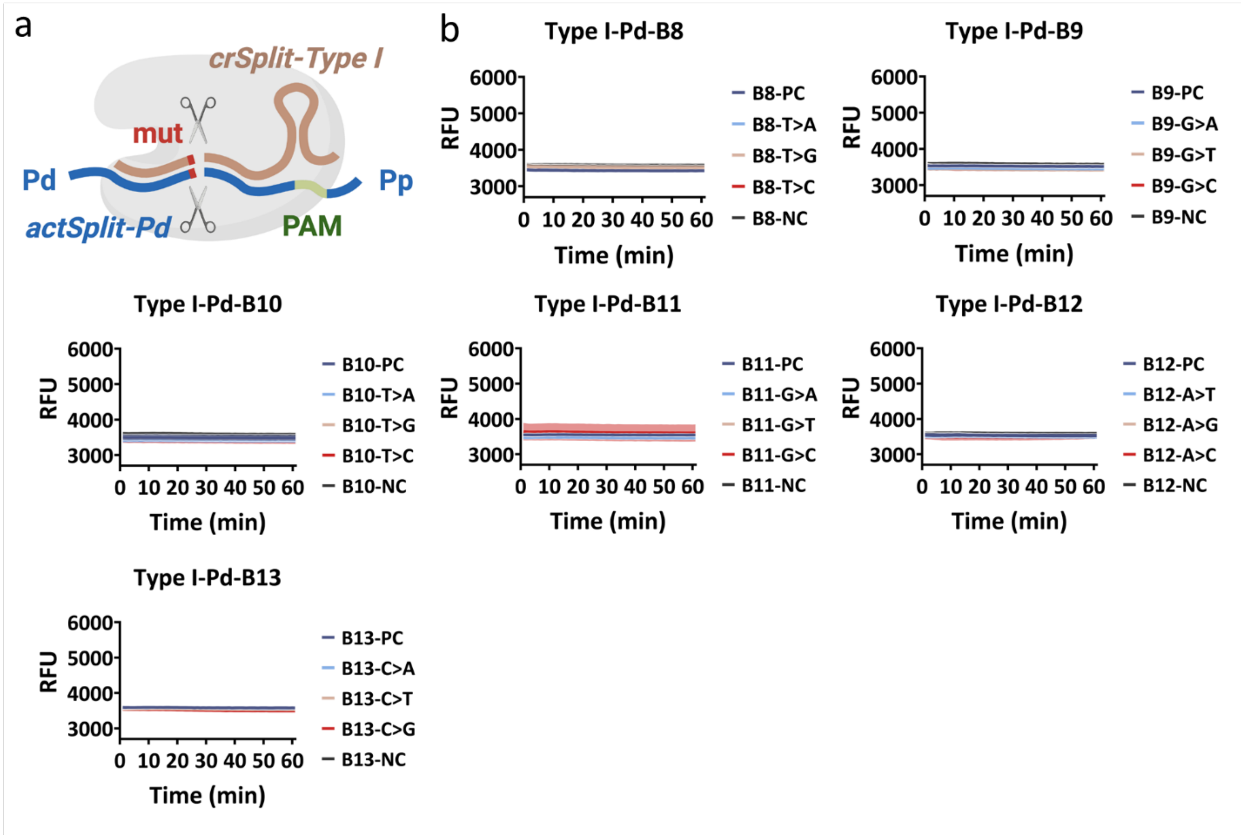

Supplementary Figure 11. Real-time fluorescence kinetics of Cas12a collateral cleavage integrating the *actSplit-Pd* and *crSplit-Type I* strategies. (a) Schematic of the SDS-CRISPR system integrating the *actSplit-Pd* and *crSplit-Type I* strategies. (b) Each plot includes the perfect-match control (PC), three SNV variants, and a no-template control (NC). Data represent mean ± s.d. from three technical replicates (n = 3).

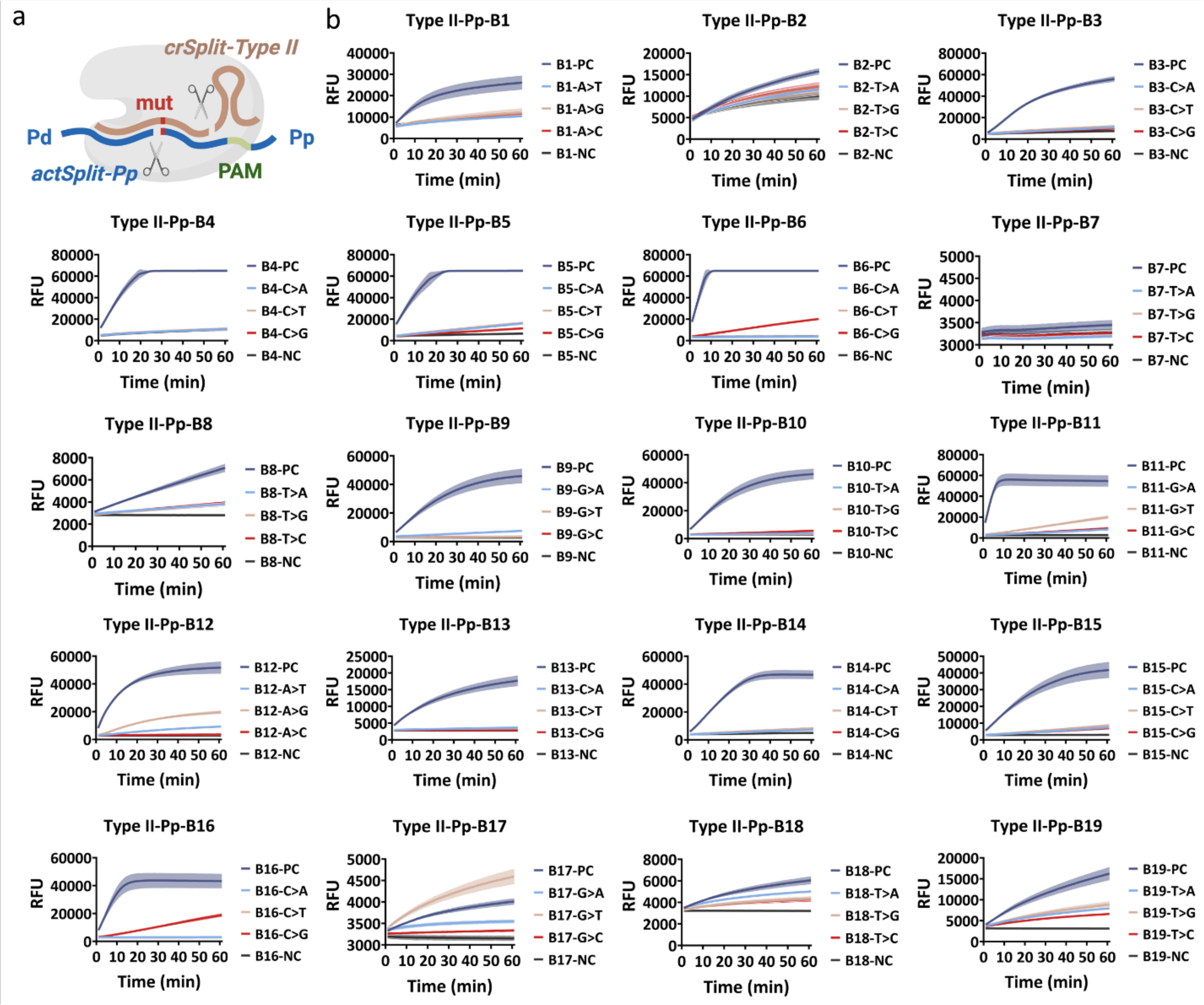

Supplementary Figure 12. Real-time fluorescence kinetics of Cas12a collateral cleavage integrating the *actSplit-Pp* and *crSplit-Type II* strategies. (a) Schematic of the SDS-CRISPR system integrating the *actSplit-Pp* and *crSplit-Type II* strategies. (b) Each plot includes the perfect-match control (PC), three SNV variants, and a no-template control (NC). Data represent mean ± s.d. from three technical replicates (n = 3).

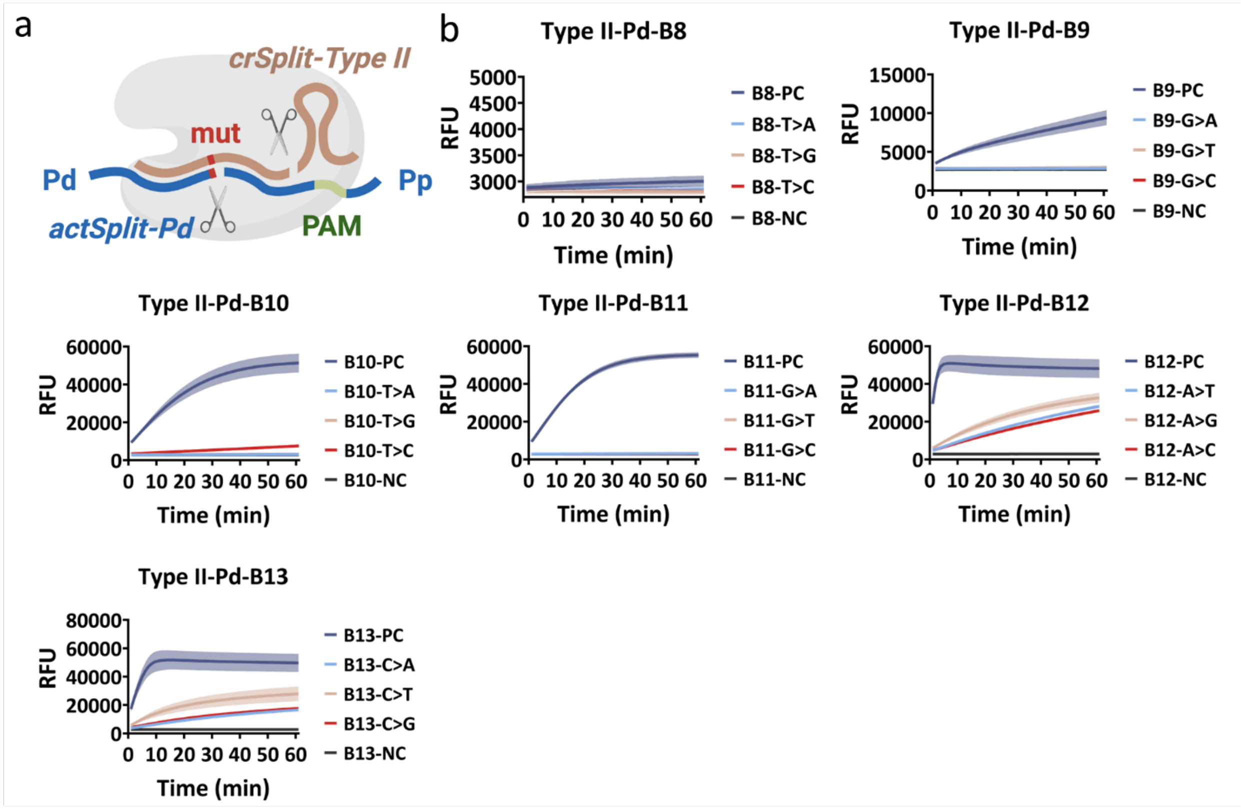

Supplementary Figure 13. Real-time fluorescence kinetics of Cas12a collateral cleavage integrating the *actSplit-Pd* and *crSplit-Type II* strategies. (a) Schematic of the SDS-CRISPR system integrating the *actSplit-Pd* and *crSplit-Type II* strategies. (b) Each plot includes the perfect-match control (PC), three SNV variants, and a no-template control (NC). Data represent mean ± s.d. from three technical replicates (n = 3).

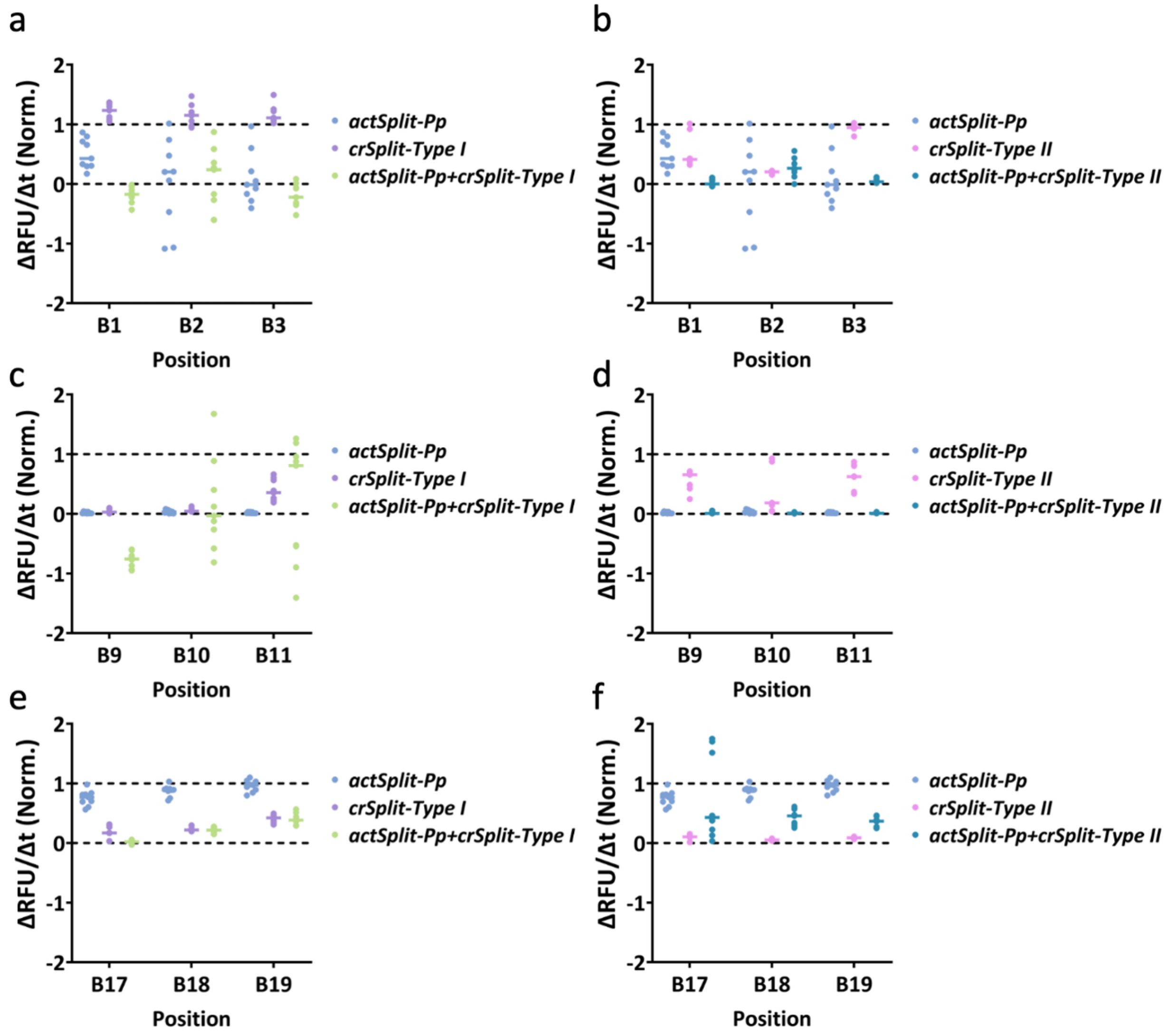

Supplementary Figure 14. Normalized fluorescence growth rates using different split strategies. Growth rates during the first 5 min were normalized using both positive (perfect-match) and negative (template-free) controls. Each SNV site includes three mutation types with three replicates per type (n = 9). The horizontal line indicates the median value for each group. (a, c, e) Comparison of *actSplit-Pp*, *crSplit-Type I*, and *actSplit-Pp* + *crSplit-Type I* at positions (a) B1–B3, (c) B9–B11, and (e) B17–B19. b, d, f, Comparison of *actSplit-Pp*, *crSplit-Type II*, and *actSplit-Pp* + *crSplit-Type II* at positions (b) B1–B3, (d) B9–B11, and (f) B17–B19.

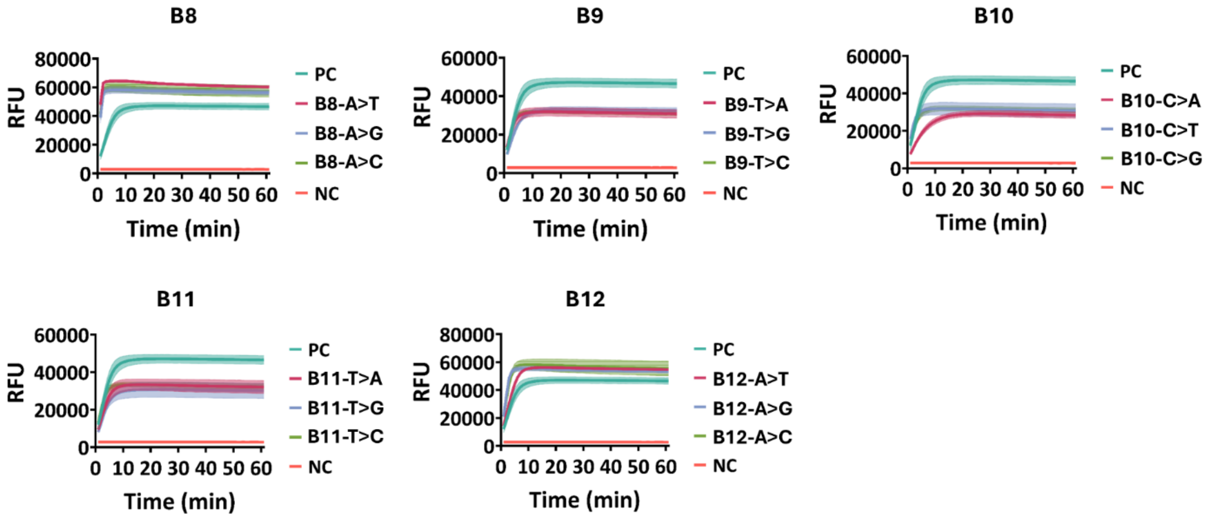

Supplementary Figure 15. Real-time fluorescence kinetics of Cas12a collateral cleavage with canonical CRISPR (2nd Target). Each plot includes the perfect-match control (PC), three SNV variants, and a no-template control (NC). Data represent mean ± s.d. from three technical replicates (n = 3).

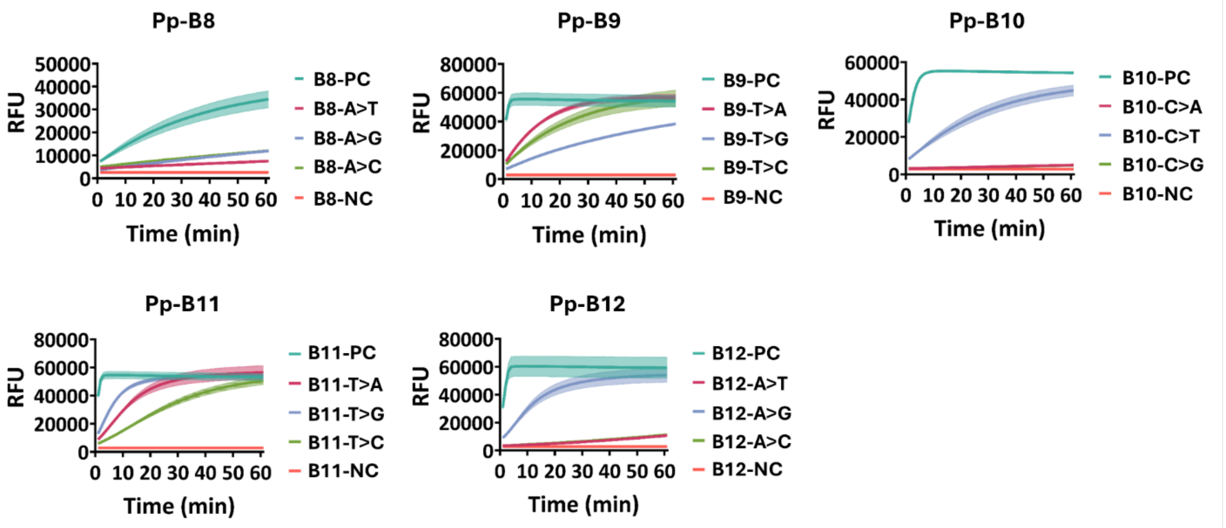

Supplementary Figure 16. Real-time fluorescence kinetics of Cas12a collateral cleavage using the *actSplit-Pp* strategy (2nd Target). Each plot includes the perfect-match control (PC), three SNV variants, and a no-template control (NC). Data represent mean ± s.d. from three technical replicates (n = 3).

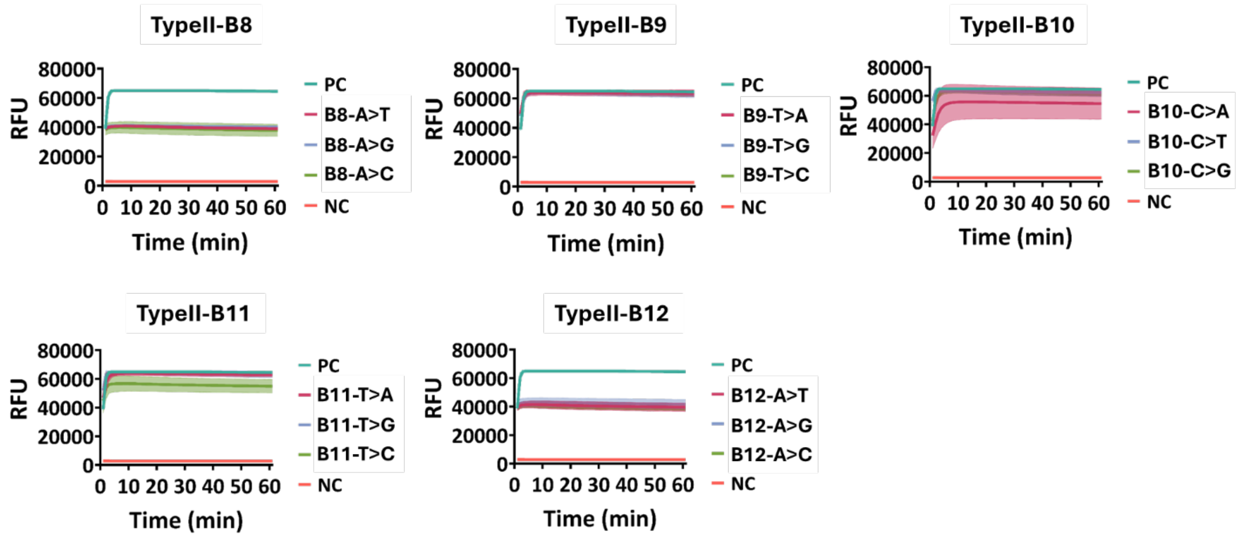

Supplementary Figure 17. Real-time fluorescence kinetics of Cas12a collateral cleavage using the *crSplit-Type II* strategy (2nd Target). Each plot includes the perfect-match control (PC), three SNV variants, and a no-template control (NC). Data represent mean ± s.d. from three technical replicates (n = 3).

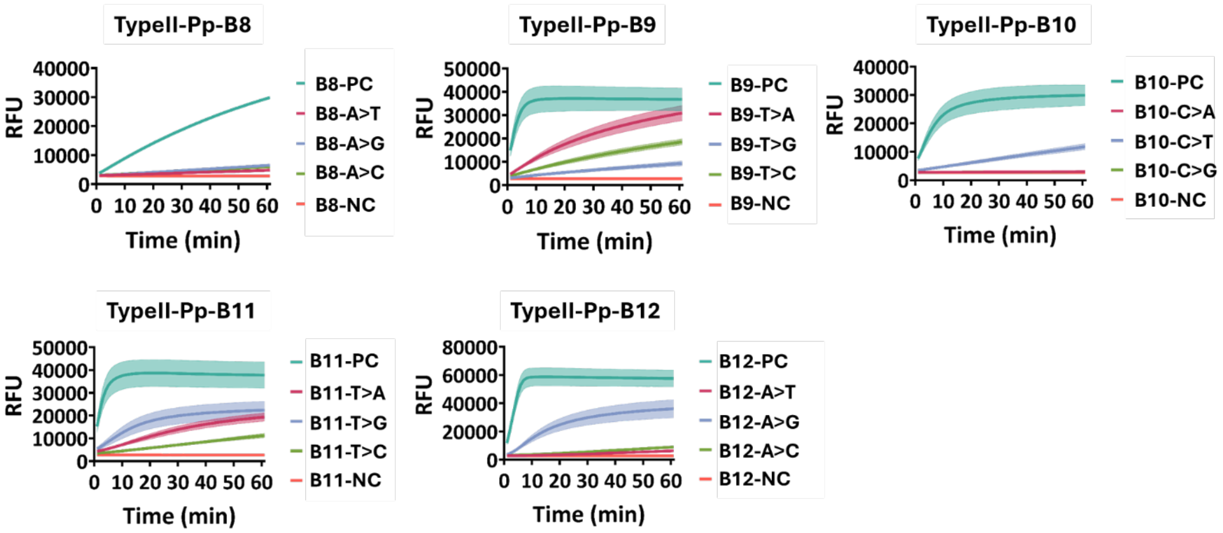

Supplementary Figure 18. Real-time fluorescence kinetics of Cas12a collateral cleavage integrating the *actSplit-Pp* and *crSplit-Type II* strategies (2nd Target). Each plot includes the perfect-match control (PC), three SNV variants, and a no-template control (NC). Data represent mean ± s.d. from three technical replicates (n = 3).

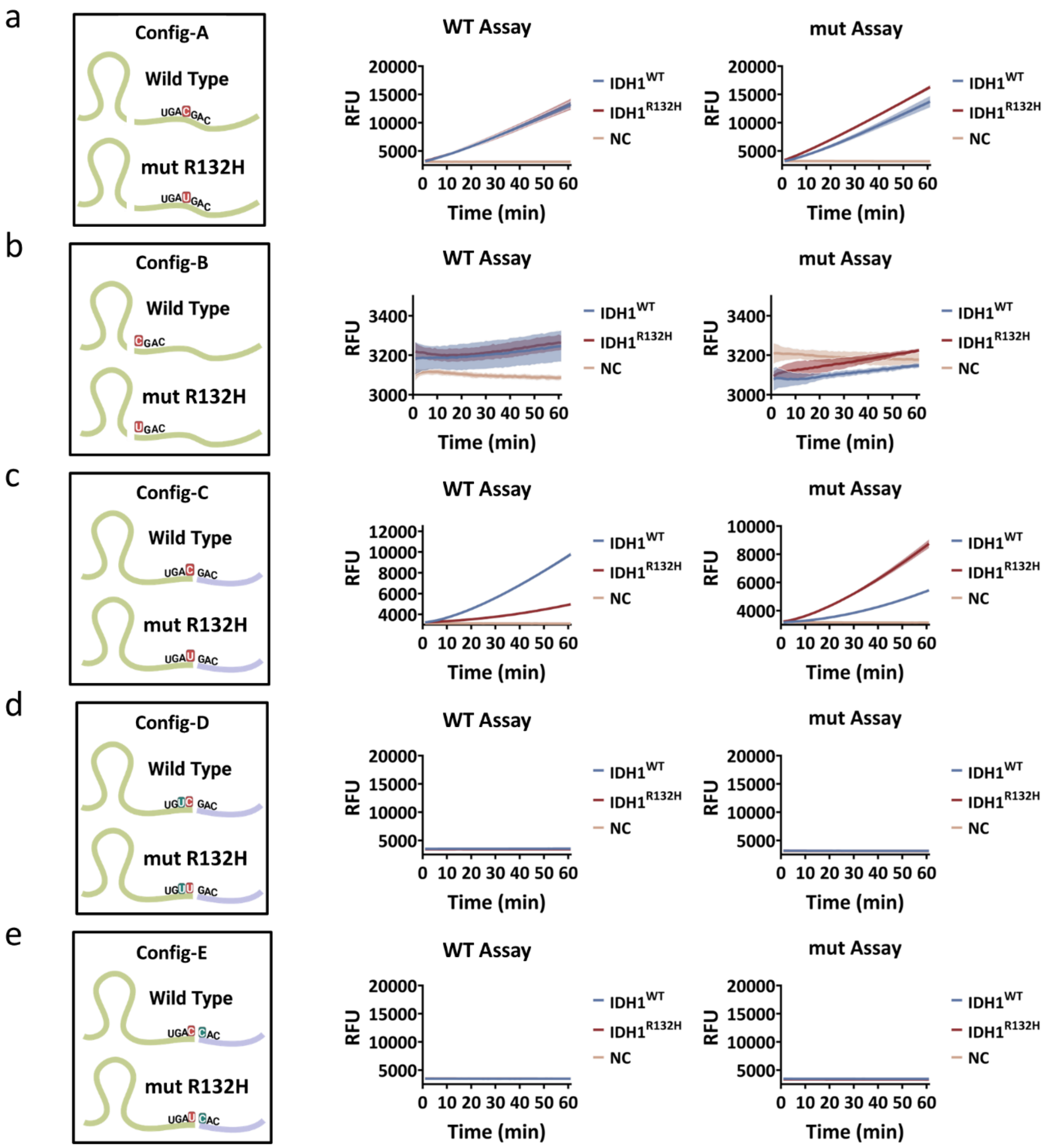

Supplementary Figure 19. Real-time fluorescence kinetics of SDS-CRISPR assays using different crRNA and activator configurations to distinguish $IDH1^{WT}$ and $IDH1^{R132H}$. (a–e) Schematic representations and corresponding fluorescence kinetics for five distinct assay designs (Config-A to Config-E). For each configuration, wild-type and mutant targets were tested using both wild-type (WT) and mutant (mut) assays. Each plot includes $IDH1^{WT}$, $IDH1^{R132H}$, and a negative control (NC). Data represent mean ± s.d. from three technical replicates (n = 3).

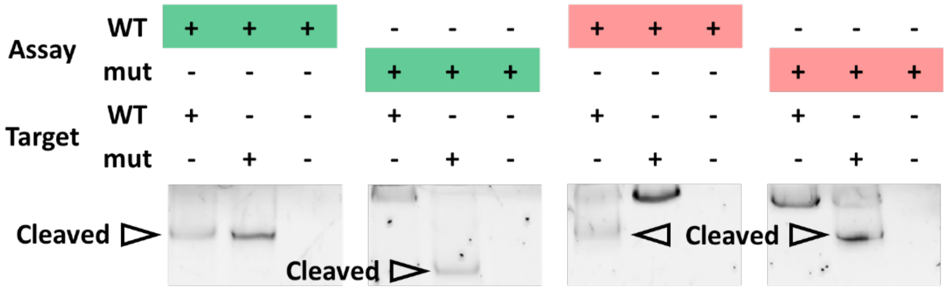

Supplementary Figure 20. *Cis*-cleavage patterns of canonical and SDS-CRISPR assays. Representative gel images comparing canonical (green) and SDS-CRISPR (red) configurations. Cleaved products are indicated by arrowheads. Images were adjusted with Light +0% and Contrast +20% for clarity.

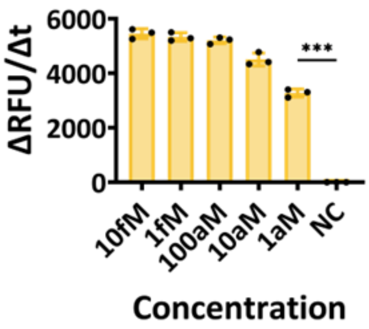

Supplementary Figure 21. Sensitivity analysis of the RPA assay. Fluorescence growth rates for a range of wild-type target concentrations were measured using the SDS-CRISPR IDH1 wild-type assay. Statistical significance between the 1aM group and the negative control (NC) was assessed using Welch's two-tailed t-test ($p = 0.0007$).

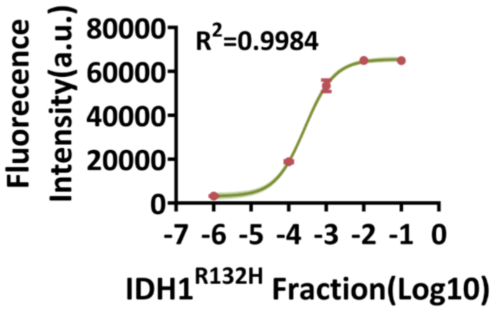

Supplementary Figure 22. Dose–response curve of the SDS-CRISPR assay for IDH1^{R132H} detection. Fluorescence intensity is plotted as a function of IDH1^{R132H} mutant allele fraction ($\log_{10}$ scale) across a dilution series (n = 15). Data points represent mean ± s.d. of technical replicates. Nonlinear regression using a four-parameter variable-slope model yielded an EC_{50} of 2.7×10^{-4} (95% confidence interval [CI]: 2.38×10^{-4}–3.05×10^{-4}), a Hill slope of 1.10 (95% CI: 1.00–1.21), a span of 62,485, and asymptotic fluorescence values of 3,109 (bottom) and 65,595 (top). The fit showed excellent agreement with the data ($R^2 = 0.9984$; 11 degrees of freedom).

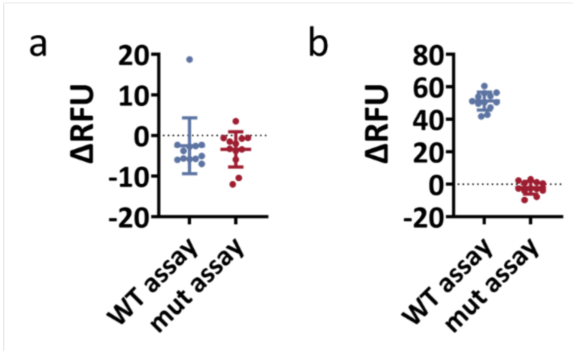

Supplementary Figure 23. Background signals from negative controls in SDS-CRISPR assays. (a) ΔRFU values from no-template controls (NFW) tested in wild-type (WT) and mutant (mut) assays (n = 12 each). (b) ΔRFU values from RPA reactions performed without template DNA (RPA product, no template) tested in WT and mut assays (n = 12 each). Data are shown as mean ± s.d., with dotted lines indicating baseline levels. Both no-template and RPA (no-template) controls produced signals near background, demonstrating the high specificity and low background of the assay.

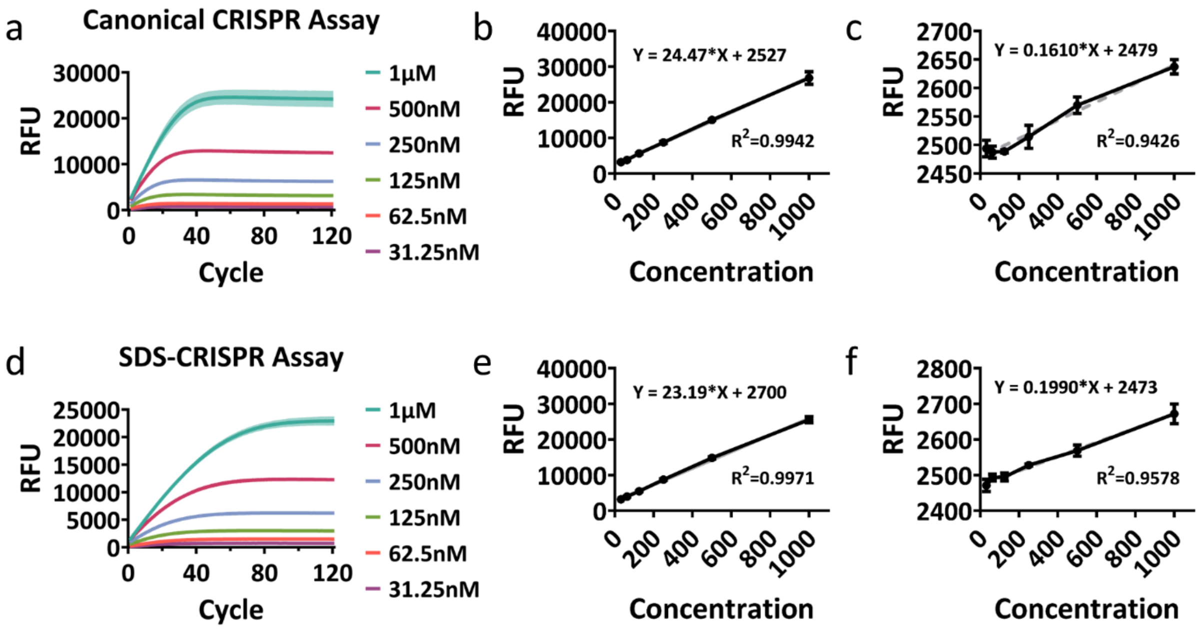

Supplementary Figure 24. Michaelis–Menten kinetic analysis of canonical CRISPR and SDS-CRISPR assays (IDH1^{R132H}-directed). (a–c) Canonical assay: (a) Real-time fluorescence kinetics at varying reporter concentrations (1 μM to 31.25 nM); (b) Linear fitting results for cleaved reporters ($F_c(t)$); (c) Linear fitting results for uncleaved reporters ($F_u(t)$). (d–f) SDS-CRISPR assay: (d) Real-time fluorescence kinetics at varying reporter concentrations; (e) Linear fitting results for cleaved reporters; (f) Linear fitting results for uncleaved reporters. Data points represent mean ± s.d. of technical replicates.

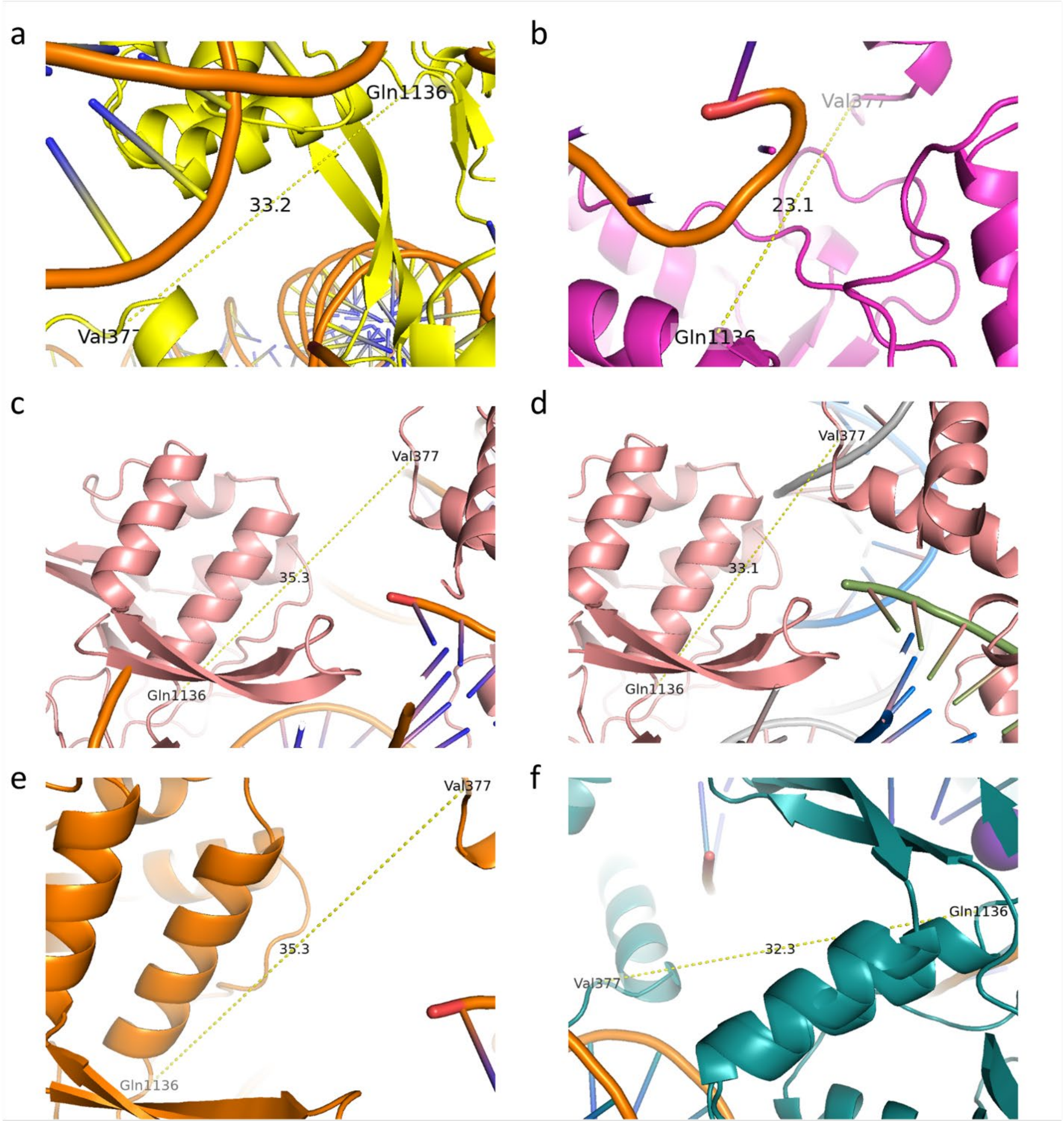

Supplementary Figure 25. Structural analysis of Cas12a activation states by measuring the distance between Val377 and Gln1136. (a–f) Representative AlphaFold3-predicted structures[1] (CIF files) showing the spatial arrangement and measured distances (in Å) between Val377 and Gln1136 under different assay conditions. (a) Positive control (33.2 Å); (b) Negative control (23.1 Å); (c) Wild-type assay + wild-type target (35.3 Å); (d) Wild-type assay + mutant target (33.1 Å); (e) Mutant assay + mutant target (35.3 Å); (f) Mutant assay + wild-type target (32.3 Å).

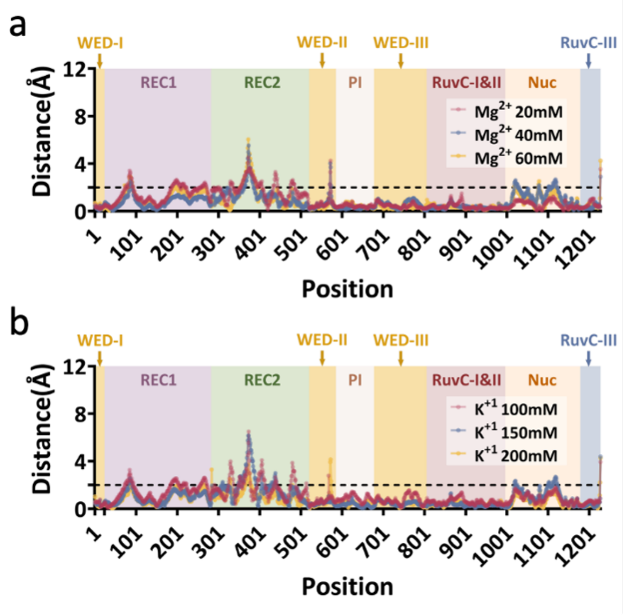

Supplementary Figure 26. Per-residue Euclidean distances (Å) between Cα atoms of LbCas12a structures predicted under the IDH1^{WT}-targeting SDS-CRISPR assay. (a) Comparison of elevated Mg^{2+} concentrations (20, 40, and 60 mM) to the baseline 10 mM condition. (b) Comparison of elevated K^{+} concentrations (100, 150, and 200 mM) to the baseline 50 mM condition. All structures were modeled in the presence of the mutant target.

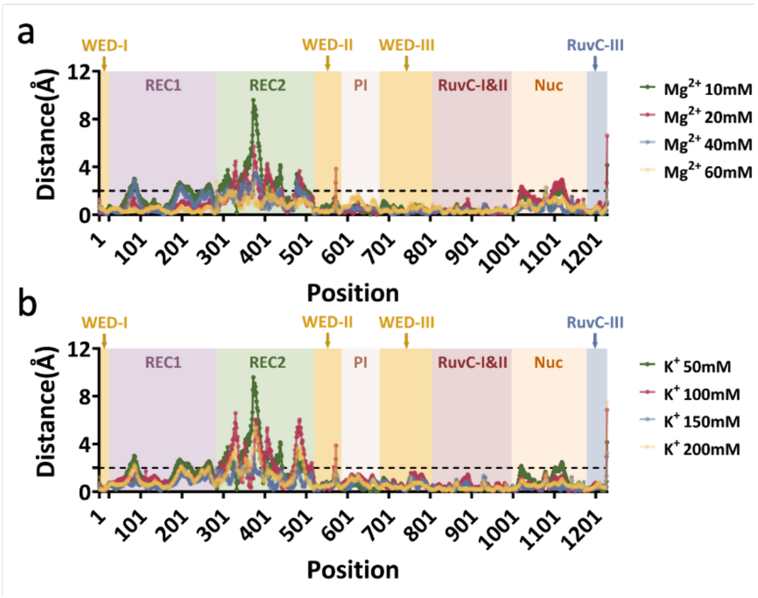

Supplementary Figure 27. Per-residue Euclidean distances (Å) between Cα atoms of LbCas12a structures predicted under the IDH1^{WT}-targeting SDS-CRISPR assay, comparing wild-type and mutant targets at varying ionic concentrations. (a) Mg^{2+} concentrations (10, 20, 40, and 60 mM). (b) K^{+} concentrations (50, 100, 150, and 200 mM).

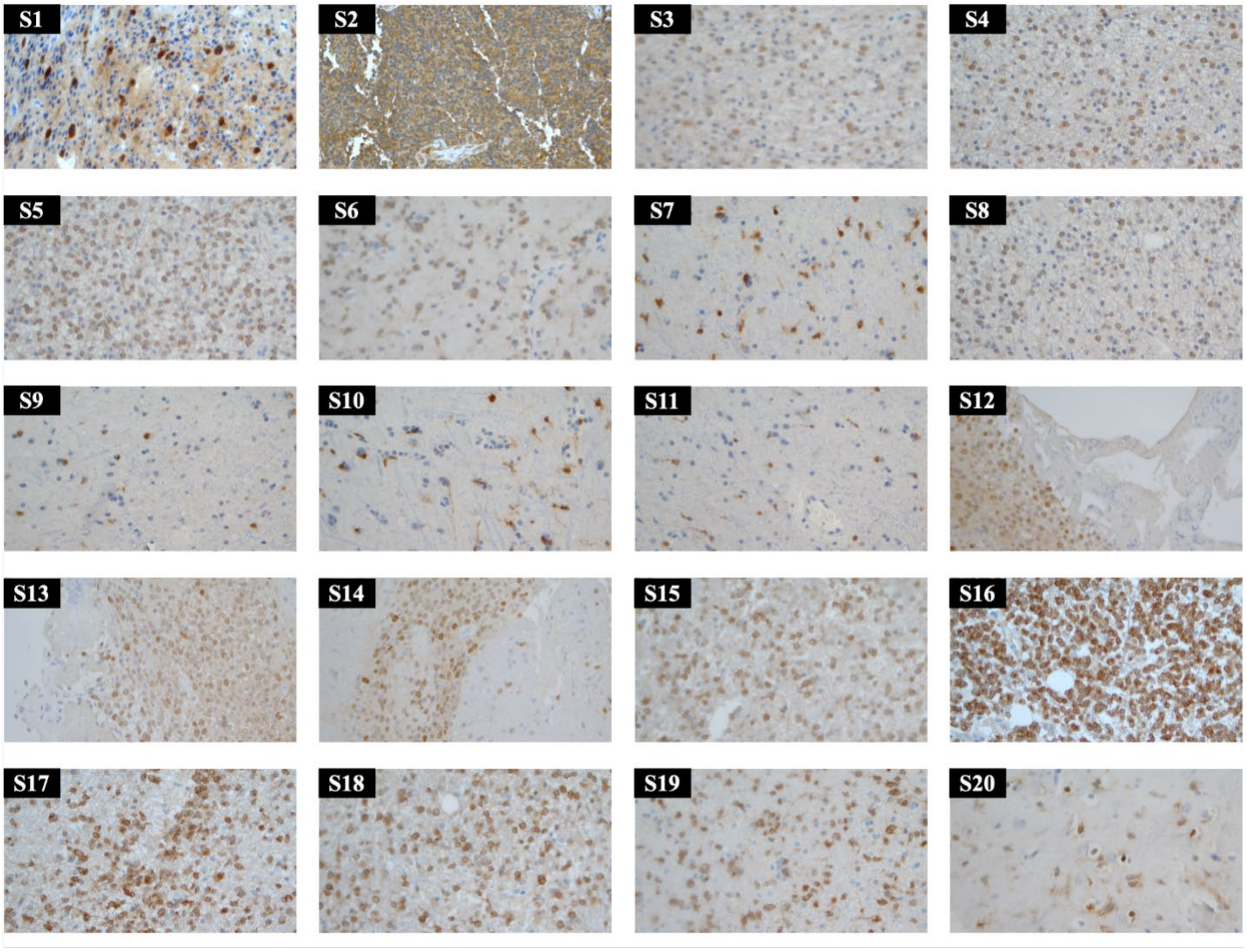

Supplementary Figure 28. IHC images of all FFPE glioma specimens used in this study (S1–S20). IHC staining for each individual sample is shown.

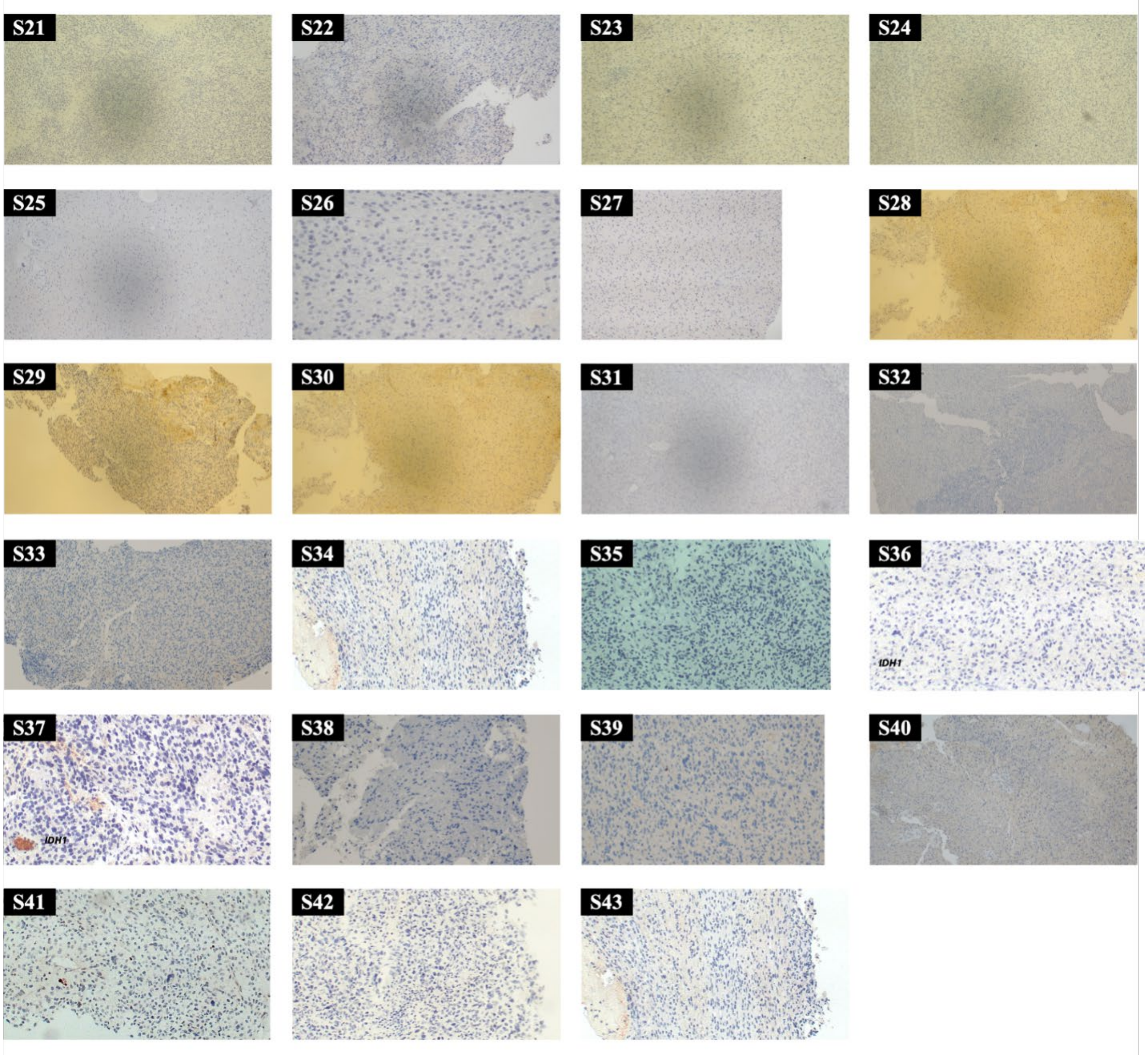

Supplementary Figure 29. IHC images of all FFPE glioma specimens used in this study (S21–S43). IHC staining for each individual sample is shown.

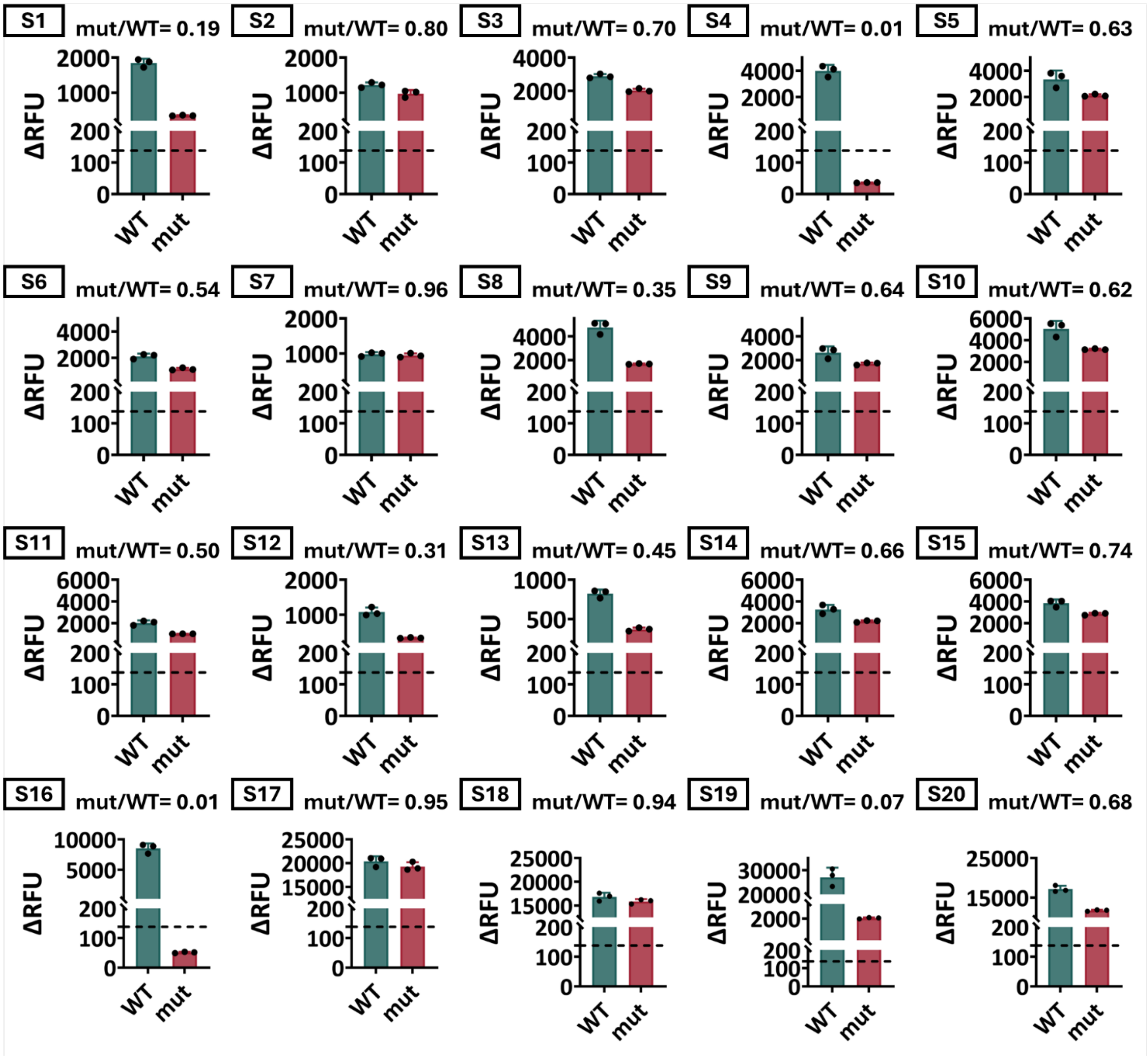

Supplementary Figure 30. Individual ΔRFU responses and mutant-to-wild-type ratios for IDH1^{R132H}-positive samples. Bar plots show ΔRFU signals for each of the 20 IHC-verified IDH1^{R132H}-positive FFPE glioma samples (S1–S20), with the corresponding mutant-to-wild-type (mut/WT) ratios indicated above each panel. Each bar represents the mean ± s.d. of three technical replicates. The dashed line marks the cutoff value (138), defined as the mean + 3×s.d. of wild-type samples.

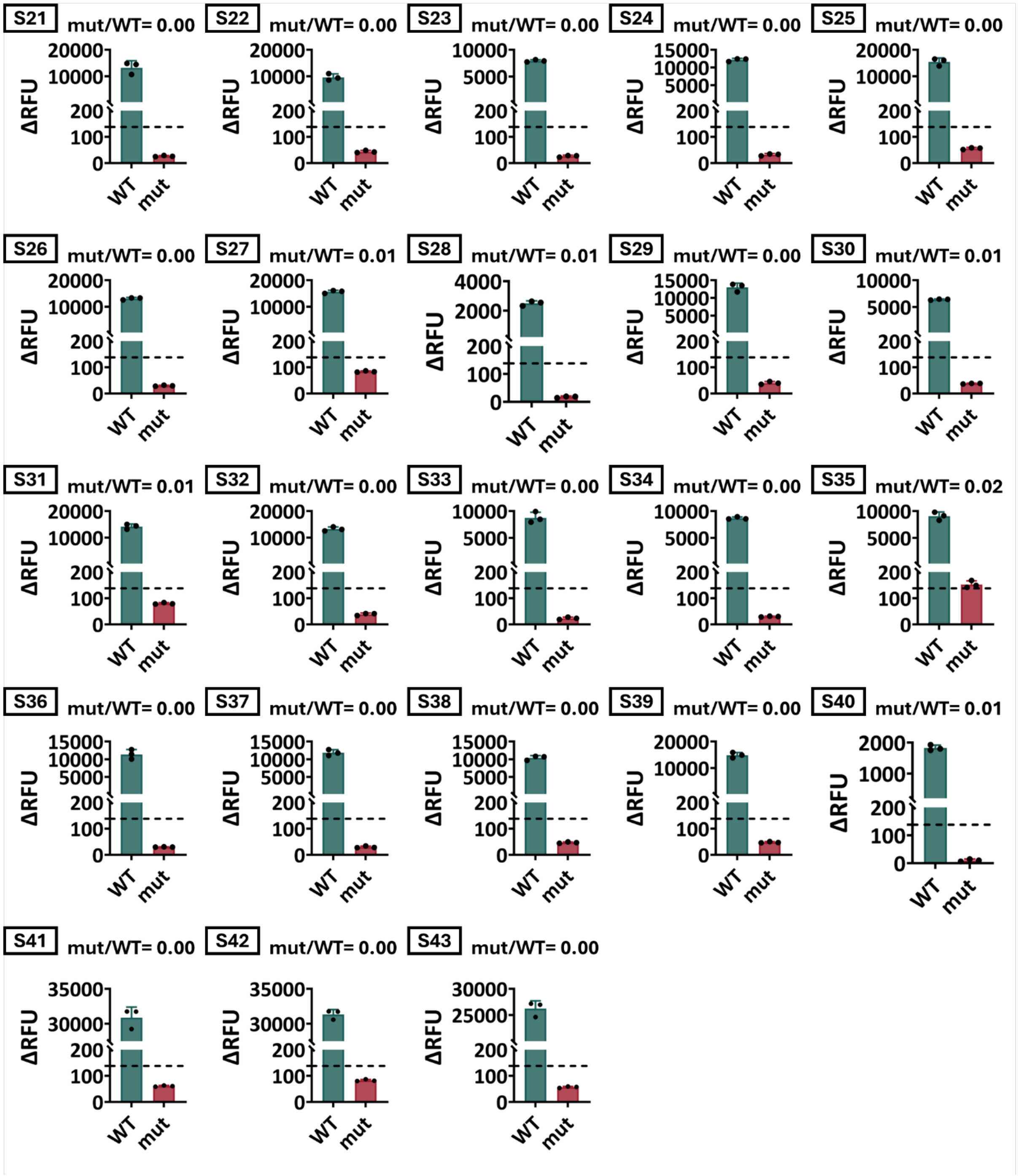

Supplementary Figure 31. Individual ΔRFU responses and mutant-to-wild-type ratios for IDH1 wild-type samples. Bar plots show ΔRFU signals for each of the 23 IHC-verified IDH1 wild-type FFPE glioma samples (S21–S43), with the corresponding mutant-to-wild-type (mut/WT) ratios indicated above each panel. Each bar represents the mean ± s.d. of three technical replicates. The dashed line marks the cutoff value (138), defined as the mean + 3×s.d. of wild-type samples.

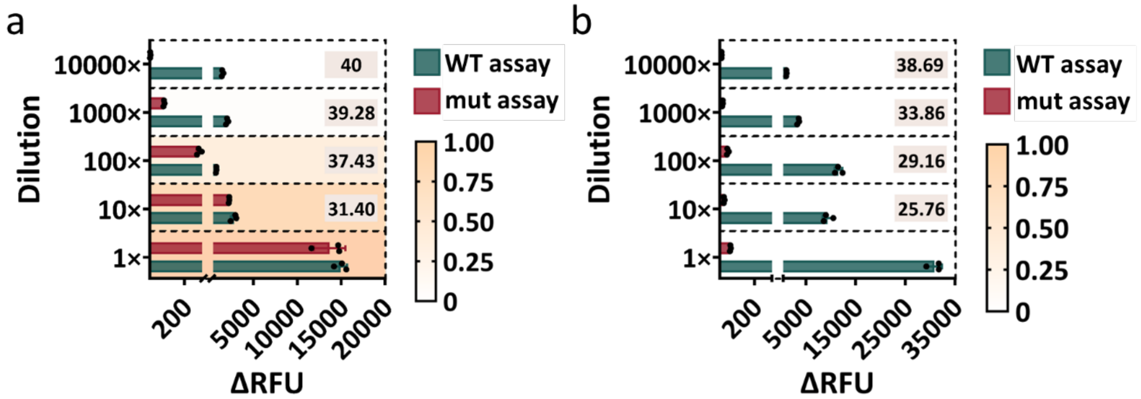

Supplementary Figure 32. Dilution analysis of FFPE glioma specimens by SDS-CRISPR and qPCR. (a) Serial dilutions of an IHC-confirmed IDH1R132H-positive FFPE sample (S7) tested in parallel by SDS-CRISPR assays and qPCR. For qPCR, each dilution was run in triplicate, with undetermined Ct values set to 40; numbers denote mean Ct values across replicates. Background shading indicates the mutant-to-wild-type ratio derived from SDS-CRISPR fluorescence. (b) Serial dilutions of an IHC-confirmed wild-type FFPE sample (S41) tested in parallel by SDS-CRISPR and qPCR. Ct values are presented as in (a), and shading reflects the mutant-to-wild-type ratio from SDS-CRISPR fluorescence.

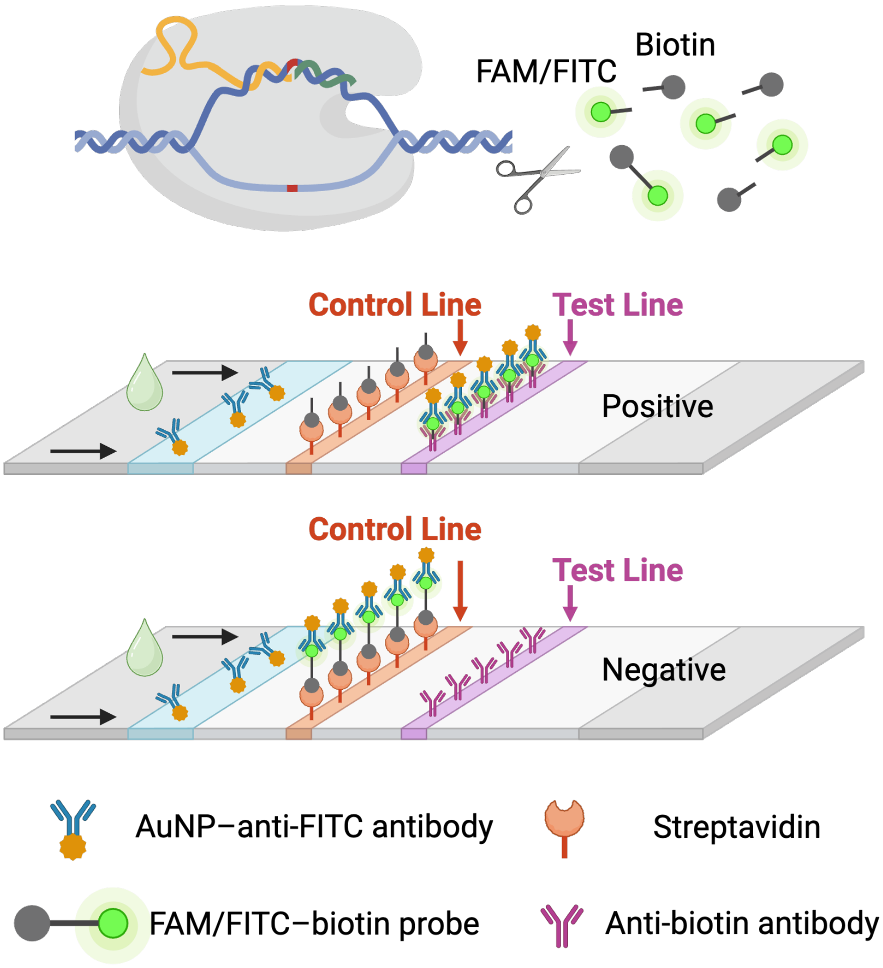

Supplementary Figure 33. Schematic illustration of the SDS-CRISPR lateral-flow detection principle[2]. Target recognition activates Cas12a *trans*-cleavage, resulting in cleavage of the probe. The cleaved probe fragments generate a visible signal at the test line. In the absence of target, the probe remains intact and is captured only at the control line, producing a negative result.

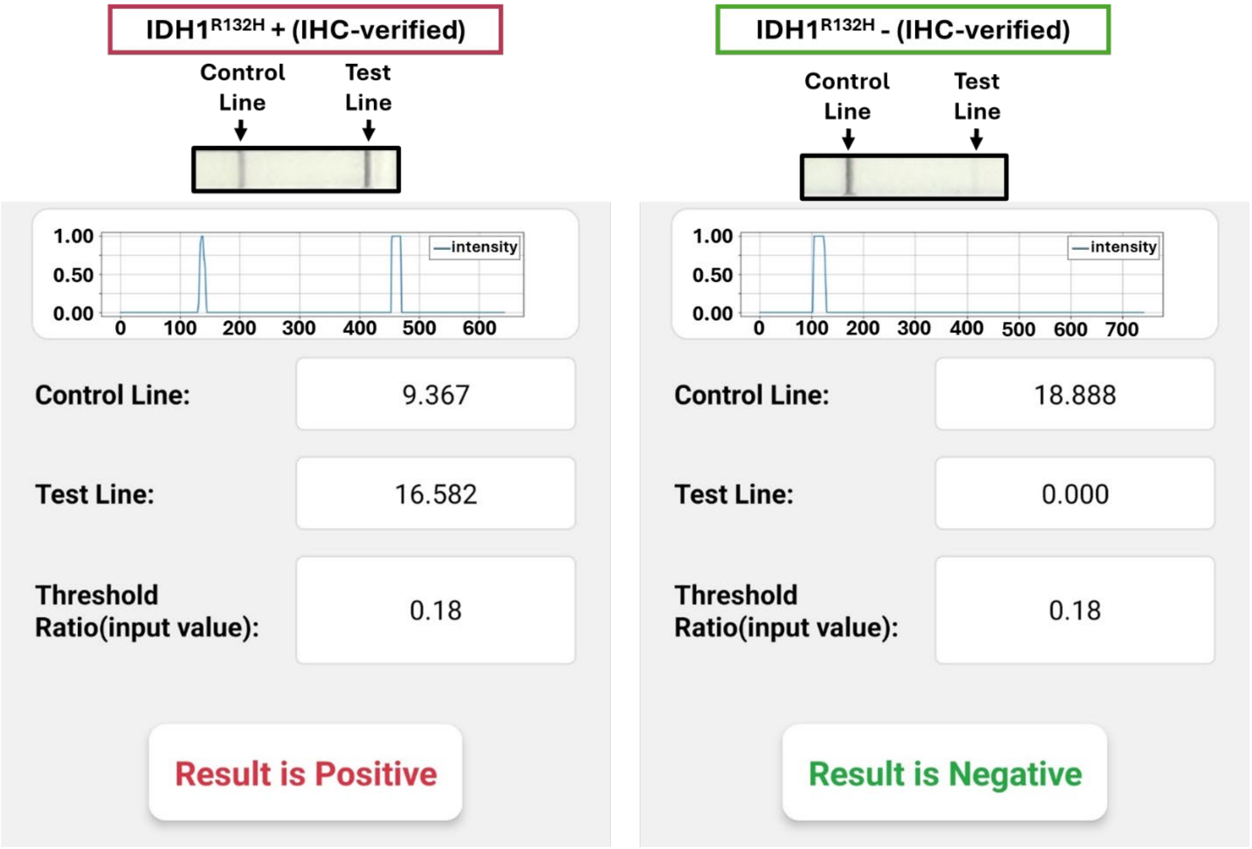

Supplementary Figure 34. Representative IHC-verified IDH1^{R132H}-positive (S7) and -negative (S41) FFPE samples analyzed by lateral-flow strips with smartphone-based quantification and classification.

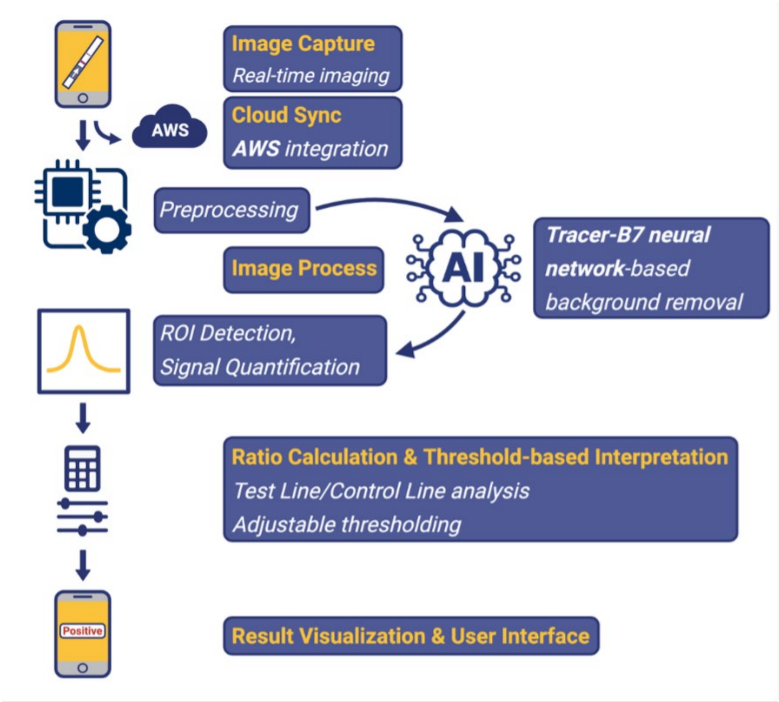

Supplementary Figure 35. Workflow of the mobile application for assay interpretation. The application enables real-time image capture of test strips with optional cloud synchronization through AWS. Captured images are preprocessed and analyzed using AI-powered modules (Carvekit for background removal) and OpenCV-based algorithms for region-of-interest (ROI) detection and signal quantification. This pipeline allows objective and robust evaluation of test and control lines, independent of user expertise or ambient lighting. Ratio calculation and threshold-based interpretation are then performed, and results are displayed through a user-friendly mobile interface.

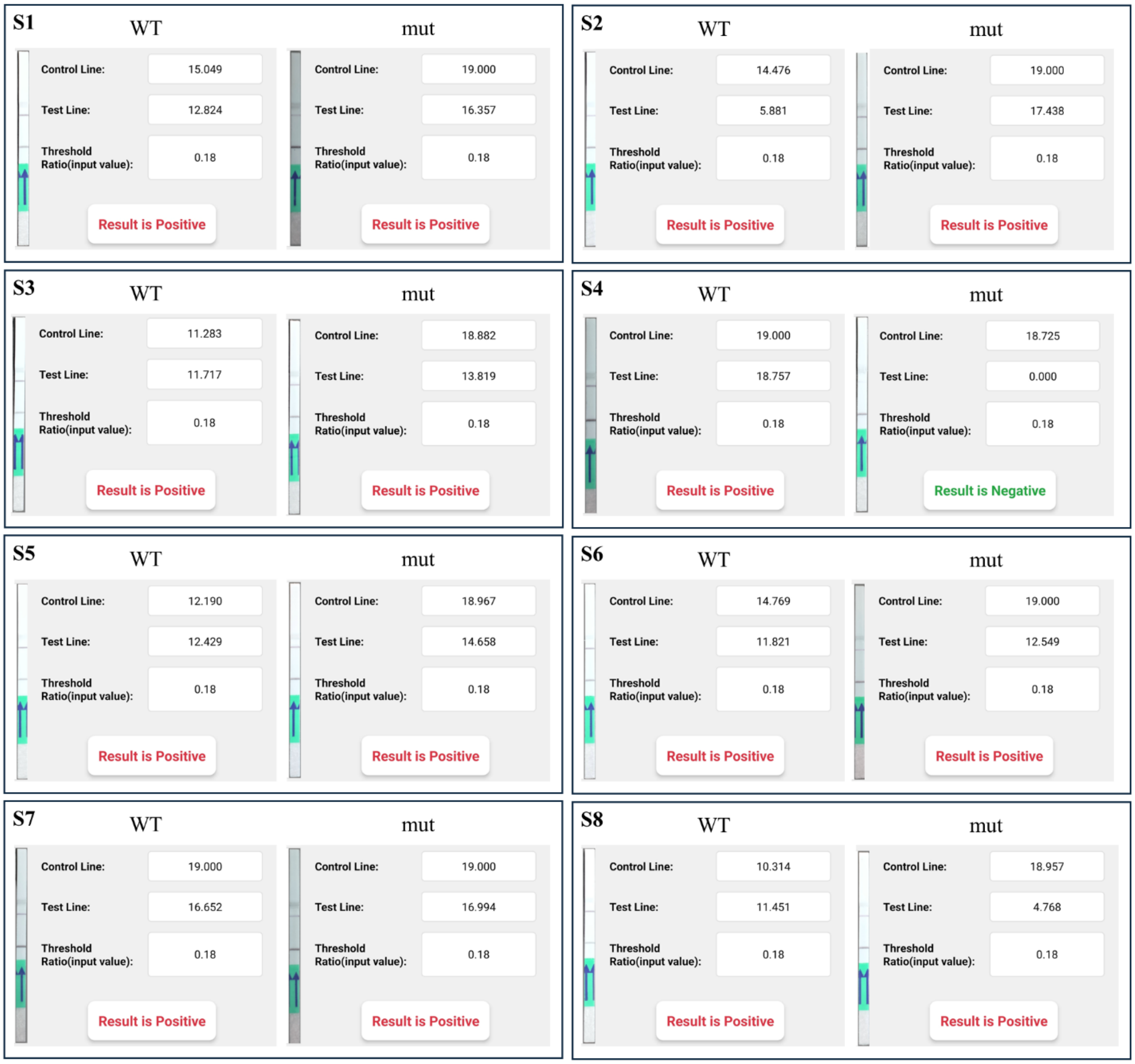

Supplementary Figure 36. Smartphone-assisted lateral-flow readouts for SDS-CRISPR assays on FFPE glioma specimens. S1–S8 were tested using wild-type (WT) and mutant (mut) SDS-CRISPR assays. For each assay, the corresponding lateral-flow strip image and smartphone app output are shown (continued).

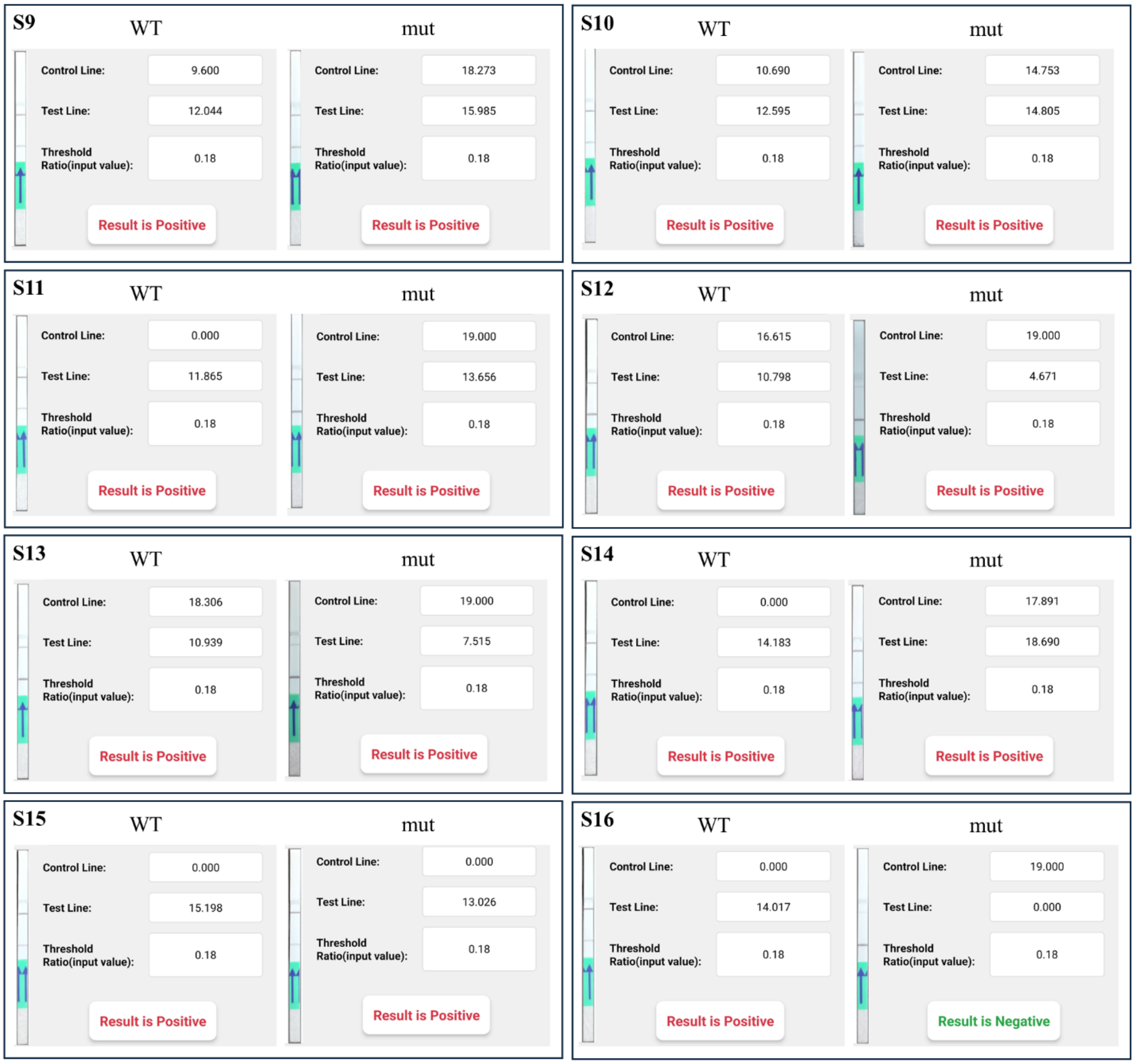

Supplementary Figure 37. S9–S16 (continued from Supplementary Figure 36).

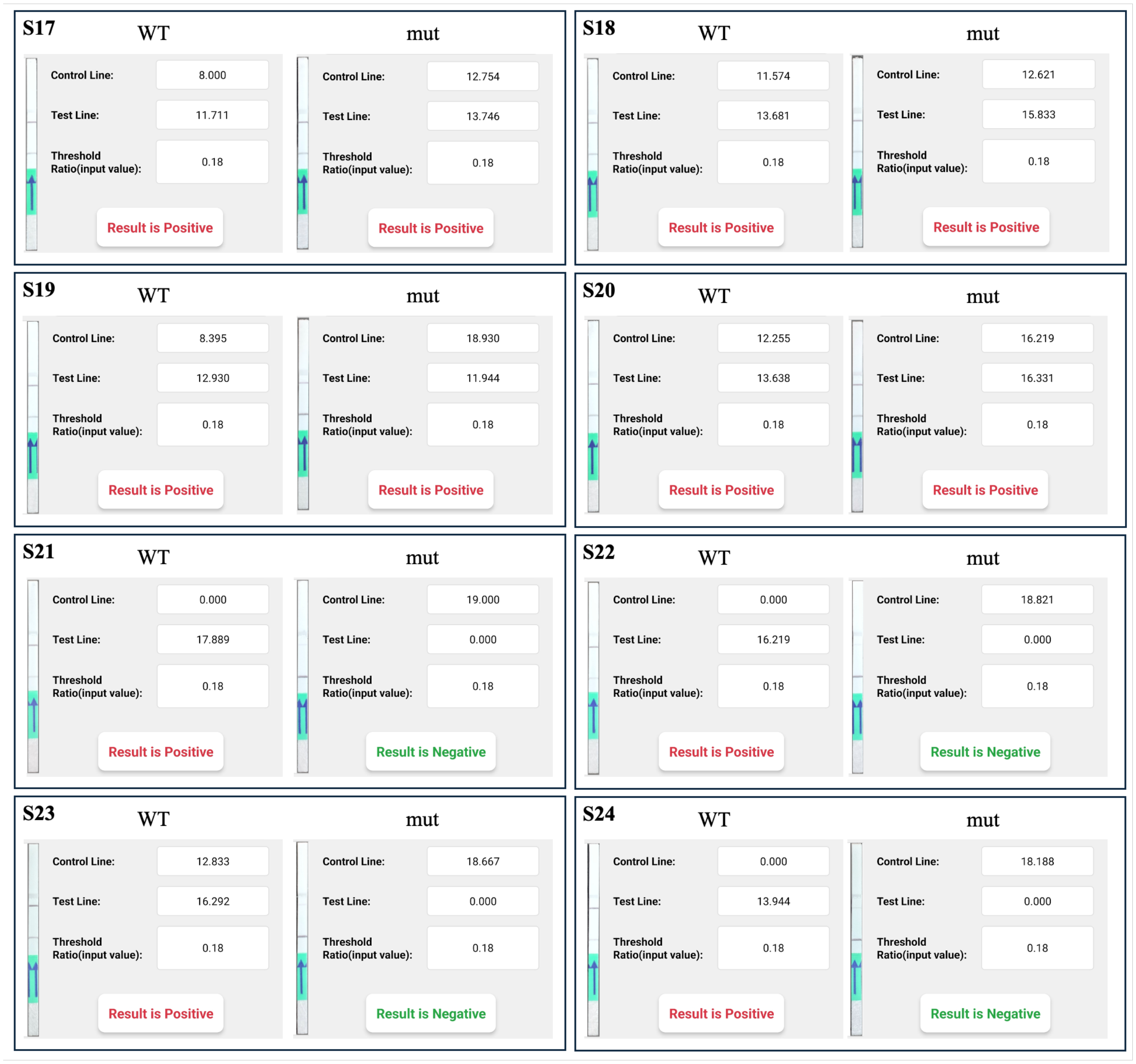

Supplementary Figure 38. S17–S24 (continued from Supplementary Figure 36).

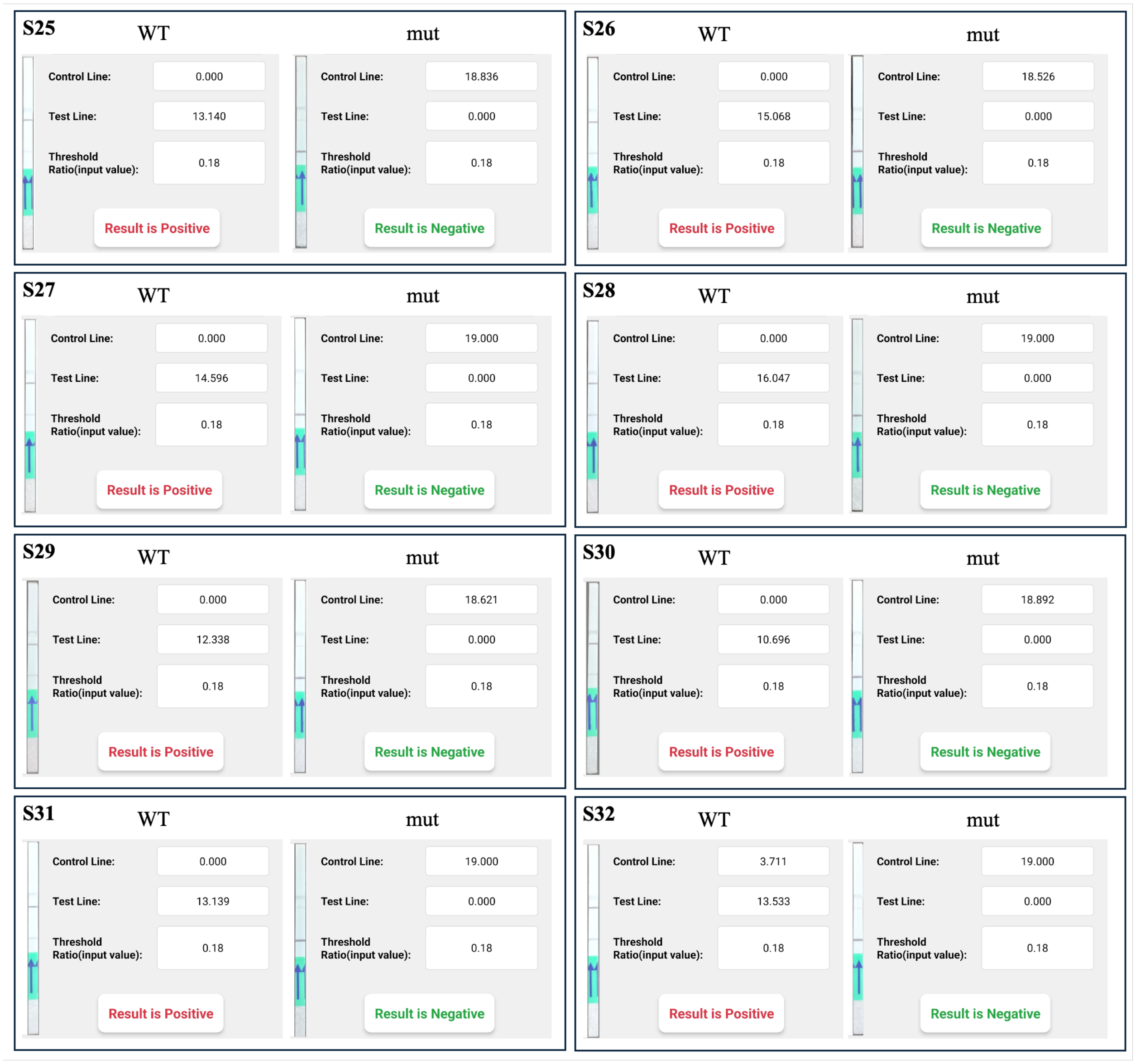

Supplementary Figure 39. S25–S32 (continued from Supplementary Figure 36).

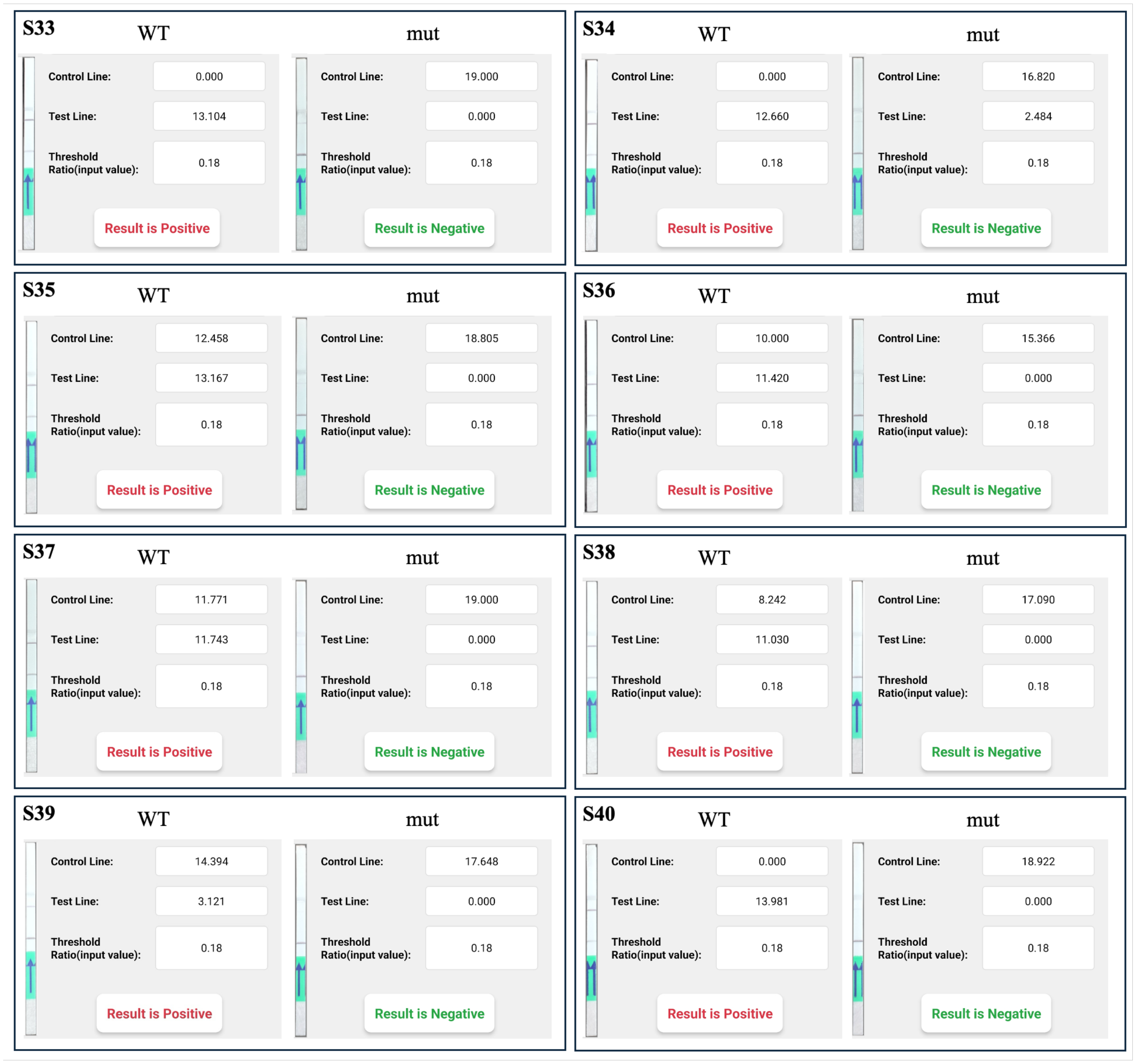

Supplementary Figure 40. S33–S40 (continued from Supplementary Figure 36).

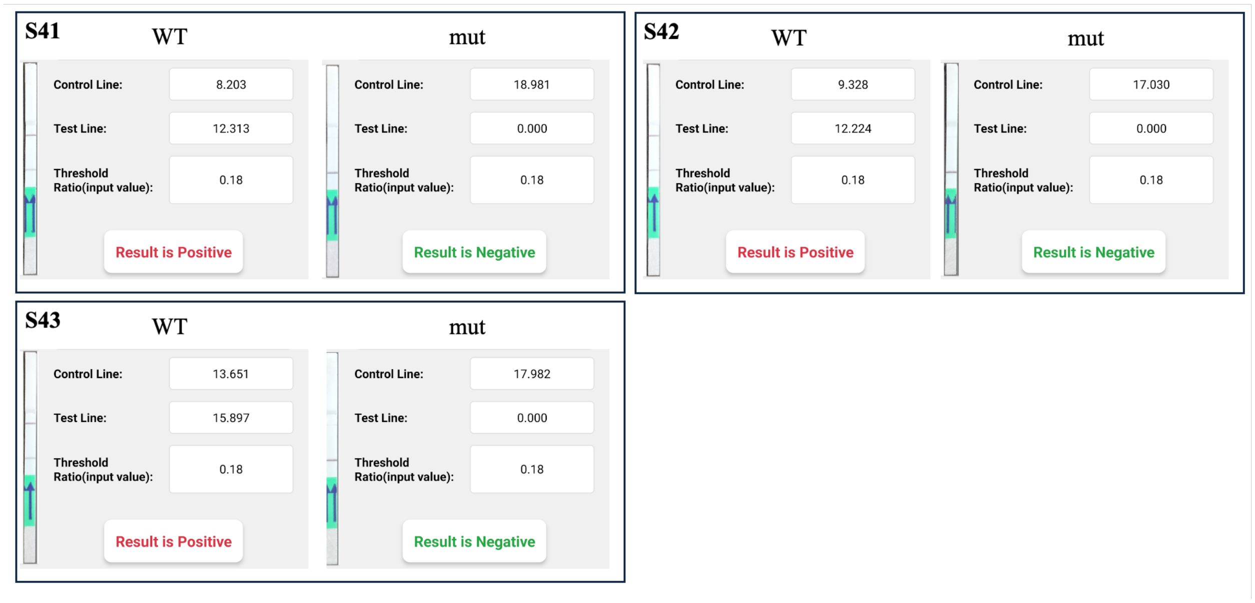

Supplementary Figure 41. S41–S43 (continued from Supplementary Figure 36).

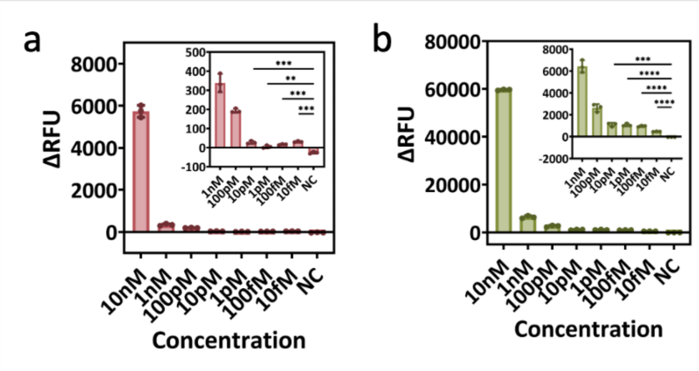

Supplementary Figure 42. Sensitivity of canonical CRISPR assays for Let-7a (a) and miR-98 (b) across target concentrations ranging from 10 nM to 10 fM. Fluorescence signals were measured within 60 min of reaction. Statistical significance was assessed using two-tailed unpaired t-tests (**P < 0.01; ***P < 0.001; ****P < 0.0001), comparing each concentration to the no-target control (NC). Exact P values are provided in Supplementary Tables 16–17.

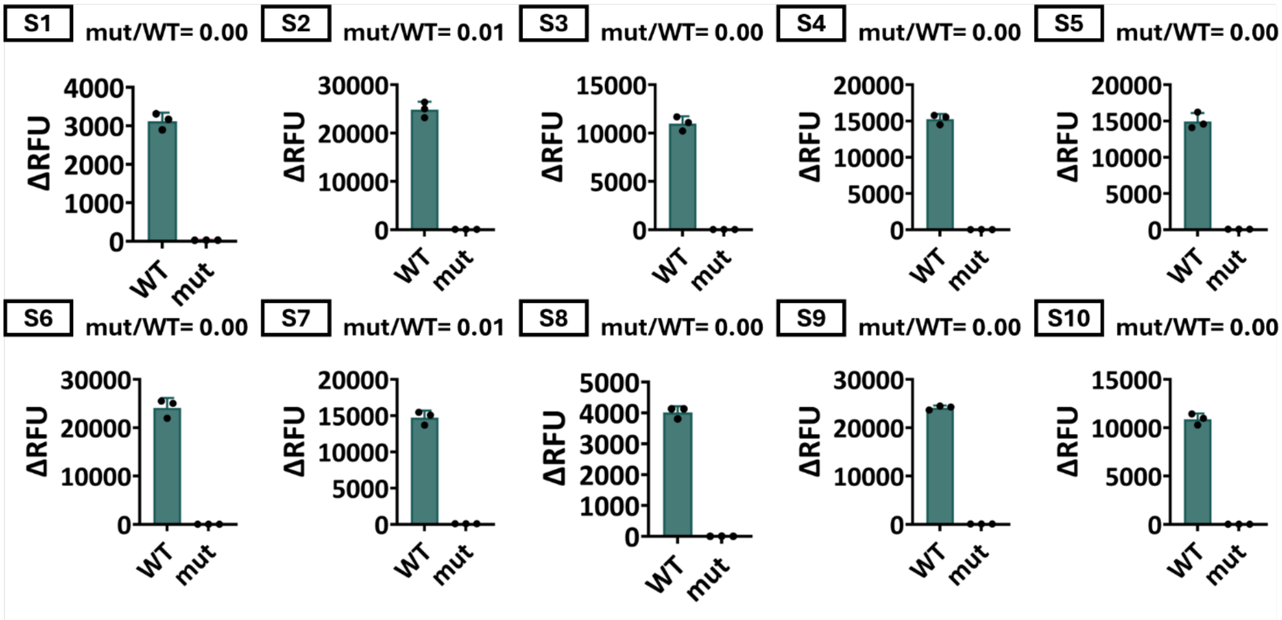

Supplementary Figure 43. SDS-CRISPR detection in fresh-frozen non-glioma controls. Bar plots show ΔRFU signals from ten fresh-frozen non-glioma cancer samples (S1–S10). Each bar represents the mean ± s.d. of three technical replicates, with the corresponding mutant-to-wild-type (mut/WT) ratios shown above each panel.

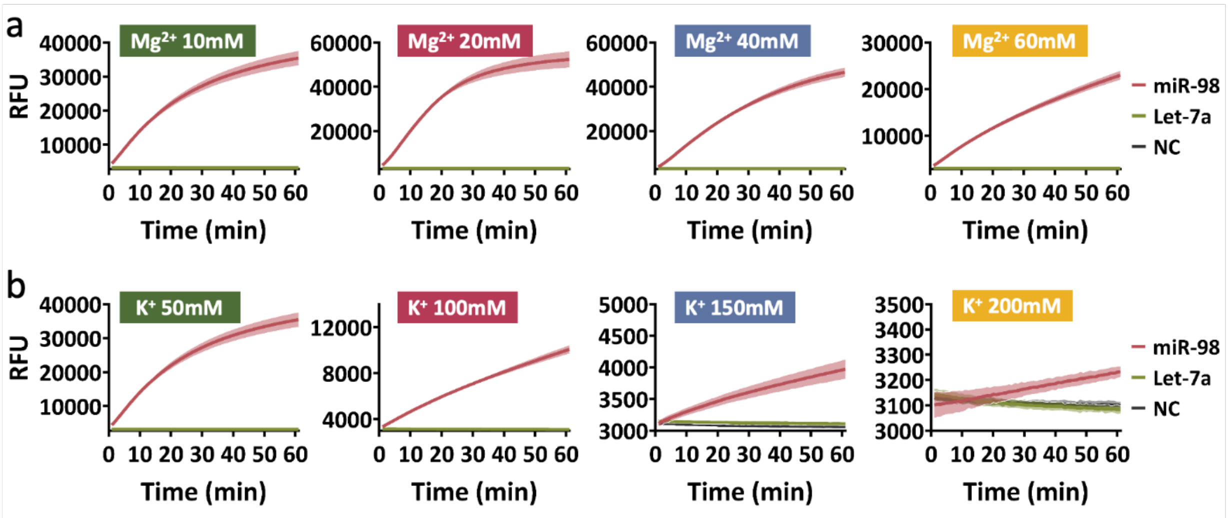

Supplementary Figure 44. Real-time fluorescence kinetics of Cas12a collateral cleavage in the SDS-CRISPR miR-98 assay at varying ionic concentrations. (a) Mg^{2+} concentrations (10, 20, 40, and 60 mM). (b) K^{+} concentrations (50, 100, 150, and 200 mM).

Supplementary Table 1. Two-tailed unpaired t-tests for mut vs. PC at each SNV position under the combined *crSplit-Type I* and *actSplit-Pp* design. This table provides the statistical values corresponding to Figure 4b. Pos., position; Diff., difference; SE, standard error.

Pos.	mut Type	P-value	Mean (PC)	Mean (mut)	Diff.	SE of Diff.	t ratio	df
B1	mut1	0.01539	2,035	1,335	700	172	4.06	4
B1	mut2	0.01037	2,035	1,293	742	163	4.56	4
B1	mut3	0.01149	2,035	1,266	769	174	4.42	4
B2	mut1	0.14345	1,666	1,469	197	108	1.82	4
B2	mut2	0.15287	1,666	1,366	299	170	1.76	4
B2	mut3	0.05441	1,666	1,303	363	135	2.69	4
B3	mut1	0.01806	2,525	1,392	1,133	293	3.87	4
B3	mut2	0.01163	2,525	1,346	1,179	268	4.41	4
B3	mut3	0.00555	2,525	1,054	1,471	270	5.44	4
B4	mut1	0.00002	1,076	179	897	39.7	22.6	4
B4	mut2	0.00002	1,076	166	910	37.3	24.4	4
B4	mut3	0.00002	1,076	117	959	39.2	24.5	4
B5	mut1	0.92777	50.3	49.8	0.414	4.29	0.0965	4
B5	mut2	0.00056	50.3	9.19	41.1	4.11	9.99	4
B5	mut3	0.00051	50.3	8.43	41.8	4.08	10.3	4
B6	mut1	0.00043	18	1.85	16.2	1.51	10.7	4
B6	mut2	0.00052	18	1.68	16.3	1.6	10.2	4
B6	mut3	0.00014	18	0.376	17.7	1.23	14.4	4
B7	mut1	0.07446	-0.381	2.69	-3.07	1.28	2.4	4
B7	mut2	0.33404	-0.381	2.45	-2.83	2.58	1.1	4
B7	mut3	0.37084	-0.381	-1.59	1.21	1.2	1.01	4
B8	mut1	0.20621	-0.638	1.81	-2.45	1.62	1.51	4
B8	mut2	0.53128	-0.638	-0.942	0.303	0.443	0.684	4
B8	mut3	0.17528	-0.638	-2.32	1.68	1.02	1.65	4
B9	mut1	0.03624	-2.61	-0.245	-2.37	0.763	3.1	4
B9	mut2	0.13157	-2.61	-1.14	-1.47	0.777	1.89	4
B9	mut3	0.38936	-2.61	-1.82	-0.792	0.821	0.965	4
B10	mut1	0.90355	0.558	0.326	0.232	1.79	0.129	4
B10	mut2	0.56687	0.558	-0.529	1.09	1.74	0.623	4
B10	mut3	0.51161	0.558	-0.676	1.23	1.71	0.72	4
B11	mut1	0.00341	-0.79	1.63	-2.42	0.389	6.21	4
B11	mut2	0.30987	-0.79	-0.0535	-0.737	0.634	1.16	4
B11	mut3	0.64050	-0.79	-0.926	0.135	0.268	0.504	4
B12	mut1	0.06078	0.0165	3.17	-3.15	1.22	2.59	4
B12	mut2	0.20985	0.0165	2.99	-2.97	1.99	1.49	4
B12	mut3	0.32658	0.0165	2.87	-2.86	2.56	1.12	4
B13	mut1	0.54768	5.85	7.63	-1.78	2.71	0.656	4

B13	mut2	0.85555	5.85	5.32	0.537	2.76	0.194	4
B13	mut3	0.19186	5.85	1.64	4.22	2.69	1.57	4
B14	mut1	0.91274	3.12	3.01	0.117	1	0.117	4
B14	mut2	0.91844	3.12	2.99	0.136	1.25	0.109	4
B14	mut3	0.30483	3.12	1.51	1.61	1.37	1.18	4
B15	mut1	0.19290	0.135	2.62	-2.49	1.59	1.56	4
B15	mut2	0.96144	0.135	0.174	-0.0399	0.776	0.0514	4
B15	mut3	0.89634	0.135	0.0224	0.112	0.808	0.139	4
B16	mut1	0.86739	1.91	1.55	0.357	2.01	0.178	4
B16	mut2	0.65755	1.91	0.0201	1.89	3.95	0.478	4
B16	mut3	0.29228	1.91	-0.909	2.82	2.32	1.21	4
B17	mut1	0.00120	141	5.87	135	16.4	8.21	4
B17	mut2	0.00111	141	2.84	138	16.4	8.37	4
B17	mut3	0.00099	141	-1.28	142	16.4	8.64	4
B18	mut1	0.00206	416	107	309	43.4	7.11	4
B18	mut2	0.00168	416	89	327	43.5	7.52	4
B18	mut3	0.00146	416	75.1	341	43.8	7.79	4
B19	mut1	0.03036	936	426	509	155	3.29	4
B19	mut2	0.01551	936	390	546	135	4.05	4
B19	mut3	0.01053	936	320	615	136	4.54	4

Supplementary Table 2. Two-tailed unpaired t-tests for mut vs. PC at each SNV position under the combined *crSplit-Type II* and *actSplit-Pp* design. This table provides the statistical values corresponding to Figure 4c. Pos., position; Diff., difference; SE, standard error.

Pos.	mut Type	P-value	Mean (PC)	Mean (mut)	Diff.	SE of diff.	t ratio	df
B1	mut1	0.00134	958	226	732	91.7	7.98	4
B1	mut2	0.00104	958	183	775	91	8.52	4
B1	mut3	0.00085	958	162	796	88.7	8.98	4
B2	mut1	0.00470	308	216	92.2	16.2	5.69	4
B2	mut2	0.00258	308	190	118	17.7	6.7	4
B2	mut3	0.00008	308	187	121	7.33	16.5	4
B3	mut1	0.00002	1,391	214	1,178	46.9	25.1	4
B3	mut2	0.00001	1,391	125	1,267	44.8	28.3	4
B3	mut3	0.00001	1,391	105	1,286	44.5	28.9	4
B4	mut1	0.00005	3,397	190	3,207	176	18.2	4
B4	mut2	0.00005	3,397	186	3,211	176	18.3	4
B4	mut3	0.00005	3,397	174	3,223	176	18.3	4
B5	mut1	0.00013	3,250	259	2,991	203	14.7	4
B5	mut2	0.00012	3,250	219	3,031	203	14.9	4
B5	mut3	0.00011	3,250	148	3,102	204	15.2	4
B6	mut1	0.00023	6,842	255	6,587	521	12.6	4
B6	mut2	0.00020	6,842	18.4	6,824	521	13.1	4
B6	mut3	0.00020	6,842	17.9	6,824	521	13.1	4
B7	mut1	0.00154	8.2	2.92	5.28	0.687	7.68	4
B7	mut2	0.00074	8.2	2.77	5.43	0.583	9.32	4
B7	mut3	0.00078	8.2	2.2	6	0.652	9.2	4
B8	mut1	0.00011	71.9	17	54.9	3.63	15.1	4
B8	mut2	0.00010	71.9	14.9	57	3.66	15.6	4
B8	mut3	0.00008	71.9	13.7	58.3	3.58	16.3	4
B9	mut1	0.00019	1,307	63.9	1,243	94.4	13.2	4
B9	mut2	0.00016	1,307	7.78	1,299	94.4	13.8	4
B9	mut3	0.00016	1,307	7.09	1,300	94.4	13.8	4
B10	mut1	0.00026	1,412	36.3	1,376	113	12.2	4
B10	mut2	0.00025	1,412	17.3	1,395	113	12.3	4
B10	mut3	0.00024	1,412	5.81	1,406	113	12.4	4
B11	mut1	0.00010	6,976	207	6,769	434	15.6	4
B11	mut2	0.00009	6,976	61.5	6,914	434	15.9	4
B11	mut3	0.00009	6,976	50.9	6,925	434	16	4
B12	mut1	0.00001	3,043	434	2,609	90.7	28.8	4
B12	mut2	0.00001	3,043	130	2,913	90.2	32.3	4
B12	mut3	0.00001	3,043	23.1	3,020	91	33.2	4
B13	mut1	0.00011	510	21.4	488	32.1	15.2	4

B13	mut2	0.00010	510	17.4	492	32	15.4	4
B13	mut3	0.00009	510	1.89	508	32	15.9	4
B14	mut1	0.00002	1,405	83.8	1,321	54.1	24.4	4
B14	mut2	0.00002	1,405	70.9	1,334	53.8	24.8	4
B14	mut3	0.00002	1,405	66.1	1,339	53.7	24.9	4
B15	mut1	0.00004	1,158	73.7	1,084	53.8	20.1	4
B15	mut2	0.00003	1,158	49.1	1,109	53.5	20.7	4
B15	mut3	0.00003	1,158	47.6	1,110	53.5	20.7	4
B16	mut1	0.00048	3,183	188	2,995	288	10.4	4
B16	mut2	0.00038	3,183	3.43	3,179	288	11.1	4
B16	mut3	0.00038	3,183	2.1	3,181	288	11.1	4
B17	mut1	0.00133	22.3	38.4	-16.1	2.01	8	4
B17	mut2	0.00027	22.3	8.18	14.1	1.16	12.1	4
B17	mut3	0.00023	22.3	1.02	21.3	1.7	12.5	4
B18	mut1	0.00085	81.4	48.1	33.4	3.72	8.98	4
B18	mut2	0.00070	81.4	34.7	46.7	4.95	9.44	4
B18	mut3	0.00011	81.4	23.4	58	3.8	15.3	4
B19	mut1	0.00161	324	137	187	24.6	7.59	4
B19	mut2	0.00087	324	122	202	22.6	8.94	4
B19	mut3	0.00043	324	82	242	22.6	10.7	4

Supplementary Table 3. Two-tailed Welch's t-test comparing PC and NC across SNV positions. Unpaired two-tailed Welch's t-tests were performed to compare perfect-match controls (PC) and no-template controls (NC) at each SNV position. This table provides the statistical values corresponding to Figure 4d-*Type I*. Diff., difference; SE, standard error.

	**n (PC, NC)**	**Mean (PC)**	**Mean (NC)**	**Diff. ± SE**	**Welch's t (df)**	**P-value**
B1	3, 3	1.00	1.3e-6	-1.000 ± 0.315	t (3.52) = 3.18	0.0402
B2	3, 3	1.00	6.7e-7	-1.000 ± 0.4249	t (3.82) = 2.35	0.0813
B3	3, 3	1.00	3.3e-7	-1.000 ± 0.2555	t (2.22) = 3.91	0.0501
B4	3, 3	1.00	-1.3e-6	-1.000 ± 0.03826	t (2.08) = 26.14	0.0012
B6	3, 3	1.00	1.0e-6	-1.000 ± 0.07855	t (2.862) = 12.73	0.0013
B17	3, 3	1.00	-6.7e-7	-1.000 ± 0.1159	t (2.002) = 8.627	0.0131
B18	3, 3	1.00	-1.0e-6	-1.000 ± 0.1035	t (2.000) = 9.666	0.0105
B19	3, 3	1.00	-3.0e-6	-1.000 ± 0.1430	t (2.000) = 6.992	0.0198

Supplementary Table 4. Two-tailed exact Mann–Whitney U tests comparing PC and mut across SNV positions. Comparisons between PC (n = 3) and pooled mut groups (mut1–mut3 combined, n = 9) were evaluated using two-tailed exact Mann–Whitney U tests. This table provides the statistical values corresponding to Figure 4d-*Type I*. Reported values include group medians, Hodges–Lehmann median differences, U statistics, and P-values. Diff., difference.

	**Median of PC**	**Median of mut**	**Diff.: Actual**	**Diff.: Hodges–Lehmann**	**Mann–Whitney U**	**P-value**
B1	0.9355, n=3	-0.1732, n=9	-1.109	-1.109	0	0.0091
B2	1.122, n=3	0.2403, n=9	-0.8812	-0.9181	2	0.0364
B3	1.133, n=3	-0.2229, n=9	-1.356	-1.265	0	0.0091
B4	1.019, n=3	0.07170, n=9	-0.9477	-0.9441	0	0.0091
B5	1.063, n=3	0.1936, n=9	-0.8696	-0.6687	3	0.0636
B6	1.053, n=3	-0.008440, n=9	-1.061	-0.9815	0	0.0091
B7	1.074, n=3	0.005499, n=9	-1.068	-1.068	9	0.4818
B8	1.153, n=3	1.429, n=9	0.2755	0.2755	12	0.8636
B9	-0.9134, n=3	-0.7579, n=9	0.1555	0.2099	4	0.1
B10	1.783, n=3	-0.03174, n=9	-1.815	-1.815	9	0.4818
B11	0.9281, n=3	0.8089, n=9	-0.1191	-0.466	8	0.3727
B12	1.664, n=3	6.257, n=9	4.593	4.774	5	0.1455
B13	0.7693, n=3	0.8680, n=9	0.0987	-0.1121	12	0.8636
B14	0.7875, n=3	0.3227, n=9	-0.4649	-0.5374	12	0.8636
B15	-0.4644, n=3	0.6010, n=9	1.065	0.1376	13	>0.9999
B16	0.7269, n=3	0.5836, n=9	-0.1433	-0.2569	11	0.7273
B17	1.089, n=3	0.02190, n=9	-1.067	-1.067	0	0.0091
B18	1.082, n=3	0.2159, n=9	-0.8664	-0.8484	0	0.0091
B19	1.108, n=3	0.3832, n=9	-0.7247	-0.6698	0	0.0091

Supplementary Table 5. Two-tailed Welch's t-test comparing PC and NC across SNV positions. Unpaired two-tailed Welch's t-tests were performed to compare perfect-match controls (PC) and no-template controls (NC) at each SNV position. This table provides the statistical values corresponding to Figure 4d-*Type II*. Diff. ± SE, difference ± standard error.

	**n (PC, NC)**	**Mean (PC)**	**Mean (NC)**	**Diff. ± SE**	**Welch's t (df)**	**P-value**
B1	3, 3	1.00	3.3e-7	-1.000 ± 0.1155	t (2.150) = 8.655	0.0105
B3	3, 3	1.00	6.7e-7	-1.000 ± 0.03393	t (2.041) = 29.47	0.0010
B4	3, 3	1.00	1.3e-6	-1.000 ± 0.05458	t (2.004) = 18.32	0.0029
B5	3, 3	1.00	-6.7e-7	-1.000 ± 0.06437	t (2.000) = 15.53	0.0041
B6	3, 3	1.00	0.000	-1.000 ± 0.07642	t (2.000) = 13.09	0.0058
B7	3, 3	1.00	6.7e-7	-1.000 ± 0.1359	t (3.659) = 7.356	0.0026
B8	3, 3	1.00	-6.7e-7	-1.000 ± 0.04857	t (2.021) = 20.59	0.0022
B9	3, 3	1.00	3.3e-7	-1.000 ± 0.07215	t (2.000) = 13.86	0.0052
B10	3, 3	1.00	-1.0e-6	-1.000 ± 0.07999	t (2.000) = 12.50	0.0063
B11	3, 3	1.00	3.3e-7	-1.000 ± 0.06223	t (2.000) = 16.07	0.0039
B12	3, 3	1.00	-6.7e-7	-1.000 ± 0.02955	t (2.000) = 33.84	0.0009
B13	3, 3	1.00	1.3e-6	-1.000 ± 0.06260	t (2.001) = 15.97	0.0039
B14	3, 3	1.00	-1.3e-6	-1.000 ± 0.03915	t (2.007) = 25.54	0.0015
B15	3, 3	1.00	1.0e-6	-1.000 ± 0.04643	t (2.001) = 21.54	0.0021
B16	3, 3	1.00	9.7e-7	-1.000 ± 0.09039	t (2.000) = 11.06	0.0081
B18	3, 3	1.00	-6.7e-7	-1.000 ± 0.04968	t (2.959) = 20.13	0.0003
B19	3, 3	1.00	-1.0e-6	-1.000 ± 0.06895	t (2.005) = 14.50	0.0047

Supplementary Table 6. Two-tailed exact Mann–Whitney U tests comparing PC and mut across SNV positions. Comparisons between PC (n = 3) and pooled Mut groups (mut1–mut3 combined, n = 9) were evaluated using two-tailed exact Mann–Whitney U tests. This table provides the statistical values corresponding to Figure 4d-*Type II*. Reported values include group medians, Hodges–Lehmann median differences, U statistics, and P-values. Diff., difference.

	**Median of PC**	**Median of mut**	**Diff.: Actual**	**Diff.: Hodges–Lehmann**	**Mann–Whitney U**	**P-value**
B1	1.006, n=3	0.0002690, n=9	-1.006	-1.006	0	0.0091
B2	1.437, n=3	0.3945, n=9	-1.043	-0.9258	8	0.3727
B3	0.9703, n=3	0.03852, n=9	-0.9318	-0.9439	0	0.0091
B4	1.054, n=3	0.0007430, n=9	-1.053	-1.05	0	0.0091
B5	0.9580, n=3	0.04232, n=9	-0.9157	-0.9157	0	0.0091
B6	1.007, n=3	-0.0005500, n=9	-1.008	-1.008	0	0.0091
B7	1.000, n=3	0.1674, n=9	-0.8327	-0.8327	0	0.0091
B8	0.9567, n=3	0.2308, n=9	-0.7259	-0.7413	0	0.0091
B9	1.014, n=3	0.007115, n=9	-1.007	-1.007	0	0.0091
B10	1.073, n=3	0.01277, n=9	-1.06	-1.059	0	0.0091
B11	0.9944, n=3	0.009331, n=9	-0.9851	-0.9851	0	0.0091
B12	1.026, n=3	0.04367, n=9	-0.9819	-0.9368	0	0.0091
B13	1.020, n=3	0.03593, n=9	-0.9844	-0.9844	0	0.0091
B14	0.9853, n=3	0.02675, n=9	-0.9586	-0.9586	0	0.0091
B15	1.027, n=3	0.03937, n=9	-0.9877	-0.9877	0	0.0091
B16	0.9641, n=3	0.0005220, n=9	-0.9635	-0.9635	0	0.0091
B17	1.003, n=3	0.4294, n=9	-0.5737	-0.5737	9	0.4818
B18	0.9886, n=3	0.4559, n=9	-0.5327	-0.5905	0	0.0091
B19	1.054, n=3	0.3664, n=9	-0.6879	-0.6653	0	0.0091

Supplementary Table 7. Michaelis–Menten kinetic parameters for canonical and SDS-CRISPR assays. Best-fit values of V_{max} and K_m were determined by nonlinear regression for both canonical and SDS-CRISPR assays.

	**Canonical CRISPR Assay**	**SDS-CRISPR Assay**
**Michaelis–Menten**		
**Best-fit values**		
**Vmax**	2.025	1.024
**Km**	890.9	686.4
**95% CI (profile likelihood)**		
**Vmax**	1.740 to 2.431	0.9197 to 1.158
**Km**	680.5 to 1206	558.3 to 856.7
**Goodness of Fit**		
**Degrees of Freedom**	16	16
**R squared**	0.9880	0.9910
**Sum of Squares**	0.02840	0.006877
**Sy.x**	0.04213	0.02073
**Constraints**		
**Km**	Km > 0	Km > 0
**Number of points**		
**# of X values**	18	18
**# Y values analyzed**	18	18

Supplementary Table 8. Measured distances (Å) between Val377 and Gln1136 in Cas12a under different assay conditions.

	**Distance (Å)**
**Positive control**	33.2
**Negative control**	23.1
**WT assay + WT target**	35.3
**WT assay + mut target**	33.1
**mut assay + mut target**	35.3
**mut assay + WT target**	32.3

Supplementary Table 9. Patient demographic and clinical characteristics. Summary of demographic information and relevant clinical features for all patient samples included in this study. N/A indicates data not available for certain cases.

Sample	IDH Status	Histology Diagnosis	Demographics
**FFPE samples**			
**S1**	mutant (IHC verified)	oligodendroglioma	Age 68, Female
**S2**	mutant (IHC verified)	glioblastoma multiforme	Age 61, Male
**S3**	mutant (IHC verified)	oligodendroglioma	Age 59, Female
**S4**	mutant (IHC verified)	diffuse astrocytoma	Age 37, Female
**S5**	mutant (IHC verified)	oligodendroglioma	Age 68, Female
**S6**	mutant (IHC verified)	anaplastic astrocytoma	Age 46, Male
**S7**	mutant (IHC verified)	anaplastic astrocytoma	Age 55, Male
**S8**	mutant (IHC verified)	anaplastic astrocytoma	Age 38, Female
**S9**	mutant (IHC verified)	oligodendroglioma	Age 59, Female
**S10**	mutant (IHC verified)	anaplastic oligodendroglioma	Age 56, Male
**S11**	mutant (IHC verified)	astrocytoma	Age 61, Female
**S12**	mutant (IHC verified)	infiltrating astrocytoma	Age 45, Male
**S13**	mutant (IHC verified)	oligodendroglioma	Age 60, Female
**S14**	mutant (IHC verified)	oligodendroglioma	Age 42, Male
**S15**	mutant (IHC verified)	high grade astrocytoma	Age 20, Male
**S16**	mutant (IHC verified)	oligodendroglioma	Age 53, Female
**S17**	mutant (IHC verified)	high grade astrocytoma	Age 61, Male
**S18**	mutant (IHC verified)	oligodendroglioma	Age 59, Female
**S19**	mutant (IHC verified)	low grade astrocytoma	Age 37, Female
**S20**	mutant (IHC verified)	oligodendroglioma	Age 68, Female
**S21**	wild type (IHC verified)	diffuse astrocytoma	Age 50, Female
**S22**	wild type (IHC verified)	anaplastic astrocytoma	Age 78, Female
**S23**	wild type (IHC verified)	glioblastoma multiforme	Age 63, Female
**S24**	wild type (IHC verified)	anaplastic astrocytoma	Age 78, Female
**S25**	wild type (IHC verified)	glioblastoma	Age 55, Female
**S26**	wild type (IHC verified)	glioblastoma	Age 65, Male
**S27**	wild type (IHC verified)	glioblastoma multiforme	Age 78, Female
**S28**	wild type (IHC verified)	diffuse astrocytoma	Age 74, Male
**S29**	wild type (IHC verified)	glioblastoma multiforme	Age 73, Male
**S30**	wild type (IHC verified)	high grade glioma	Age 31, Male
**S31**	wild type (IHC verified)	glioblastoma multiforme	Age 60, Male
**S32**	wild type (IHC verified)	glioblastoma multiforme	Age 65, Male
**S33**	wild type (IHC verified)	glioblastoma	Age 65, Male
**S34**	wild type (IHC verified)	glioblastoma	Age 65, Male

**S35**	wild type (IHC verified)	glioblastoma	Age 74, Male
**S36**	wild type (IHC verified)	anaplastic astrocytoma	Age 53, Female
**S37**	wild type (IHC verified)	anaplastic astrocytoma	Age 53, Female
**S38**	wild type (IHC verified)	glioblastoma	Age 58, Female
**S39**	wild type (IHC verified)	glioblastoma	Age 63, Male
**S40**	wild type (IHC verified)	pilocytic astrocytoma	Age 22, Female
**S41**	wild type (IHC verified)	meningioma	Age 53, Female
**S42**	wild type (IHC verified)	glioblastoma	Age 77, Male
**S43**	wild type (IHC verified)	glioblastoma	Age 55, Female
**Plasma samples**			
**S1**	wild type	squamous cell carcinoma basaloid, G2	Age 74, Male
**S2**	wild type	ovary with fibroma (benign)	N/A
**S3**	mutant (tissue mutant confirmed by NGS)	adenocarcinoma, invasive	Age 58, Male
**S4**	mutant (tissue mutant confirmed by NGS)	oligodendroglioma	Age 49, Female
**S5**	mutant (tissue mutant confirmed by NGS)	adenocarcinoma, invasive	Age 79, Male
**Fresh Frozen Samples**			
**S1**	wild type	clear cell adenocarcinoma	N/A
**S2**	wild type	serous carcinoma, Stage IIIB1	N/A
**S3**	wild type	high grade endometrial carcinoma	N/A
**S4**	wild type	carcinosarcoma	N/A
**S5**	wild type	endometrioid adenocarcinoma, G2	N/A
**S6**	wild type	endometrial, G1	N/A
**S7**	wild type	endometrial cancer, G1	N/A
**S8**	wild type	endometrioid carcinoma, G2	N/A
**S9**	wild type	squamous cell carcinoma basaloid, G2	N/A
**S10**	wild type	squamous cell carcinoma basaloid, G2	N/A

Supplementary Table 10. Sensitivity analysis of FFPE sample S17 using SDS-CRISPR assays and qPCR (qPCR-pair2).S17 is an IDH1^{R132H}-positive FFPE sample. Serial dilutions were tested in parallel by SDS-CRISPR and qPCR. RFU ratios gradually decreased with increasing dilution but remained above the threshold value of 0.02 (derived from the negative control sample S41) down to the 1:1,000 dilution, demonstrating the assay's sensitivity for detecting low-abundance mutant alleles. Each sample was tested in triplicate. Undetermined reactions without a Ct value were imputed as Ct = 40 for subsequent analysis.

**Dilution**	**Ct Value**	**RFU Ratio**	**ΔRFU of IDH1^{WT}**		**ΔRFU of IDH1^{R132H}**	
			**Mean**	**s.d.**	**Mean**	**s.d.**
1×	-	0.92	14,952.28	710.5585	13,684.45	1,798.642
10×	31.40	0.80	2,774.267	346.5723	2,215.152	36.1847
100×	37.43	0.38	750.4933	34.35368	283.285	13.82364
1,000×	39.28	0.04	1,983.455	127.738	81.45807	3.397747
10,000×	40.0	0.00	1,463.229	90.82532	2.239185	1.656228

Supplementary Table 11. Sensitivity analysis of FFPE sample S41 using SDS-CRISPR and qPCR (qPCR-pair2). S41 is an IDH1 wild-type (negative) FFPE sample. Serial dilutions were tested in parallel by SDS-CRISPR and qPCR. RFU ratios remained near zero across all dilutions, and a threshold value of 0.02 was derived from these negative controls. This threshold was subsequently applied to distinguish positive signals in the dilution series of the mutant-positive sample (S17). Each sample was tested in triplicate. Undetermined reactions without a Ct value were imputed as Ct = 40 for subsequent analysis.

**Dilution**	**Ct Value**	**RFU Ratio**	**ΔRFU of $IDH1^{WT}$**		**ΔRFU of $IDH1^{R132H}$**	
			**Mean**	**s.d.**	**Mean**	**s.d.**
1×	-	0.00	30,908.05	1,444.353	60.8337	2.423613
10×	25.76	0.00	9,348.244	1,016.315	24.94456	2.476445
100×	29.16	0.00	11,543.31	764.632	45.56723	5.484002
1,000×	33.86	0.00	3,457.768	210.0204	15.9901	2.19669
10,000×	38.69	0.01	1,046.015	46.51678	10.39494	0.397735

Supplementary Table 12. NGS analysis of plasma cfDNA from five glioma patients. Summary of sequencing results for the $IDH1^{R132H}$ locus, including reference (ref) and alternate (alt) alleles, read counts, total depth, and calculated variant allele fraction (VAF).

Sample	ref	alt	alt_count	ref_count	depth	VAF
S1	C	T	112	34,767	34,912	0.00
S2	C	T	192	48,190	48,451	0.00
S3	C	T	299	32,998	33,368	0.01
S4	C	T	187	31,731	32,048	0.01
S5	C	T	630	37,648	38,673	0.02

Supplementary Table 13. Plasma cfDNA analysis by SDS-CRISPR. Wild-type and mutant SDS-CRISPR assays were performed on plasma cfDNA from five patients (S1–S5). The table summarizes mean fluorescence signals and standard deviations (s.d.) for wild-type (WT) and mutant (mut) assays, along with the calculated mutant-to-wild-type ratios.

**Sample**	**WT assay**		**mut assay**		**Ratio**
	**Mean**	**s.d.**	**Mean**	**s.d.**	
S1	26,008.76	1,080.68	321.60	30.49	0.01
S2	24,049.17	741.11	479.04	24.72	0.02
S3	22,473.30	1,229.51	1,101.34	65.12	0.05
S4	20,987.99	734.98	897.57	23.89	0.04
S5	16,609.70	425.56	1,211.03	28.60	0.07

Supplementary Table 14. Statistical analysis of SDS-CRISPR assays for Let-7a. Two-tailed unpaired t-tests were performed to compare wild-type and mutant signals at each concentration. Reported values include the difference ± standard error (Diff. ± SE) and 95% confidence intervals (95% CI).

A	B	Mean (A)	Mean (B)	Diff. ± SE	95% CI	P value	t, df
10nM	NC	38,673	-123.8	-38,797 ± 3,196	-47,672 to -29,923	0.0003	t=12.14, df=4
1nM	NC	7,048	-123.8	-7,172 ± 158.4	-7,611 to -6,732	<0.0001	t=45.28, df=4
100pM	NC	2,658	-123.8	-2,782 ± 230.6	-3,422 to -2,142	0.0003	t=12.07, df=4
10pM	NC	1,058	-123.8	-1,182 ± 45.07	-1,307 to -1,057	<0.0001	t=26.22, df=4
1pM	NC	298.1	-123.8	-422.0 ± 39.71	-532.2 to -311.7	0.0004	t=10.63, df=4
100fM	NC	114.1	-123.8	-237.9 ± 18.51	-289.3 to -186.6	0.0002	t=12.86, df=4
10fM	NC	67.12	-123.8	-191.0 ± 20.89	-249.0 to -133.0	0.0008	t=9.140, df=4

Supplementary Table 15. Statistical analysis of SDS-CRISPR assays for miR-98. Two-tailed unpaired t-tests were performed to compare wild-type and mutant signals at each concentration. Reported values include the difference ± standard error (Diff. ± SE) and 95% confidence intervals (95% CI).

**A**	**B**	**Mean (A)**	**Mean (B)**	**Diff. ± SE**	**95% CI**	**P value**	**t, df**
10nM	NC	3911	-19.47	-3,930 ± 70.11	-4,125 to -3,735	<0.0001	t=56.05, df=4
1nM	NC	99.75	-19.47	-119.2 ± 9.783	-146.4 to -92.06	0.0003	t=12.19, df=4
100pM	NC	45.19	-19.47	-64.65 ± 6.536	-82.80 to -46.51	0.0006	t=9.891, df=4
10pM	NC	16.62	-19.47	-36.08 ± 6.791	-54.94 to -17.23	0.006	t=5.314, df=4
1pM	NC	13.72	-19.47	-33.19 ± 6.331	-50.77 to -15.61	0.0063	t=5.242, df=4
100fM	NC	9.257	-19.47	-28.72 ± 10.70	-58.42 to 0.9711	0.0549	t=2.686, df=4
10fM	NC	-0.723	-19.47	-18.74 ± 4.721	-31.85 to -5.636	0.0165	t=3.970, df=4

Supplementary Table 16. Statistical analysis of canonical CRISPR assays for Let-7a. Two-tailed unpaired t-tests were performed to compare wild-type and mutant signals at each concentration. Reported values include the difference ± standard error (Diff. ± SE) and 95% confidence intervals (95% CI).

A	B	Mean (A)	Mean (B)	Diff. ± SE	95% CI	P value	t, df
10nM	NC	5737	-26.52	-5763 ± 164.7	-6221 to -5306	<0.0001	t=34.99, df=4
1nM	NC	337.7	-26.52	-364.2 ± 27.78	-441.3 to -287.0	0.0002	t=13.11, df=4
100pM	NC	193.3	-26.52	-219.8 ± 6.941	-239.1 to -200.5	<0.0001	t=31.67, df=4
10pM	NC	27.48	-26.52	-54.00 ± 5.506	-69.29 to -38.71	0.0006	t=9.808, df=4
1pM	NC	4.917	-26.52	-31.44 ± 5.217	-45.92 to -16.95	0.0038	t=6.026, df=4
100fM	NC	16.32	-26.52	-42.84 ± 3.827	-53.46 to -32.21	0.0004	t=11.19, df=4
10fM	NC	30.88	-26.52	-57.40 ± 3.955	-68.38 to -46.42	0.0001	t=14.51, df=4

Supplementary Table 17. Statistical analysis of canonical CRISPR assays for miR-98. Two-tailed unpaired t-tests were performed to compare wild-type and mutant signals at each concentration. Reported values include the difference ± standard error (Diff. ± SE) and 95% confidence intervals (95% CI).

**A**	**B**	**Mean (A)**	**Mean (B)**	**Diff. ± SE**	**95% CI**	**P value**	**t, df**
10nM	NC	59,640	-39.33	-59,680 ± 141.3	-60,072 to -59,287	<0.0001	t=422.4, df=4
1nM	NC	6,428	-39.33	-6,467 ± 326.5	-7,374 to -5,561	<0.0001	t=19.80, df=4
100pM	NC	2,625	-39.33	-2,665 ± 202.0	-3,225 to -2,104	0.0002	t=13.19, df=4
10pM	NC	1,099	-39.33	-1,138 ± 98.94	-1,413 to -863.4	0.0003	t=11.50, df=4
1pM	NC	1,082	-39.33	-1,122 ± 70.93	-1,319 to -924.7	<0.0001	t=15.81, df=4
100fM	NC	969.4	-39.33	-1,009 ± 38.79	-1,116 to -901.0	<0.0001	t=26.00, df=4
10fM	NC	470	-39.33	-509.4 ± 15.51	-552.4 to -466.3	<0.0001	t=32.84, df=4

Supplementary Table 18. PCR Ct values for $IDH1^{WT}$ and $IDH1^{R132H}$ targets across serial dilutions. Ct values were measured for wild-type ($IDH1^{WT}$) and mutant ($IDH1^{R132H}$) targets across concentrations from 1nM to 1aM. Reactions without detectable amplification are indicated as "–".

**Concentration**	**Target**	
	**$IDH1^{WT}$**	**$IDH1^{mut}$**
1nM	7.39	6.25
100pM	12.46	11.73
10pM	16.31	16.26
1pM	20.15	19.77
100fM	23.35	23.78
10fM	27.34	27.13
1fM	32.22	31.40
100aM	40.27	35.37
10aM	–	36.71
1aM	–	–
NC	–	–

Supplementary Table 19. SDS-CRISPR analysis of fresh-frozen non-glioma control tissues. Wild-type and mutant SDS-CRISPR assays were performed on DNA extracted from ten fresh-frozen non-glioma control samples (S1–S10). The table summarizes mean fluorescence signals and standard deviations (s.d.) for wild-type (WT) and mutant (mut) assays, along with the calculated mutant-to-wild-type ratios.

**Sample**	**WT assay**		**mut assay**		**Ratio**
	**Mean**	**s.d.**	**Mean**	**s.d.**	
S1	3,124.03	212.25	27.47	2.53	0.01
S2	24,862.44	1,606.91	81.12	2.31	0.00
S3	10,996.31	735.39	30.41	1.64	0.00
S4	15,270.19	683.58	31.26	5.63	0.00
S5	14,965.34	1,123.16	70.15	2.42	0.00
S6	24,173.42	1,922.35	45.27	2.72	0.00
S7	14,738.61	929.02	93.71	1.92	0.01
S8	4,023.27	187.94	4.76	2.20	0.01
S9	24,149.38	423.93	77.38	1.36	0.00
S10	10,896.72	575.94	19.88	0.92	0.00

Supplementary Table 20. Oligonucleotide sequences used in this study. This table lists all crRNA, activator, reporter, and primer sequences used across the different SDS-CRISPR and control assays.

Name	Sequence (5'→3')
Full-sized ssDNA (20 nt)	
Target	TTTGCCCCAGTGTTCCCCTA
B20-T>A	ATTGCCCCAGTGTTCCCCTA
B20-T>G	GTTGCCCCAGTGTTCCCCTA
B20-T>C	CTTGCCCCAGTGTTCCCCTA
B19-T>A	TATGCCCCAGTGTTCCCCTA
B19-T>G	TGTGCCCCAGTGTTCCCCTA
B19-T>C	TCTGCCCCAGTGTTCCCCTA
B18-T>A	TTAGCCCCAGTGTTCCCCTA
B18-T>G	TTGGCCCCAGTGTTCCCCTA
B18-T>C	TTCGCCCCAGTGTTCCCCTA
B17-G>A	TTTACCCCAGTGTTCCCCTA
B17-G>T	TTTTCCCCAGTGTTCCCCTA
B17-G>C	TTTCCCCCAGTGTTCCCCTA
B16-C>A	TTTGACCCAGTGTTCCCCTA
B16-C>T	TTTGTCCCAGTGTTCCCCTA
B16-C>G	TTTGGCCCAGTGTTCCCCTA
B15-C>A	TTTGCACCAGTGTTCCCCTA
B15-C>T	TTTGCTCCAGTGTTCCCCTA
B15-C>G	TTTGCGCCAGTGTTCCCCTA
B14-C>A	TTTGCCACAGTGTTCCCCTA
B14-C>T	TTTGCCTCAGTGTTCCCCTA
B14-C>G	TTTGCCGCAGTGTTCCCCTA
B13-C>A	TTTGCCCAAGTGTTCCCCTA
B13-C>T	TTTGCCCTAGTGTTCCCCTA
B13-C>G	TTTGCCCGAGTGTTCCCCTA
B12-A>T	TTTGCCCCTGTGTTCCCCTA
B12-A>G	TTTGCCCCGGTGTTCCCCTA
B12-A>C	TTTGCCCCCGTGTTCCCCTA
B11-G>A	TTTGCCCCAATGTTCCCCTA
B11-G>T	TTTGCCCCATTGTTCCCCTA
B11-G>C	TTTGCCCCACTGTTCCCCTA
B10-T>A	TTTGCCCCAGAGTTCCCCTA
B10-T>G	TTTGCCCCAGGGTTCCCCTA
B10-T>C	TTTGCCCCAGCGTTCCCCTA
B9-G>A	TTTGCCCCAGTATTCCCCTA
B9-G>T	TTTGCCCCAGTTTTCCCCTA
B9-G>C	TTTGCCCCAGTCTTCCCCTA
B8-T>A	TTTGCCCCAGTGATCCCCTA

B8-T>G	TTTGCCCCAGTGGTCCCCTA
B8-T>C	TTTGCCCCAGTGCTCCCCTA
B7-T>A	TTTGCCCCAGTGTACCCCTA
B7-T>G	TTTGCCCCAGTGTGCCCCTA
B7-T>C	TTTGCCCCAGTGTCCCCCTA
B6-C>A	TTTGCCCCAGTGTTACCCTA
B6-C>T	TTTGCCCCAGTGTTTCCCTA
B6-C>G	TTTGCCCCAGTGTTGCCCTA
B5-C>A	TTTGCCCCAGTGTTCACCTA
B5-C>T	TTTGCCCCAGTGTTCTCCTA
B5-C>G	TTTGCCCCAGTGTTCGCCTA
B4-C>A	TTTGCCCCAGTGTTCCACTA
B4-C>T	TTTGCCCCAGTGTTCCTCTA
B4-C>G	TTTGCCCCAGTGTTCCGCTA
B3-C>A	TTTGCCCCAGTGTTCCCATA
B3-C>T	TTTGCCCCAGTGTTCCCTTA
B3-C>G	TTTGCCCCAGTGTTCCCGTA
B2-T>A	TTTGCCCCAGTGTTCCCCAA
B2-T>G	TTTGCCCCAGTGTTCCCCGA
B2-T>C	TTTGCCCCAGTGTTCCCCCA
B1-A>T	TTTGCCCCAGTGTTCCCCTT
B1-A>G	TTTGCCCCAGTGTTCCCCTG
B1-A>C	TTTGCCCCAGTGTTCCCCTC
Split-ssDNA (Mutation on Pp)	
Pp-B1	ATAGGTGGTTTGCAA
Pp-B1-A>T	TTAGGTGGTTTGCAA
Pp-B1-A>G	GTAGGTGGTTTGCAA
Pp-B1-A>C	CTAGGTGGTTTGCAA
Pd-B1	TTTGCCCCAGTGTTCCCCT
Pp-B2	TATAGGTGGTTTGCA
Pp-B2-T>A	AATAGGTGGTTTGCA
Pp-B2-T>G	GATAGGTGGTTTGCA
Pp-B2-T>C	CATAGGTGGTTTGCA
Pd-B2	TTTGCCCCAGTGTTCCCC
Pp-B3	CTATAGGTGGTTTGC
Pp-B3-C>A	ATATAGGTGGTTTGC
Pp-B3-C>T	TTATAGGTGGTTTGC
Pp-B3-C>G	GTATAGGTGGTTTGC
Pd-B3	TTTGCCCCAGTGTTCCC
Pp-B4	CCTATAGGTGGTTTG
Pp-B4-C>A	ACTATAGGTGGTTTG
Pp-B4-C>T	TCTATAGGTGGTTTG
Pp-B4-C>G	GCTATAGGTGGTTTG

Pd-B4	TTTGCCCCAGTGTTCC
**Pp-**B5	CCCTATAGGTGGTTT
**Pp-**B5-C>A	ACCTATAGGTGGTTT
**Pp-**B5-C>T	TCCTATAGGTGGTTT
**Pp-**B5-C>G	GCCTATAGGTGGTTT
Pd**-B5**	TTTGCCCCAGTGTTC
**Pp-**B6	CCCCTATAGGTGGTT
**Pp-**B6-C>A	ACCCTATAGGTGGTT
**Pp-**B6-C>T	TCCCTATAGGTGGTT
**Pp-**B6-C>G	GCCCTATAGGTGGTT
**Pd-B6**	CTTTGCCCCAGTGTT
**Pp-**B7	TCCCCTA
**Pp-**B7-T>A	ACCCCTA
**Pp-**B7-T>G	GCCCCTA
**Pp-**B7-T>C	CCCCCTA
**Pd-B7**	CCTTTGCCCCAGTGT
**Pp-**B8	TTCCCCTATAGGTGG
**Pp-**B8-T>A	ATCCCCTATAGGTGG
**Pp-**B8-T>G	GTCCCCTATAGGTGG
**Pp-**B8-T>C	CTCCCCTATAGGTGG
**Pd-B8**	TCCTTTGCCCCAGTG
**Pp-**B9	GTTCCCCTATAGGTG
**Pp-**B9-G>A	ATTCCCCTATAGGTG
**Pp-**B9-G>T	TTTCCCCTATAGGTG
**Pp-**B9-G>C	CTTCCCCTATAGGTG
**Pd-B9**	ATCCTTTGCCCCAGT
**Pp-**B10	TGTTCCCCTATAGGT
**Pp-**B10-T>A	AGTTCCCCTATAGGT
**Pp-**B10-T>G	GGTTCCCCTATAGGT
**Pp-**B10-T>C	CGTTCCCCTATAGGT
**Pd-B10**	GATCCTTTGCCCCAG
**Pp-**B11	GTGTTCCCCTATAGG
**Pp-**B11-G>A	ATGTTCCCCTATAGG
**Pp-**B11-G>T	TTGTTCCCCTATAGG
**Pp-**B11-G>C	CTGTTCCCCTATAGG
**Pd-B11**	GGATCCTTTGCCCCA
**Pp-**B12	AGTGTTCCCCTATAG
**Pp-**B12-A>T	TGTGTTCCCCTATAG
**Pp-**B12-A>G	GGTGTTCCCCTATAG
**Pp-**B12-A>C	CGTGTTCCCCTATAG
**Pd-B12**	GGGATCCTTTGCCCC
**Pp-**B13	CAGTGTTCCCCTATA
**Pp-**B13-C>A	AAGTGTTCCCCTATA

**Pp-**B13-C>T	TAGTGTTCCCCTATA
**Pp-**B13-C>G	GAGTGTTCCCCTATA
**Pd-B13**	GGGGATCCTTTGCCC
**Pp-**B14	CCAGTGTTCCCCTAT
**Pp-**B14-C>A	ACAGTGTTCCCCTAT
**Pp-**B14-C>T	TCAGTGTTCCCCTAT
**Pp-**B14-C>G	GCAGTGTTCCCCTAT
**Pd-B14**	TGGGGATCCTTTGCC
**Pp-**B15	CCCAGTGTTCCCCTA
**Pp-**B15-C>A	ACCAGTGTTCCCCTA
**Pp-**B15-C>T	TCCAGTGTTCCCCTA
**Pp-**B15-C>G	GCCAGTGTTCCCCTA
**Pd-B15**	ATGGGGATCCTTTGC
**Pp-**B16	CCCCAGTGTTCCCCTA
**Pp-**B16-C>A	ACCCAGTGTTCCCCTA
**Pp-**B16-C>T	TCCCAGTGTTCCCCTA
**Pp-**B16-C>G	GCCCAGTGTTCCCCTA
**Pd-B16**	CATGGGGATCCTTTG
**Pp-**B17	GCCCCAGTGTTCCCCTA
**Pp-**B17-G>A	ACCCCAGTGTTCCCCTA
**Pp-**B17-G>T	TCCCCAGTGTTCCCCTA
**Pp-**B17-G>C	CCCCCAGTGTTCCCCTA
**Pd-B17**	ACATGGGGATCCTTT
**Pp-**B18	TGCCCCAGTGTTCCCCTA
**Pp-**B18-T>A	AGCCCCAGTGTTCCCCTA
**Pp-**B18-T>G	GGCCCCAGTGTTCCCCTA
**Pp-**B18-T>C	CGCCCCAGTGTTCCCCTA
**Pd-B18**	TACATGGGGATCCTT
**Pp-**B19	TTGCCCCAGTGTTCCCCTA
**Pp-**B19-T>A	ATGCCCCAGTGTTCCCCTA
**Pp-**B19-T>G	GTGCCCCAGTGTTCCCCTA
**Pp-**B19-T>C	CTGCCCCAGTGTTCCCCTA
**Pd-B19**	GTACATGGGGATCCT
Split-ssDNA (Mutation on Pd)	
Pd-**B13**-C>A	GGGATCCTTTGCCCA
Pd-**B13**-C>T	GGGATCCTTTGCCCT
Pd-**B13**-C>G	GGGATCCTTTGCCCG
Pd-**B12**-A>T	GGATCCTTTGCCCCT
Pd-**B12**-A>G	GGATCCTTTGCCCCG
Pd-**B12**-A>C	GGATCCTTTGCCCCC
Pd-**B11**-G>A	GATCCTTTGCCCCAA
Pd-**B11**-G>T	GATCCTTTGCCCCAT
Pd-**B11**-G>C	GATCCTTTGCCCCAC

Pd-**B10**-T>A	ATCCTTTGCCCCAGA
Pd-**B10**-T>G	ATCCTTTGCCCCAGG
Pd-**B10**-T>C	ATCCTTTGCCCCAGC
Pd-**B9**-G>A	TCCTTTGCCCCAGTA
Pd-**B9**-G>T	TCCTTTGCCCCAGTT
Pd-**B9**-G>C	TCCTTTGCCCCAGTC
Pd-**B8**-T>A	CCTTTGCCCCAGTGA
Pd-**B8**-T>G	CCTTTGCCCCAGTGG
Pd-**B8**-T>C	CCTTTGCCCCAGTGC
crRNA	
Full-sized crRNA	UAAUUUCUACUAAGUGUAGAUUAGGGGAACACUGGGGCAAA
crSplit-Type I-5’	UAAUUUCUACUAAGUGUAGAUUAGGGGAACA
crSplit-Type I -3’	CUGGGGCAAA
crSplit-Type II -Handle	UAAUUUCUACUAAGUGUAGAU
crSplit-Type II -Spacer	UAGGGGAACACUGGGGCAAA
2nd Target	
Full-sized crRNA	UAAUUUCUACUAAGUGUAGAUUAAUCCAUAGAUAAGCAUUCC
crSplit-Type II -Handle	UAAUUUCUACUAAGUGUAGAU
crSplit-Type II -Spacer	UAAUCCAUAGAUAAGCAUUCC
**Target-ssDNA**	GGAATGCTTATCTATGGATTA
**B**8-A>T	GGAATGCTTATCTTTGGATTA
**B**8-A>G	GGAATGCTTATCTGTGGATTA
**B**8-A>C	GGAATGCTTATCTCTGGATTA
**B9**-T>A	GGAATGCTTATCAATGGATTA
**B9**-T>G	GGAATGCTTATCGATGGATTA
**B9**-T>C	GGAATGCTTATCCATGGATTA
**B10**-C>A	GGAATGCTTATATATGGATTA
**B**10-C>T	GGAATGCTTATTTATGGATTA
**B**10-C>G	GGAATGCTTATGTATGGATTA
**B**11-T>A	GGAATGCTTAACTATGGATTA
**B**11-T>G	GGAATGCTTAGCTATGGATTA
**B**11-T>C	GGAATGCTTACCTATGGATTA
**B**12-A>T	GGAATGCTTTTCTATGGATTA
**B**12-A>G	GGAATGCTTGTCTATGGATTA
**B**12-A>C	GGAATGCTTCTCTATGGATTA
Pp-**B**8	ATGGATTATAAACAA
Pp-**B**8-A>T	TTGGATTATAAACAA
Pp-**B**8-A>G	GTGGATTATAAACAA
Pp-**B**8-A>C	CTGGATTATAAACAA
Pd-**B8**	AGGGAATGCTTATCT
Pp-**B**9	TATGGATTATAAACA

Pp-**B**9-T>A	AATGGATTATAAACA
Pp-**B**9-T>G	GATGGATTATAAACA
Pp-**B**9-T>C	CATGGATTATAAACA
Pd-**B9**	CAGGGAATGCTTATC
Pp-**B**10	CTATGGATTATAAAC
Pp-**B**10-C>A	ATATGGATTATAAAC
Pp-**B**10-C>T	TTATGGATTATAAAC
Pp-**B**10-C>G	GTATGGATTATAAAC
Pd-**B10**	ACAGGGAATGCTTAT
Pp-**B**11	TCTATGGATTATAAA
Pp-**B**11-T>A	ACTATGGATTATAAA
Pp-**B**11-T>G	GCTATGGATTATAAA
Pp-**B**11-T>C	CCTATGGATTATAAA
Pd-**B11**	AACAGGGAATGCTTA
Pp-**B**12	ATCTATGGATTATAA
Pp-**B**12-A>T	TTCTATGGATTATAA
Pp-**B**12-A>G	GTCTATGGATTATAA
Pp-**B**12-A>C	CTCTATGGATTATAA
Pd-**B12**	TAACAGGGAATGCTT
microRNA	
Handle	UAAUUUCUACUAAGUGUAGAU
Let-7a	UGAGGUAGUAGGUUGUAUAGUU
miR-98	UGAGGUAGUAAGUUGUAUUGUU
Let-7a Full-sized Assay	AACTATACAACCTACTACCTCA
miR-98 Full-sized Assay	AACAATACAACTTACTACCTCA
Let-7a-Pp	CTACTACCTCA
Let-7a-Pd	AACTATACAAC
miR-98-Pp	TTACTACCTCA
miR-98-Pd	AACAATACAAC
IDH1^{R132H} Detection	
mut-Gene Fragment	GAAATTTCTGGGCCATGAAAAAAAAAACATGCAAAATCACATT<br>ATTGCCAACATGACTTACTTGATCCCCATAAGCATGATGACCTA<br>TGATGATAGGTTTTACCCATCCACTCACAAGCCGGGGGATATTT<br>TTGCAGATAATGGCTTCTCTGAAGACCGTGCCACCCAGAATATT<br>TCGTATGGTGCCATTTGGTGATTTCC
WT- Gene Fragment	GAAATTTCTGGGCCATGAAAAAAAAAACATGCAAAATCACATT<br>ATTGCCAACATGACTTACTTGATCCCCATAAGCATGACGACCTA<br>TGATGATAGGTTTTACCCATCCACTCACAAGCCGGGGGATATTT<br>TTGCAGATAATGGCTTCTCTGAAGACCGTGCCACCCAGAATATT<br>TCGTATGGTGCCATTTGGTGATTTCC
IDH1-FP (134 bp)	CACATTATTGCCAACATGACTTACTTGATCCC
IDH1-RP (134 bp)	TTCTGGGTGGCACGGTCTTCAGAGAAGCCA
IDH1-RP (85 bp)	CCGGCTTGTGAGTGGATGGGTAAAACCTAT
Handle	UAAUUUCUACUAAGUGUAGAU

**Config-A-**WT-**Spacer**	AUAAGCAUGACGACCUAUGAU
**Config-A-**mut-**Spacer**	AUAAGCAUGAUGACCUAUGAU
**Config-B-**WT-**Spacer**	CGACCUAUGAUGAUAGGUUUU
**Config-B-**mut-**Spacer**	UGACCUAUGAUGAUAGGUUUU
**Config-C-**Spacer-	GACCUAUGAU
**Config-C-**WT-Handle+	UAAUUUCUACUAAGUGUAGAUAUAAGCAUGAC
**Config-C-**mut- Handle+	UAAUUUCUACUAAGUGUAGAUAUAAGCAUGAU
**Config-D-**WT-A>T-**Handle+**	UAAUUUCUACUAAGUGUAGAUAUAAGCAUGUC
**Config-D-**mut-A>T-**Handle+**	UAAUUUCUACUAAGUGUAGAUAUAAGCAUGUU
**Config-E-**G>C-**Spacer-**	CACCUAUGAU
**SDS-CRISPR-Assay-Spacer-**	ACCUAUGAUG
**SDS-CRISPR-**WT-**Assay -Handle+**	UAAUUUCUACUAAGUGUAGAUUAAGCAUGACG
**SDS-CRISPR-**mut-**Assay-Handle+**	UAAUUUCUACUAAGUGUAGAUUAAGCAUGAUG
qPCR-**p**air**1**-FWD	GCCAACATGACTTACTTGATCCC
qPCR-pair**1**-REV	TGGCACGGTCTTCAGAGAA
qPCR-pair**1**-PRB	/56-FAM/TTTACCCAT/ZEN/CCACTCACAAGCCGG/3IABkFQ/
qPCR-pair**2**-FWD-wt	TTGATCCCCATAAGCATGAC
qPCR-pair**2**-FWD-mut	TTGATCCCCATAAGCATGAT
qPCR-pair**2**-REV	GGTCTTCAGAGAAGCCATTATC
qPCR-pair**2**-PRB	/56-FAM/TTTACCCAT/ZEN/CCACTCACAAGCCGG/3IABkFQ/
HIV drug resistance	
HIV-M184-134bp	GAGCCTTTTAGAAAACAAAATCCAGACATAGTTATCTATCAATACATGGATGATTTGTATGTAGGATCTGACTTAGAAATAGGGCAGCATAGAACAAAAATAGAGGAGCTGAGACAACATCTGTTGAGGTGGG
HIV-M184V-134bp	GAGCCTTTTAGAAAACAAAATCCAGACATAGTTATCTATCAATACGTGGATGATTTGTATGTAGGATCTGACTTAGAAATAGGGCAGCATAGAACAAAAATAGAGGAGCTGAGACAACATCTGTTGAGGTGGG
M184-Spacer-	GGAUGAUUUG
M184-Handle+	UAAUUUCUACUAAGUGUAGAUAUCAAUACAU
M184V-Handle+	UAAUUUCUACUAAGUGUAGAUAUCAAUACGU
Others	
Reporter	/56-FAM/TTATT/3IABkFQ/
**LbCas12a**	MSKLEKFTNCYSLSKTLRFKAIPVGKTQENIDNKRLLVEDEKRAEDYKGVKKLLDRYYLSFINDVLHSIKLKNLNNYISLFRKKTRTEKENKELENLEINLRKEIAKAFKGNEGYKSLFKKDIIETILPEFLDDKDEIALVNSFNGFTTAFTGFFDNRENMFSEEAKSTSIAFRCINENLTRYISNMDIFEKVDAIFDKHEVQEIKEKILNSDYDVEDFFEGEFFNFVLTQEGIDVYNAIIGGFVTESGEKIKGLNEYINLYNQKTKQKLPKFKP

	LYKQVLSDRESLSFYGEGYTSDEEVLEVFRNTLNKNSEIFSSIKKL<br>EKLFKNFDEYSSAGIFVKNGPAISTISKDIFGEWNVIRDKWNAEY<br>DDIHLKKKAVVTEKYEDDRRKSFKKIGSFSLEQLQEYADADLSV<br>VEKLKEIIIQKVDEIYKVYGSSEKLFDADFVLEKSLKKNDAVVAI<br>MKDLLDSVKSFENYIKAFFGEGKETNRDESFYGDFVLAYDILLKV<br>DHIYDAIRNYVTQKPYSKDKFKLYFQNPQFMGGWDKDKETDYR<br>ATILRYGSKYYLAIMDKKYAKCLQKIDKDDVNGNYEKINYKLLP<br>GPNKMLPKVFFSKKWMAYYNPSEDIQKIYKNGTFKKGDMFNLN<br>DCHKLIDFFKDSISRYPKWSNAYDFNFSETEKYKDIAGFYREVEE<br>QGYKVSFESASKKEVDKLVEEGKLYMFQIYNKDFSDKSHGTPNL<br>HTMYFKLLFDENNHGQIRLSGGAELFMRRASLKKEELVVHPANS<br>PIANKNPDNPKKTTTLSYDVYKDKRFSEDQYELHIPIAINKCPKNI<br>FKINTEVRVLLKHDDNPYVIGIDRGERNLLYIVVVDGKGNIVEQY<br>SLNEIINNFNGIRIKTDYHSLLDKKEKERFEARQNWTSIENIKELK<br>AGYISQVVHKICELVEKYDAVIALEDLNSGFKNSRVKVEKQVYQ<br>KFEKMLIDKLNYMVDKKSNPCATGGALKGYQITNKFESFKSMST<br>QNGFIFYIPAWLTSKIDPSTGFVNLLKTKYTSIADSKKFISSFDRIM<br>YVPEEDLFEFALDYKNFSRTDADYIKKWKLYSYGNRIRIFRNPKK<br>NNVFDWEEVCLTSAYKELFNKYGINYQQGDIRALLCEQSDKAFY<br>SSFMALMSLMLQMRNSITGRTDVDFLISPVKNSDGIFYDSRNYEA<br>QENAILPKNADANGAYNIARKVLWAIGQFKKAEDEKLDKVKIAIS<br>NKEWLEYAQTSVKH
**HIV**	MSLPGRWKPKMIGGIGGFIKVRQYDQILIEICGHKAIGTVLVGPTP<br>VNIIGRNLLTQIGCTLNFPISPIETVPVKLKPGMDGPKVKQWPLTE<br>EKIKALVEICTEMEKEGKISKIGPENPYNTPVFAIKKKDSTKWRKL<br>VDFRELNKRTQDFWEVQLGIPHPAGLKKKKSVTVLDVGDAYFSV<br>PLDEDFRKYTAFTIPSINNETPGIRYQYNVLPQGWKGSPAIFQSSM<br>TKILEPFRKQNPDIVIYQYMDDLYVGSDLEIGQHRTKIEELRQHLL<br>RWGLTTPDKKHQKEPPFLWMGYELHPDKWTVQPIVLPEKDSWT<br>VNDIQKLVGKLNWASQIYPGIKVRQLCKLLRGTKALTEVIPLTEE<br>AELELAENREILKEPVHGVYYDPSKDLIAEIQKQGQGQWTYQIY<br>QEPFKNLKTGKYARMRGAHTNDVKQLTEAVQKITTESIVIWGKT<br>PKFKLPIQKETWETWWTEYWQATWIPEWEFVNTPPLVKLWYQL<br>EKEPIVGAETFYVDGAANRETKLGKAGYVTNRGRQKVVTLTDT<br>TNQKTELQAIYLALQDSGLEVNIVTDSQYALGIIQAQPDQSESELV<br>NQIIEQLIKKEKVYLAWVPAHKGIGGNEQVDKLVSAGIRKVLFLD<br>GIDKAQDEHEKYHSNWRAMASDFNLPPVVAKEIVASCDKCQLK<br>GEAMHGQVDCSPGIWQLDCTHLEGKVILVAVHVASGYIEAEVIPA<br>ETGQETAYFLLKLAGRWPVKTIHTDNGSNFTGATVRAACWWAGI<br>KQEFGIPYNPQSQGVVESMNKELKKIIGQVRDQAEHLKTAVQMA<br>VFIHNFKRKGGIGGYSAGERIVDIIATDIQTKELQKQITKIQNFRVY<br>YRDSRNPLWKGPAKLLWKGEGAVVIQDNSDIKVVPRRKAKIIRD<br>YGKQMAGDDCVASRQDED

References

[1] J. Abramson, J. Adler, J. Dunger, R. Evans, T. Green, A. Pritzel, O. Ronneberger, L. Willmore, A. J. Ballard, J. Bambrick, *Nature* **2024**, *630*, 493-500.

[2] Z. Li, X. Ding, K. Yin, L. Avery, E. Ballesteros, C. Liu, *Biosensors and Bioelectronics* **2022**, *199*, 113865.
